# A New Smoothened Antagonist Bearing the Purine Scaffold Shows Antitumour Activity In Vitro and In Vivo

**DOI:** 10.3390/ijms22168372

**Published:** 2021-08-04

**Authors:** Ana María Zárate, Christian Espinosa-Bustos, Simón Guerrero, Angélica Fierro, Felipe Oyarzún-Ampuero, Andrew F. G. Quest, Lucia Di Marcotullio, Elena Loricchio, Miriam Caimano, Andrea Calcaterra, Matías González-Quiroz, Adam Aguirre, Jaime Meléndez, Cristian O. Salas

**Affiliations:** 1 Departamento de Química Orgánica, Facultad de Química y de Farmacia, Pontificia Universidad Católica de Chile, Vicuña Mackenna 4860, Macul, Santiago 702843, Chile; amzarate@uc.cl (A.M.Z.); afierroh@uc.cl (A.F.); 2 Departamento de Farmacia, Facultad de Química y de Farmacia, Pontificia Universidad Católica de Chile, Vicuña Mackenna 4860, Macul, Santiago 702843, Chile; ccespino@uc.cl; 3 Advanced Center for Chronic Diseases (ACCDiS), Universidad de Chile, Sergio Livingstone 1007, Independencia, Santiago 8380492, Chile; simon.daiblogt@gmail.com (S.G.); foyarzuna@ciq.uchile.cl (F.O.-A.); aquest@med.uchile.cl (A.F.G.Q.); 4 Instituto de Investigación Interdisciplinar en Ciencias Biomédicas, Facultad de Ciencias de la Salud, Universidad SEK (I3CBSEK), Fernando Manterola 0789, Providencia, Santiago 7520317, Chile; 5 Departamento de Ciencias y Tecnología Farmacéuticas, Facultad de Ciencias Químicas y Farmacéuticas, Universidad de Chile, Santos Dumont 964, Independencia, Santiago 8380494, Chile; 6 Laboratorio de Comunicaciones Celulares, Centro de Estudios en Ejercicio, Metabolismo y Cáncer (CEMC), Program of Cellular and Molecular Biology, Instituto de Ciencias Biomédicas (ICBM), Facultad de Medicina, Universidad de Chile, Independencia 1027, Santiago 8380453, Chile; 7 Laboratory Affiliated to Insituto Pasteur Italia, Fondazione Cenci Bognetti, Department of Molecular Medicine, Sapienza University of Rome, Viale Regina Elena 291, 00161 Rome, Italy; lucia.dimarcotullio@uniroma1.it; 8 Center For Life Nano Science@Sapienza, Istituto Italiano di Tecnologia, Viale Regina Elena 291, 00161 Rome, Italy; elena.loricchio@iit.it; 9 Department of Molecular Medicine, Sapienza University of Rome, Viale Regina Elena 291, 00161 Rome, Italy; miriam.caimano@uniroma1.it; 10 Department of Chemistry and Technology of Drugs, Sapienza University of Rome, Piazzale Aldo Moro 5, 00185 Rome, Italy; andrea.calcaterra@uniroma1.it; 11 Program of Cellular and Molecular Biology, Institute of Biomedical Sciences, Universidad de Chile, Independencia 1027, Santiago 8380453, Chile; mugonzalez@uc.cl; 12 Laboratorio de Medicina Traslacional, Fundación Arturo López Pérez, Rancagua 878, Lower Fifth Floor, Providencia, Santiago 8320000, Chile; adam.aguirre@falp.org

**Keywords:** Hedgehog signalling pathway, smoothened receptor antagonists, purine derivatives, docking studies, in vivo assays

## Abstract

The Smoothened (SMO) receptor is the most druggable target in the Hedgehog (HH) pathway for anticancer compounds. However, SMO antagonists such as vismodegib rapidly develop drug resistance. In this study, new SMO antagonists having the versatile purine ring as a scaffold were designed, synthesised, and biologically tested to provide an insight to their mechanism of action. Compound **4s** was the most active and the best inhibitor of cell growth and selectively cytotoxic to cancer cells. **4s** induced cell cycle arrest, apoptosis, a reduction in colony formation and downregulation of *PTCH* and *GLI1* expression. BODIPY-cyclopamine displacement assays confirmed **4s** is a SMO antagonist. In vivo, **4s** strongly inhibited tumour relapse and metastasis of melanoma cells in mice. In vitro, **4s** was more efficient than vismodegib to induce apoptosis in human cancer cells and that might be attributed to its dual ability to function as a SMO antagonist and apoptosis inducer.

## 1. Introduction

The Hedgehog (HH) signalling pathway plays a crucial role in early stages of embryo development. The transduction signal, induced by HH ligands (SHH, IHH and DHH), regulates proliferation and cell differentiation in the physiological maturation of new tissues [1,2,3]. At the same time, aberrant activation of this pathway is linked to the occurrence of malignant transformation in different cancers that affect humans [4,5,6]. When HH ligands binds to Patched (PTCH), a 12-pass transmembrane protein which represses the pathway, the inhibition exerted by PTCH on the co-receptor Smoothened (SMO) is relieved. As a result, the active SMO triggers a downstream signalling cascade, which leads to the activation of GLI transcription factors (Gli1, Gli2 and Gli3) that translocate into the nucleus and induce the expression of their target genes including *GLI1* and *PTCH1*, among others which are pro-tumorigenic [7,8]. Nevertheless, in several cancers including basal cell carcinoma (BCC) and medulloblastoma (MB), HH pathway is constitutively activated and related to ligand-independent mechanism, due to somatic mutations in *PTCH1*, *SMO* or *SUFU* genes [9]. Conversely, ligand-dependent activation has been shown in other malignancies, such as melanoma, pancreatic, lung, breast, renal, and colorectal cancers [2,6]. In all cases, the resulting increase in HH signalling activity is responsible for an increase in cell proliferation along with neoplastic transformation.

**Figure 1:**
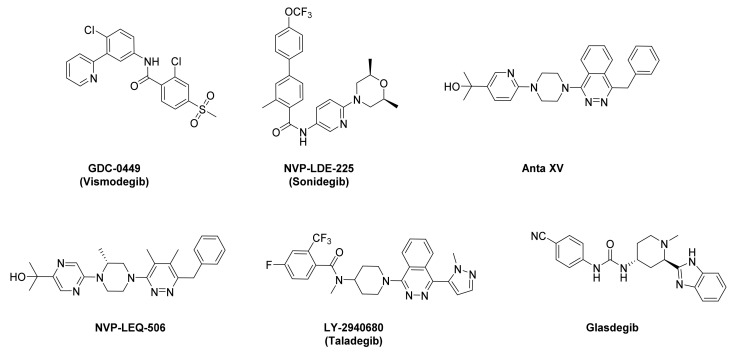
Chemical structure of SMO antagonists approved by the FDA or in clinical phase studies.

Anticancer therapy based on the ability to inhibit HH signalling at the upstream level on SMO receptor has passed clinical scrutiny. Two Smo antagonists: vismodegib [10,11] and sonidegib [12] (Figure 1), have been approved by the FDA in 2012 and 2015 [13,14], respectively, for the treatment of metastatic and locally advanced BCC or MB. Although both FDA-approved SMO antagonists were initially successful in the treatment of BCC or MB, they rapidly caused drug resistance due to acquired mutations within the SMO-transmembrane domain, which prevent the binding of the drugs [15,16]. Despite their limitations, SMO antagonists are in new clinical trials for various cancers, either as mono- or combined therapy [15,16]. In addition, a Pfizer oral inhibitor of the HH signalling pathway, glasdegib (DAURISMO™, Figure 1), was approved in November 2018 in the USA for the treatment of recently diagnosed acute myeloid leukaemia (AML) in combination with low-dose of cytarabine [17,18,19]. 

**Figure 2:**
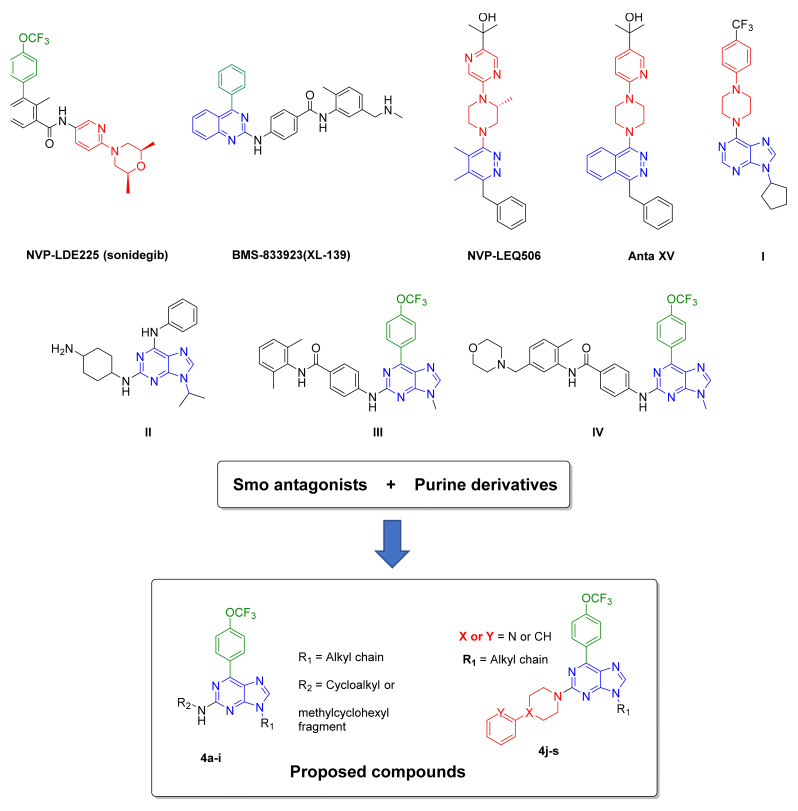
Chemical structures of SMO antagonists, biologically active purine derivatives with potential as anticancer agents, and purine derivatives designed to serve as SMO antagonists. In colour are shown the fragments considered in the design of these purine derivatives: in blue a heterocyclic core, in red an aryl-piperazine portion and in green the trifluoromethoxy moiety.

Efforts to identify new SMO antagonists have yielded several potential candidates [20], such as the following phthalazine derivatives such as Anta XV, NVP-LEQ506 and taladegib (Figure 1) [21,22]. Taladegib is currently undergoing phase II clinical studies at Eli Lilly for new oesophageal cancer therapies [23,24]. Finally, in exploring new SMO antagonists with novel structural patterns, the purine ring has emerged as an interesting scaffold because all living organisms have biological molecules that have this heterocyclic structure and play major roles in signalling pathways [25,26,27,28]. In fact, compounds **I** and **II** are examples of synthetic purine derivatives with antitumour activities (Figure 2) [29,30]. Along with the purine scaffold, compound **I** has a piperazinyl group (also present in Anta XV and NVP-LEQ506), and a diminutive and inflexible heterocyclic backbone, which was applied in the design of various anticancer drugs [31]. Compounds **III**–**IV** (Figure 2) exemplify 2,6,9-trisubstituted purine derivatives which stood out as SMO antagonists in research conducted by Zhang et al. [32]. Both purine derivatives showed IC_50_ values in the nanomolar range in GLI-luciferase reporter assays. From the chemical perspective, **III** and **IV** have a trifluoromethylphenyl group at C-6 of the purine ring (in sonidegib), a methyl substitution on *N*-9 and a variable aniline moiety on C-2.

In this study, we took the purine scaffold and some fragments from other SMO antagonists, including those mentioned above and BMS-833923 [33,34], as the starting point to design a new series of purine derivatives that would inhibit the HH pathway upon binding to SMO receptor (Compounds **4a**–**4s**, Figure 2). Subsequently, to evaluate the activity of the new compounds on HH signalling, we tested them on HH-dependent cancer cell lines and ranked the most promising compounds in order of selectivity and cytotoxicity. Selected compounds were further studied to determine the type of cell death by flow cytometry. Quantitative Real Time PCR analyses were carried out to evaluate the effect on the downregulation of HH-target genes expression. Gli1-transcription functional assays and BODIPY-cyclopamine displacement assays were also performed for the eventual hit compound. Our work also included in silico studies using docking analysis that allowed us to analyse the binding mode of some purine derivatives in the SMO binding pocket. Finally, using a syngeneic mouse model, we demonstrated that the most promising compound elicits a strong inhibitory effect on tumour relapse and metastasis in vivo. Our results provide a platform for designing new SMO antagonists that may prove to be stronger and more selective anticancer agents.

## 2. Results

### 2.1. Synthesis

**Scheme 1:**
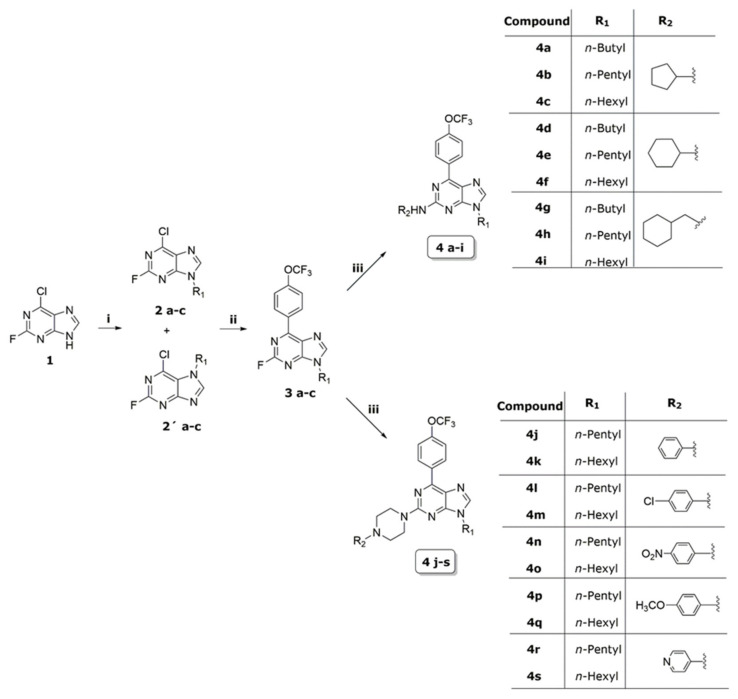
Synthesis of purine derivatives **4a**–**4s**. *Reagents and conditions*: (**i**) Alkyl halides, K_2_CO_3_, DMF, r.t., 12 h (51–58%); (**ii**) 4-Trifluoromethoxyphenylboronic acid, Pd(PPh_3_)_2_Cl_2_, K_2_CO_3_ 2 M, 1,4-dioxane (41–66%); (**iii**) amines or piperazine derivatives, DIPEA, *n*-BuOH, 110 °C, 12 h (77–97%).

The structures and synthesis of the new 2,6,9-trisubstituted purines **4a**–**4s** appear in Scheme 1. Nineteen compounds based on the chemical structures of well-known SMO ligands were synthesised using a three-step synthesis starting from 2-chloro-6-fluoro-purine (**1**). The initial step involved alkylating **1** with some alkyl halides under basic conditions to yield the *N*-9 and *N*-7-alkylated purine regioisomers **2a**–**2c**:**2a′**–**2c′**, in most cases in a 4:1 ratio, as we reported previously [35,36]. Later, the regioisomers **2a**–**2c** were substituted in position C-6 with trifluoromethoxyphenylboronic acid to obtain compounds like-**3** by a regiospecific Suzuki cross coupling reaction [35,36,37]. Finally, a nucleophilic substitution (SNAr) on C-2 was carried out using several amines or piperazine derivatives to obtain **4a**–**4s** in high yields (77–97%). The synthetic strategy employed was an effective method to synthesise compounds used for research. The chemical structures of the synthesised compounds were confirmed according to their spectral properties. ^1^H NMR and ^13^C NMR spectra and HRMS are reported in the Supplementary Materials section. 

### 2.2. Cytotoxic Studies

In order to test the activity of 2,6,9-trisubstituted purines in vitro as SMO antagonists, we carried out cytotoxicity studies using 19 compounds (**4a**–**4s**) in both HH-dependent (Daoy [38,39,40]), (HT-29 and HCT-116 cells [41,42]) and HH-independent (H1975, AsPC-1, BxPC-3) cell lines reported to show a moderate to high expression of SMO receptor and HH signalling components [43,44,45,46] and in HEK293 cells as control. Etoposide and 5-fluorouracil (5-FU) were used as anticancer agents to compare cytotoxicity. Results from our studies revealed that 7 of the 19 compounds showed a cytotoxic activity greater than 50% after 72 h of treatment in an MTT assay (Supplementary Materials, Table S1). Of note, compounds **4r** and **4s** showed the highest degree of cytotoxicity (more than 80%) at 50 µM in most of the tumour cell lines tested, but less than 50% in HEK293 control cells, highlighting the specificity of these small molecules. 

In order to estimate the IC_50_ and selectivity index values of these seven selected compounds, including **4r** and **4s**, four serial dilutions (from 0.05 to 50 µM) of each sample were evaluated after 72 h using etoposide, 5-FU and cisplatin as positive controls. Vismodegib, a well-known SMO antagonist, was included for comparison. Gemcitabine was also used as a positive control for the two pancreatic tumour cell lines (AsPC-1 and BxPC-3) included in the screening panel.

Table 1 shows the IC_50_ cytotoxicity values of seven compounds, including **4r** and **4s**, on six tumour cell lines and HEK293 control cells. Results revealed the lowest IC_50_ values for **4s**, ranging between 1.3 and 15 µM depending on the tumour cell line. These values represent approximately a 40-fold increase in cytotoxicity compared to vismodegib and a 7-fold increased activity compared to anticancer agents used clinically, such as etoposide, cisplatin, 5-FU or gemcitabine, as appropriate for each type of tumour cell line. In addition, **4s** was 40 times more selective for cancer cells compared to HEK293 control cells. Effects of **4s** on HEK293 cells were observed at concentrations above 50 µM. In fact, the selectivity index of **4s** was 12 and 29 for the pancreatic cell lines BxPC-3 and AsPC-1, respectively, 38 for HCT116, 3.3 for HT29 and 33 for H1975 cells. It is remarkable that compound **4s** was more effective than vismodegib in all tumour cell lines assayed. Although **4r** showed considerable cytotoxicity, its effect was lower than **4s**, with higher IC_50_ and lower SI values. In Daoy cells, compounds **4r** and **4s** had IC_50_ values of 1.5 and 6.5 µM, respectively, and a selectivity index greater than 6, which indicates that, although SMO is constitutively active in this cell type, **4s** is still effective at inducing significant cytotoxicity. Thus, one compound (**4s**) that is structurally simpler than other available SMO antagonists on the market showed the highest cytotoxic effect (IC_50_ of 1.3–15 µM) in all tumour cell lines and was equally effective in both HH-dependent and independent cell lines assayed. From here on we focused on **4s** to explore the molecular mechanism of these effects in cancer in HH-dependent cell lines with a high expression of SMO [44].

### 2.3. Effect of ***4s*** in Proliferation, Cell Cycle, Apoptosis and Colony Formation of Cancer Cells

**Figure 3:**
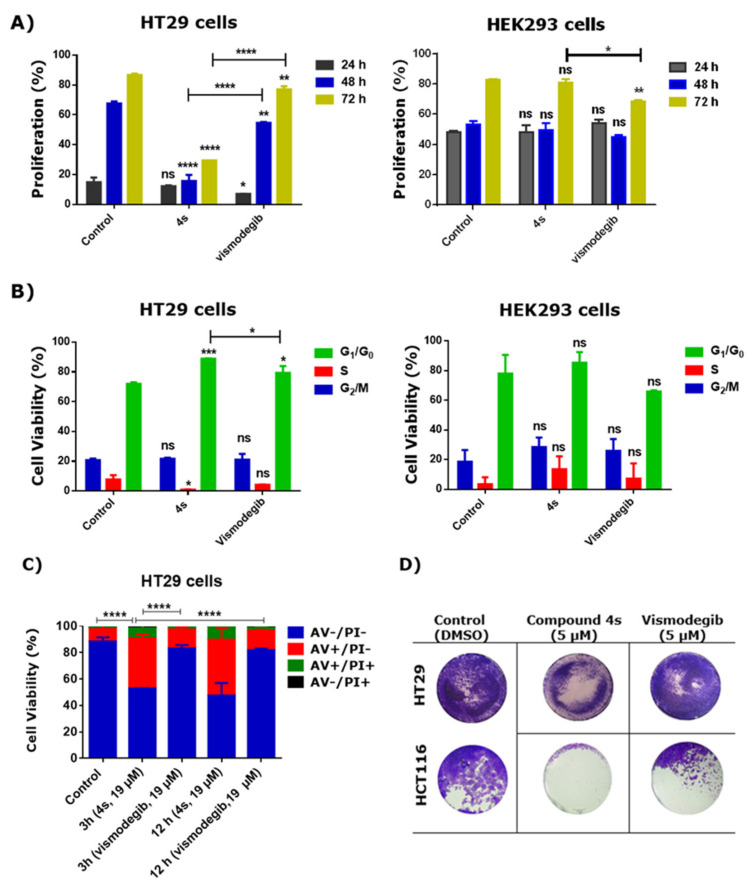
Effects of **4s** on cell proliferation, cell cycle arrest, apoptosis, and colony formation of cancer cells. HT29 and HEK293 cell lines were treated with **4s** or vismodegib for 3, 12, 24, 48 and/or 72 h (19 µM), depending on the analysis, for (**A**) anti-proliferative effects determined by flow cytometry upon staining cells with 5 µM CFSE (control); (**B**) DNA content determined by flow cytometry; (**C**) apoptosis determined by flow cytometry after staining with annexin V-FITC/PI; (**D**) effects on colony formation of cancer cells. Cells were treated with **4s** or vismodegib (5 µM) for 14 d. Results of (**A**–**C**) are presented as mean ± SD of two separate experiments. (*) *p* < 0.05, (**) *p* < 0.01, (***) *p* < 0.001, (****) *p* < 0.0001 vs. control, ns = non-significant. Data analysis was performed via a 2-way ANOVA Tukey’s multiple comparisons test.

To shed light on different effects shown by **4s** in cancer cells, proliferation rate and cell cycle arrest were compared in HT29 and HEK293 control cells (Figure 3A,B). For proliferation assays, cells were plated and treated at sub-confluency with a fixed concentration of **4s** (19 µM) or using vismodegib at same concentration. Cell death was close to 50% after 24, 48 and 72 h as assessed using the carboxyfluorescein diacetate succinimidyl ester (CFSE) fluorescent probe. Incubation with **4s** (19 µM) significantly reduced HT29 cell proliferation after 48 h of treatment, while no effect was shown by the control HEK293 cells. The same effects were observed in Daoy cells (data not shown). On the other hand, vismodegib had less effect on reducing proliferation than **4s** in either HT29 or HEK293 cells. Thus, **4s** inhibits cell growth more effectively than vismodegib after 48 h in both cell lines (Figure 3A). Taking into account that apoptotic mechanisms are related to the arrest of the G_1_/S boundary cell cycle, and furthermore, cycle progression is a crucial mechanism to control the growth of cancerous cells, we looked to further dissect the mechanism of **4s** action. Cell suspensions underwent treatment with the same concentrations (19 µM) of either **4s** or vismodegib for 24 h and effects on the cell division cycle were assessed in HT29 and HEK293 cell lines. We analysed the cell cycle distribution of PI-stained cells by flow cytometry. HT29 cells treated with **4s** showed a significant decrease of 12% in S phase and the consequent increase of the G_1_/G_0_ phase by 24% compared to the control (Figure 3B). We did not observe any changes in HEK293 cells after treatment with **4s**. A concomitant tendency towards increases in 10% of the G_1_/G_0_ population was also detected in HT29 cells treated with vismodegib (Figure 3B).

Apoptosis plays a major role in maintaining the homeostasis of cells. Cellular shrinkage and DNA fragmentation at the molecular level are characteristics acquired during apoptosis. Therefore, we performed flow cytometric analysis to determine if **4s** could induce apoptosis in tumour cell lines. To this end, **4s**-treated cells were stained with Annexin V-FITC and/or PI and analysed by flow cytometry. Dose–response effects were assessed using **4s** at concentrations under 50 µM (1.5, 3.0, 6.25, 19, 25 and 50 µM) for 72 h. Indeed, the results showed that **4s** treatment induced apoptosis in a dose-dependent manner in all cancer cell lines (HH-dependent and HH-independent) assayed in this study (Supplementary Materials, Figure S1A). 

To understand the ability of compound **4s** (high SI and minimum IC_50_ values) to promote cell death, we performed an Annexin V-FITC/PI assay at times earlier than 24 h. HT29 cells were treated with **4s** at 19 µM for 3 or 12 h and compared to cells treated with vismodegib. As shown in Figure 3C and Supplementary Materials Figure S1B, **4s** diminished cell viability (from around 95.7–33.8%) and increased early apoptosis (II) compared to untreated control cells (2.7 and 64.6%, respectively). Vismodegib showed no statistically significant differences in early apoptosis compared to control cells. In addition, Figure 3C reveals an increase in early and late apoptosis (II and III) after 3 h at 19 µM, indicating that **4s** can induce apoptosis in a time-dependent manner.

Finally, additional support for the anticancer effects of the **4s** was obtained using colony formation assays. HT29 and HT116 cells were seeded on plastic at low-density and were treated with either vehicle (DMSO), **4s** (5 μM) or vismodegib (5 μM). For cells treated with DMSO, colony formation was detected after 14 d (Figure 3D). Remarkably, **4s** totally inhibited the formation of colonies at 5 µM in HCT116 and to a lesser extent in HT-29 cells because of a reduction in cell proliferation. In contrast, vismodegib was less effective than **4s** at the same concentration in both cancer cell lines. Thus, **4s** inhibits more effectively HCT116 and HT29 colony formation than vismodegib. Taken together, our data indicate that **4s** shows an anti-proliferative activity, induces cell cycle arrest and apoptosis, and inhibits colony formation of multiple cancer cell types.

### 2.4. Effect of ***4s*** on the Expression of HH Signalling Pathway Target Genes

**Figure 4:**
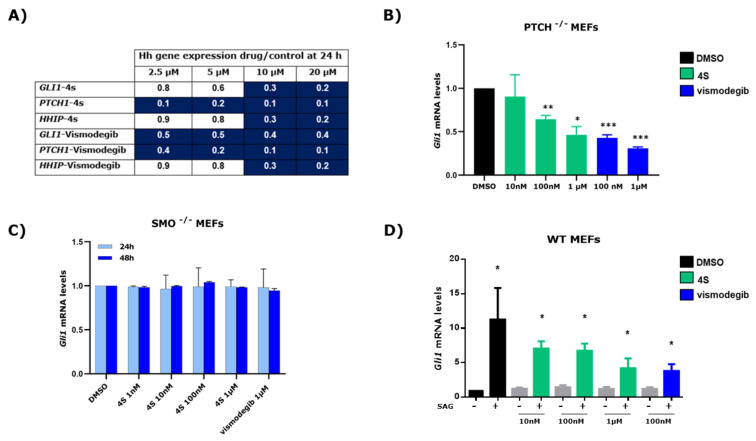
Effect of **4s** on the expression of HH target genes. (**A**) Heat map of mRNA levels in human colon cancer HT29 cells as determined by qRT-PCR for a 3-gene HH signature. All values are ratios of experimental over control (treated only with DMSO) data, having previously normalised individual gene expression Ct values to those of housekeeping genes. Samples treated with drugs were collected 24 h after treatment. Expression changes are highlighted as follows: Blue: repression ≤ 0.5. White: no gene repression with values near 1. Results were obtained from three independent experiments and expressed as the mean ± the standard deviation. (**B**) *Gli1* mRNA levels in *Ptch*^−/−^ KO cells treated with **4s** for 24 h. Vismodegib was used as a control. mRNAs were extracted and *Gli1* mRNA levels were determined by qRT–PCR and normalised to the endogenous *Hprt* control. Data are presented as the mean ± SD of three independent experiments. (*) *p* < 0.05, (**) *p* < 0.01, (***) *p* < 0.001 vs. DMSO (vehicle). Data were analysed with a parametric *t*-test. (**C**) *Gli1* mRNA levels in *Smo*^−/−^ MEF cells treated with **4s** for 24 h and/or 48 h. Vismodegib was used as a control. mRNAs were extracted and *Gli1* mRNA levels were determined by qRT–PCR and normalised to the endogenous *Hprt* control. Data are presented as the mean ± SD of three independent experiments. (**D**) *Gli1* mRNA levels in WT MEFs treated for 24 h with DMSO or SAG 200 nM in presence or absence of compound **4s** at the indicated concentrations. Vismodegib was used as a control. mRNAs were extracted and *Gli1* mRNA levels were determined by qRT–PCR and normalised to the endogenous *Hprt* control. Data are presented as the mean ± SD of three independent experiments. (*) *p* < 0.05. Data were analysed with a parametric *t*-test.

To verify that **4s** acts on the HH pathway, the expression of its target genes was evaluated by qRT-PCR in HT29 cells treated with **4s** or vismodegib, as a control. Our results showed that over a wide range of concentrations (2.5–50 µM), **4s** treatment reduced *GLI1*, *PTCH1* and *HHIP* mRNA levels (Figure 4A) more effectively than vismodegib. To provide additional support to the ability of compound **4s** to target the HH pathway, increasing concentrations of compound **4s** significantly reduced mRNA levels of *GLI1*, the final effector of HH signalling in genetically defined *Ptch1*^−/−^ mouse embryonic fibroblasts (*Ptch1*^−/−^ MEFs) (Figure 4B), in which constitutive activation of the Hh pathway is the consequence of the loss of repressive receptor *Ptch1* gene. Conversely, in MEFs lacking the Smo receptor (*Smo*^−/−^ MEFs), both compound **4s** and vismodegib did not affect the mRNA levels of GLI1 (Figure 4C). It is worth noting that although in *Smo*^−/−^ MEFs the levels of the *Gli1* mRNA are lower than wildtype cells, the RNA levels are detectable and can be pharmacologically modulated [47]. SAG is a known SMO agonist and directly binds to SMO and can block SMO inhibition by cyclopamine. We wanted to explore whether the antagonistic effect of **4s** on the SMO receptor at increasing concentrations can displace the SAG agonist effect in WT MEF cells. The results showed that **4s** at a concentration of 10 µM inhibited *Gli-1* expression in these cells when compared to WT MEF cells stimulated with SAG only. The inhibition of *Gli-1* expression depends upon **4s** concentration in the presence of a constant concentration of SAG. These results reinforce the evidence that **4s** acts as an SMO antagonist in WT MEF cells (Figure 4D). Overall, these data suggest that **4s** inhibits the HH signalling pathway by acting on the SMO receptor and regulates cancer cells characteristics by inhibiting various components of the HH pathway.

To further evaluate the effect of **4s** on HH signalling, we carried out a transcriptional functional assay in HCT116 cells, stably expressing a GLI1-responsive luciferase reporter and the pRL-TK renilla reporter for normalisation, treated for 24 h with **4s**, vismodegib and purmorphamine [48] as a positive control. Compound **4s**, as well as vismodegib, did not affect basal *GLI1* transcription activity. On the other hand, both **4s** and vismodegib effectively reduced the activation induced by the agonist purmorphamine (Supplementary Materials, Figure S2). These results indicate that **4s** inhibits *GLI1* transcriptional activity in HCT116 cells though it remains to be shown whether **4s** inhibits the GLI1-DNA binding activity as vismodegib does [49].

### 2.5. Study of ***4s*** as Antagonist of the SMO Receptor

**Figure 5:**
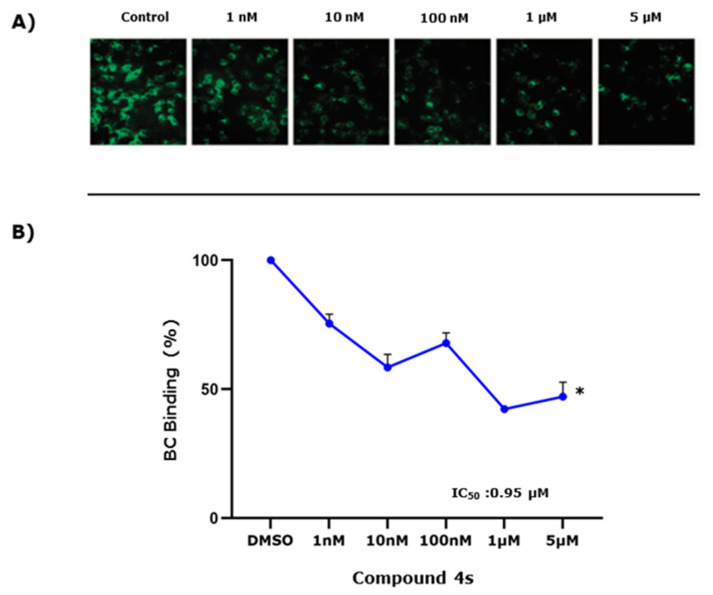
Binding of **4s** to HEK293T cells incubated with the fluorescent compound BC in a competition assay. (**A**) Fluorescence photomicrographs of the competitive binding of BC (green) to HEK293T cells treated with increasing concentrations of **4s** or BODIPY only (control). (**B**) The concentration–response curve shows the percentage of BC binding to WT SMO after treatment with compound **4s**. IC_50_ for binding to WT SMO = 0.95 µM. Data indicate the average ± SD of three separate experiments. (*) *p* < 0.05 vs. control. Data are the average BC intensity of five fluorescence microscopy images.

To confirm that **4s** acts directly on the SMO receptor, we performed a BODIPY-cyclopamine (BC) displacement assay using a fluorescent derivative of cyclopamine that interacts with SMO. To this end, HEK293T cells were transfected with a vector encoding WT SMO and incubated with BC at increasing concentrations of **4s**. As shown in Figure 5, **4s** reduced BC binding in a dose-dependent manner.

Vismodegib resistance is associated with SMO mutations (mainly in amino acid residue D473) [20,50]. For this reason, major efforts have been made to identify specific molecules able to overcome drug resistance. Therefore, we evaluated the ability of compound **4s** to bind the SMO D473H mutant. To accomplish this, we carried out the BC displacement assay with a vector encoding the SMO D473H mutant, which confers resistance following vismodegib treatment (Supplementary Materials, Figure S3). Vismodegib displayed a binding affinity of 0.00762 µM in HEK293T cells carrying the SMO WT vector; however, in cells carrying the SMO-D473H vector, its affinity was reduced around 1000-fold with an IC_50_ of 10.450 µM [51]. These results support previous data from patients affected with BCC in whom vismodegib therapies resulted in a poor prognosis. On the other hand, incubation of cells expressing SMO-D473H with **4s** resulted in a dose-dependent inhibition of binding to the SMO-D473H mutant receptor. Nevertheless, in these cells **4s** exhibited toxicity above 5 µM, so we were unable to determine an IC_50_ value. It was only possible to suggest that **4s** decreased about 25% of its binding affinity with SMO-D473H mutant compared to WT SMO (Supplementary Materials, Figure S3). In this way, **4s** behaved as a SMO antagonist where the use of binding assays with BODIPY-cyclopamine clearly demonstrated that the binding site of **4s** is SMO.

### 2.6. Docking Studies of ***4s*** into SMO Receptor

**Figure 6:**
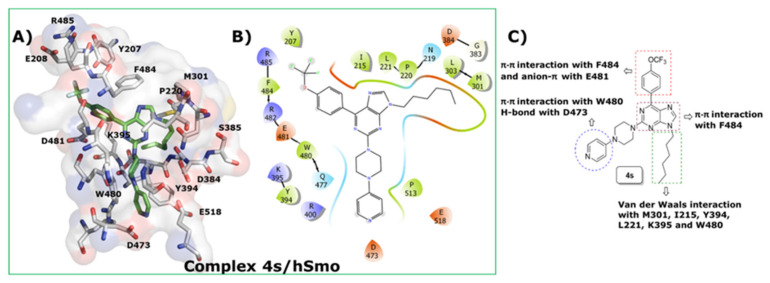
Predicted binding mode of **4s** (green) within the α-heptahelical bundle of the hSMO receptor (PDB ID: 4QIN). (**A**) 3D representation of hSMO in complex with **4s**; residues (less than 5.0 Å) are shown in white stick representation. (**B**) 2D representation of **4s** in a complex with hSMO. (**C**) Main interactions in the **4s**-hSMO complex. Areas of the molecular structure of **4s** which are important for its interaction with SMO are marked with dotted lines.

In order to identify a potential binding pocket and the most stable conformation of the **4s** antagonist interacting with SMO, docking studies were performed. Figure 6A,B shows 3D and 2D descriptions of the proposed molecular interactions of **4s**. 

According to molecular modelling studies, compound **4s** fitted in the same cavity of SMO where other agonist/antagonists have been described to lodge, supporting our structural results [52]. **4s** fits within the SMO receptor heptahelical bundle and established molecular interactions with key residues such as M301, I215, Y394, L221, L303, K395 and W480 (Figure 6). Specifically, the binding cavity provides a highly electron-rich environment (red colour in Supplementary Materials, Figure S4A) which allows π-stacking interactions with the central core of the **4s** purine scaffold.

An H-bond with D473 was also observed when pyridine nitrogen of **4s** was accommodated inside of SMO cavity. Interestingly, based on our experimental data, **4s** was active against the clinically relevant Smo-D473H mutant, indicating that the H-bond generated with D473 contributes at the stabilisation of the ligands into the cavity but is not decisive. In addition, F484 established an extra π–π interaction with the pyridine ring and with the aromatic centre of the trifluoromethylphenyl group. Notably, the amino acid residues described above have been identified in X-ray diffraction studies of the crystal structure of SMO complexed with different ligands (4QIN, 4O9R, 4JKV and 4N4W). Thus, to seek additional support for proposing the site where **4s** binds, we compared this with the location of vismodegib when complexed with SMO. Our findings showed that **4s** and vismodegib attached inside the SMO receptor establishing similar contacts in the heptahelical bundle (Supplementary Materials, Figure S4B). Overall, Figure 6C summarises the main interactions supporting the formation of a complex between **4s** and SMO.

### 2.7. Effect of ***4s*** on Tumour Growth and Metastasis In Vivo

The incidence of aggressive cancers such as melanoma is increasing. These tumours derived from the melanocyte lineage remain incurable after metastasis. Melanomas require HH-GLI signalling [53] regulated by interactions between GLI1 and the Ras-MEK/AKT pathways [54]. Due to this, other novel and potent SMO inhibitors have been recently described [55].

With this evidence and because the induction of apoptosis in tumour cells is an important oncological strategy, we explored the effects of **4s** in vivo. In vitro studies showed that **4s** promotes cell growth arrest and apoptosis. Thus, we evaluated the ability of **4s** to inhibit tumour growth of melanoma B16F10 cells in vivo in a syngeneic mouse model. Given that the solubility of compound **4s** in water is poor, we devised a solubilisation strategy using components approved by the FDA (the oil Miglyol and the natural surfactant Epikuron), to overcome this limitation and increase the cellular absorption benefiting in doing so from our previously acquired know-how [56,57]. We have developed an oil-in-water (O/W) nanoemulsion that has shown safety and effectiveness in both in vitro and in vivo studies when loaded with lipophilic anticancer active molecules [58,59,60,61]. We measured the capacity of this nanoemulsion loaded with **4s** (**4s**-NEM) to inhibit tumour relapse and metastasis following surgery. The selected dose of **4s** encapsulated in the bulk nanoemulsion was 1.5 mg (Supplementary Materials, Figures S5 and S6). The size and zeta potential of the nanoemulsion obtained were ~190 nm and ~−43 mV, respectively, which is in agreement with other work using this formulation and encapsulating lipophilic drugs [56,58,62]. 

**Figure 7:**
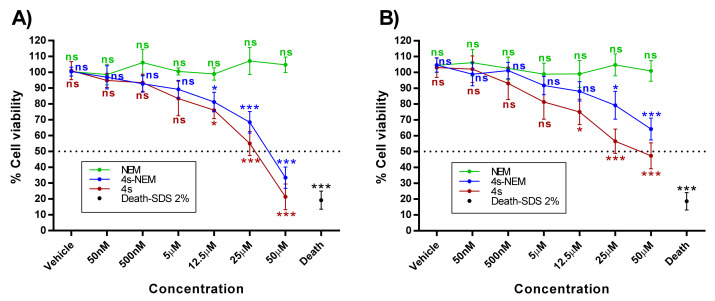
Effect of **4s**-NEM on the viability of cancer (B16F10) and non-cancer (HEK293) cells in vitro. Cells were treated with **4s**-NEM at different concentrations for 24 h and their viability was evaluated using the MTS assay; SDS, the vehicle for NEM, and **4s** free in the culture medium were used as controls. The IC_50_ was determined in each case. (**A**) Effect of **4s**-NEM on B16F10 cells. Six independent experiments were performed in triplicate. Results are shown as the mean ± SEM. (**B**) Effect of **4s**-NEM on HEK293 cells. Results are shown as the mean ± SEM of three separate experiments. (***) *p* < 0.001, (*) *p* < 0.05 vs. control, ns = non-significant. Data analysis was performed via a 2-way ANOVA Tukey’s multiple comparisons test.

**Figure 8:**
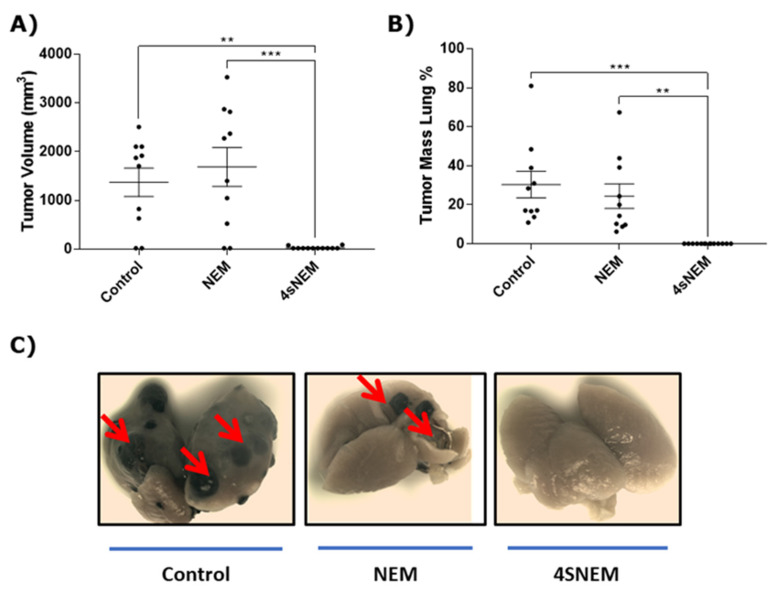
Efficacy of the **4s** compound delivered in an oil-in-water (O/W) nanoemulsion (**4s**-NEM) in a preclinical mouse model. Recurrent tumour growth at the initial site (**A**) and lung metastases (**B**) were evaluated 21 d post-surgery. Mice were injected with B16F10 cells subcutaneously in their flanks. The resulting tumours were surgically removed, and the wounded zone was either not treated (control) or treated with NEM or with **4s**-NEM. Mice were sacrificed 21 d later to evaluate the efficacy of the treatments. Results are presented as the mean ± SD of *n* = 10–12 mice. (***) *p* < 0.001, (**) *p* < 0.01 vs. control. Data analysis was performed via a non-parametric Mann–Whitney test. (**C**) Representative images of lung metastases in control mice (control and NEM) and of animals treated with **4s**-NEM. Note the complete absence of lung metastases in animals treated with **4s**-NEM compared to controls (red arrows).

First, to evaluate the cytotoxicity of **4s** encapsulated in the nanoemulsion, we performed an MTS assay in B16F10 cells (a murine melanoma cell line) and HEK293 cells as control. B16F10 cells were chosen because they express HH pathway genes, such as *Gli1*, and because they are highly resistant, proliferative and metastatic cells [63]. In addition, this cell line is suitable for in vivo studies of tumour formation after subcutaneous injection in syngeneic C57BL/6 mice with a functional immune system, as we have previously described [64,65] and melanomas require HH-GLI signalling [54]. As shown in Figure 7, the B16F10 cell line was sensitive to the effect of **4s**-NEM. It was observed that the IC_50_ of **4s**-NEM at 1.5 µM was comparable to **4s** dissolved in DMSO (29.2 µM for **4s**-NEM vs. 27.5 µM for **4s** in DMSO). However, DMSO is toxic and unable to provide a formulation to be tested with therapeutic purposes [66,67]. Importantly, the formulation **4s**-NEM did not induce a significant decrease in cell viability in HEK293 cells, indicating that the effect of **4s** is specific for tumour cells. These data are in line with our previous in vitro studies. As shown in Figure 7, the NEM vehicle did not affect cell viability suggesting that the nanoemulsion is safe [56]. Studies with C57BL/6 mice showed that a single dose of **4s**-NEM, applied topically on the wounded area following surgical excision of the primary tumour as previously described [56,64], was enough to prevent tumour regrowth (Figure 8A) and spontaneous pulmonary metastasis (Figure 8B,C).

Thus, in a syngeneic mouse melanoma model, **4s** blocked tumour xenograft growth. These results demonstrate that **4s** exerts is a potent suppressor of cancer cell growth, both in vitro and in vivo.

## 3. Discussion

By incorporating substitutions to the purine scaffold, we designed a number of purine derivatives able to interact preferentially with the SMO receptor in the perspective of targeting the HH signalling pathway. From the chemical point of view, although it was not possible to establish a structure–activity relationship because the most compounds were not very active. However, the cytotoxic efficacy shown by compounds **4r** and **4s** might suggest that C-2 substitutions on the purine ring with a cyclic amine and an aromatic ring containing a nitrogen, such as the 1-methyl-4-(pyridin-4-yl)piperazine group, may be essential to obtain more potent compounds than alicyclic derivatives. 

Using in vitro and in vivo assays, we identified one compound (**4s**) that showed cytotoxic effects in various cancer cell lines, with IC_50_ values ranging from 1.3 to 15 µM. **4s** shows an anti-proliferative activity, induces cell cycle arrest and apoptosis, and inhibits colony formation of multiple cancer cell types Additionally, we found that **4s** promoted the inhibition of genes targeted by the HH signalling pathway, and acts as a SMO antagonist in vitro. 

The anti-proliferative effect exhibited by **4s** could be explained my mechanisms of targeting and SMO destabilisation. The significant increment in G_0_/G_1_ in **4s**-treated HT29 cells confirmed that the anticancer effect of this compound is through the arrest of cell cycle in G_0_/G_1_-phase and subsequent activation of apoptosis. It is known that the cell cycle transition through different phases is coordinated by CDKs and their activating partners, the cyclins [68,69]. Although it was not determined in this study, it is probable that **4s**-induced cell cycle arrest is likely mediated via an induction of p21WAF1 and p27kip1, with a concomitant inhibition of CDK2 and CDK4, along with cyclins D1 and E1. Previous studies suggested additional effects of HH signalling on metastasis and cancer stem cells besides its function in promoting cancer cell proliferation [70,71,72]. Upregulated HH signalling was found to facilitate metastasis and to be associated with poor prognosis in various types of cancer, possibly due to its role in regulating stemness of CSCs. In our study, **4s** affects colony formation more than vismodegib, confirming its cytotoxic effect on colon cancer cells. Cell proliferation was inhibited, while apoptosis was increased by **4s** probably affecting the expression of B cell lymphoma 2 (Bcl 2), an anti-apoptotic downstream target of HH signalling. There are many examples throughout the literature about mRNA and protein levels of Bcl-2 being downregulated while cell apoptosis is augmented. Simultaneously, the expression of Bax pro-apoptotic protein should increase in response to apoptotic stimuli. Vismodegib is known to inhibit the replication of colon cancer cells and trigger apoptosis through downregulating Bcl-2 although its effect depends on the cell type [73,74,75]. Vismodegib induces apoptosis at concentrations greater than 100 µM in pancreatic cancer cells [76] with less efficiency than **4s**. In pancreatic cancer cell lines and pancreatic cancer stem cells (CSCs), vismodegib inhibits cell viability and induces apoptosis increasing Fas expression and decreasing expression of PDGFRα. Vismodegib also suppresses cell viability, GLI-DNA binding and transcriptional activities, and induces apoptosis through caspase-3 activation and PARP cleavage [49]. The possible involvement of caspases in the effects exhibited by **4s** in vitro remains to be elucidated. Fas expression is low in human and murine basal cell carcinomas but is upregulated in the presence of the SMO antagonist, cyclopamine, both in vitro as in vivo. This parallels an elevated rate of apoptosis. Conversely, expression of activated SMO inhibits Fas expression. Fas/Fas ligand interactions are necessary for cyclopamine-mediated apoptosis in these cells, a process involving caspase-8 activation [77]. How **4s** is related to Fas/Fas ligand expression is an association that is currently under study. Similar results are observed when a small molecule inhibitor of GLI1 and GLI2, GANT61, was used to block HH signalling in human colon carcinoma cell lines that express HH signalling components [44].

In vivo studies results demonstrated that **4s** exerted a potent suppressor of cancer cell growth, both in vitro and in vivo. The administration of a single dose of **4s**, presented as a nanoemulsion, was sufficient to completely prevent tumour regrowth and spontaneous lung metastasis. The ability of **4s** to induce apoptosis likely potentiates its ability to reduce tumour growth. Thus, **4s** seems to function both as a potent HH pathway inhibitor and an effective inducer of apoptosis, with significant potential for the development of new cancer therapies. 

It remains to be demonstrated whether the effects observed by **4s** in vivo are comparable to those exhibited by nanoemulsified vismodegib under the same experimental conditions. These findings support the use of SMO inhibitors for melanoma therapy, as well as for other types of cancers with an active HH signalling pathway. It would be interesting to compare the preclinical antitumour efficacy and the pharmacokinetic/pharmacodynamic profile of **4s** to those of vismodegib. Recently, other new inhibitors have been assayed in melanoma models [78]. Inhibitors which use the acylthiourea or acylguanidine scaffolds elicit an important reduction in growth and regeneration of melanoma cells [78]. Lastly, inhibition of HH-GLI pathway by the SMO inhibitor NVP-LDE225 is associated to the inhibition of cell growth and induction of apoptosis in human melanoma cell lines. Further observations demonstrated that NVP-LDE225 also reduced cell proliferation and induced tumour growth arrest in vitro and in vivo, when compared to cyclopamine [79]. 

## 4. Conclusions

We identified compound **4s** as a SMO antagonist that induced apoptosis and reduced the expression of several HH signalling pathway target genes involved in tumour growth. Furthermore, **4s** was more efficient than vismodegib in vitro in inducing apoptosis in human cancer cells. Taken together, these results establish compound **4s** as a novel SMO antagonist that curtails HH-dependent tumour growth, both in vitro and in vivo.

## 5. Materials and Methods

### 5.1. Chemistry

#### 5.1.1. Materials

Melting points were determined on a Kofler Thermogerate apparatus and were uncorrected. Infrared spectra were recorded on a JASCO FT/IR-400 spectrophotometer. Nuclear magnetic resonance spectra were recorded, unless otherwise specified, on a Bruker AM-400 instrument using deuterated chloroform or dimethylsulfoxide solutions containing tetramethylsilane as an internal standard. ESI/MS experiment was carried out on an UHPLC Eksigent1 coupled with MS detector ABSciex1, Triple Quad 4500 model equipment. HRMS-ESI-MS experiments were performed using a Thermo Scientific Exactive Plus Orbitrap spectrometer with a constant nebuliser temperature of 250 °C. The experiments were carried out in positive or negative ion mode, with a scan range of *m*/*z* 300.00–1510.40 and a resolution of 140,000. The samples were infused directly into the ESI source via a syringe pump at flow rates of 5 µL·min^−1^ through the instrument’s injection valve. Thin layer chromatography (TLC) was performed using Merck GF-254 type 60 silica gel. Column chromatography was carried out using Merck type 9385 silica gel. The purity of the compounds was determined by TLC and high-resolution mass spectrometry (HRMS) and for **4s** by HLPC (Figure S8).

#### 5.1.2. General Procedure of the Synthesis for Compounds **2a**–**2c** and **2a′**–**2c′**

A mixture of 6-chloro-2-fluoropurine **1** (1.0 mmol), the respective alkyl halide (1.5 mmol), and potassium carbonate (3.0 mmol) in DMF (5 mL) was stirred for 12 h, then the mixture was filtered, washed with EtOAc and evaporated under vacuum. The regioisomeric products were separated by flash chromatography on silica gel eluting with EtOAc/CH_2_Cl_2_ (2:3).

9-Butyl-6-chloro-2-fluoro-9H-purine (**2a**) White solid, yield 36%, m.p. 76–78 °C. ^1^H NMR (200 MHz, CDCl_3_) δ 8.09 (s, 1H), 4.25 (t, *J* = 7.2 Hz, 2H), 1.99–1.83 (m, 2H), 1.49–1.24 (m, 2H), 0.98 (t, *J* = 7.3 Hz, 3H). ^13^C NMR (50 MHz, CDCl_3_) δ 159.44–155.08 (d, *J*_CF_ = 219.6 Hz), 153.87–153.50 (d, *J*_CF_ = 18.3 Hz), 152.82–152.47 (d, *J*_CF_ = 17.6 Hz), 145.76–145.69 (d, *J*_CF_ = 3.1 Hz), 130.32–130.22 (d, *J*_CF_ = 5.0 Hz), 44.38, 31.65, 19.78, 13.38. ^19^F NMR (188 MHz, CDCl_3_) δ −49.69. IR (KBr, cm^−1^): 2959, 1605, 1579, 1511, 1408, 1332, 920. ESI/MS calcd. for (C_9_H_10_ClFN_4_ [M + H]^+^): 229.1. Found: 229.1.

7-Butyl-6-chloro-2-fluoro-7H-purine (**2a′**) Yellow solid, yield 13%, m.p. 61–64 °C. ^1^H NMR (400 MHz, CDCl_3_) δ 8.21 (d, *J* = 4.9 Hz, 1H), 4.42 (t, *J* = 7.3 Hz, 2H), 1.87 (dt, *J* = 15.1, 7.5 Hz, 2H), 1.41–1.32 (m, 2H), 0.94 (t, *J* = 7.4 Hz, 3H). ^13^C NMR (101 MHz, CDCl_3_) δ 164.35–164.17 (d, *J*_CF_ = 17.3 Hz), 158.55–156.38 (d, *J*_CF_ = 217.5 Hz), 150.74, 144.62–144.44 (d, *J*_CF_ = 18.3 Hz), 121.14–121.09 (d, *J*_CF_ = 5.4 Hz) 47.45, 33.46, 19.59, 13.44. ^19^F NMR (376 MHz, CDCl_3_) δ −50.30. IR (KBr, cm^−1^): 2961, 1608, 1485, 1396, 1036. ESI/MS calcd. for (C_9_H_10_ClFN_4_ [M + H]^+^): 229.1. Found: 229.0.

6-Chloro-2-fluoro-9-pentyl-9H-purine (**2b**) Yellow solid, yield 47%, m.p. 92–93 °C. ^1^H NMR (400 MHz, CDCl_3_) δ 8.05 (s, 1H), 4.19 (t, *J* = 7.3 Hz, 2H), 1.93–1.83 (m, 2H), 1.39–1.22 (m, 4H), 0.85 (t, *J* = 6.9 Hz, 3H). ^13^C NMR (101 MHz, CDCl_3_) δ 158.35–156.17 (d, *J*_CF_ = 219.6 Hz), 153.76–153.59 (d, *J*_CF_ = 17.0 Hz), 152.71–152.54 (d, *J*_CF_ = 17.6 Hz), 145.79–145.76 (d, *J*_CF_ = 3.2 Hz), 130.31–130.26 (d, *J*_CF_ = 5.0 Hz), 44.66, 29.37, 28.63, 22.04, 13.79. ^19^F NMR (376 MHz, CDCl_3_) δ −49.71. IR (KBr, cm^−1^): 2958, 1599, 1578, 1510, 1406, 1338, 920. ESI/MS calcd. for (C_10_H_12_ClFN_4_ [M + H]^+^): 243.1. Found: 243.1.

6-Chloro-2-fluoro-7-pentyl-7H-purine (**2b′**) White solid, yield 12%, m.p. 42–43 °C. ^1^H NMR (400 MHz, CDCl_3_) δ 8.21 (s, 1H), 4.41 (t, *J* = 7.4 Hz, 2H), 1.95–1.84 (m, 2H), 1.39–1.24 (m, 4H), 0.87 (t, *J* = 6.8 Hz, 3H). ^13^C NMR (101 MHz, CDCl_3_) δ 164.37–164.20 (d, *J*_CF_ = 17.2 Hz), 158.57–156.41 (d, *J*_CF_ = 217.5 Hz), 150.70, 144.61–144.43 (d, *J*_CF_ = 18.2 Hz), 121.13–121.07 (d, *J*_CF_ = 6.0 Hz), 47.71, 31.22, 28.45, 22.08, 13.81. ^19^F NMR (376 MHz, CDCl_3_) δ −50.23. IR (KBr, cm^−1^): 2953, 1610, 1553, 1490, 1363, 1084, 939. ESI/MS calcd. for (C_10_H_12_ClFN_4_ [M + H]^+^): 243.1. Found: 243.0.

6-Chloro-2-fluoro-9-hexyl-9H-purine (**2c**) Yellow solid, yield 60%, m.p. 96–99 °C. ^1^H NMR (400 MHz, CDCl_3_) δ 8.03 (s, 1H), 4.10 (t, *J* = 7.2 Hz, 2H), 1.81–1.71 (m, 2H), 1.13 (m, 6H), 0.64 (t, *J* = 6.7 Hz, 3H). ^13^C NMR (101 MHz, CDCl_3_) δ158.04–155.86 (d, *J*_CF_ = 219.7 Hz), 153.69–153.52 (d, *J*_CF_ = 16.9 Hz), 152.14–151.96 (d, *J*_CF_ = 17.6 Hz), 146.08–146.05 (d, *J*_CF_ = 3.1 Hz), 130.12–130.07 (d, *J*_CF_ = 4.8 Hz), 44.57, 30.92, 29.47, 26.06, 22.22, 13.70. ^19^F NMR (376 MHz, CDCl_3_) δ −49.73. IR (KBr, cm^−1^): 2859, 1577, 1510, 1338, 1212, 921. ESI/MS calcd. for (C_11_H_14_ClFN_4_ [M + H]^+^): 257.1. Found: 257.1.

6-Chloro-2-fluoro-7-hexyl-7H-purine (**2c′**) Yellow solid, yield 16%, m.p. 83–85 °C. ^1^H NMR (400 MHz, CDCl_3_) δ 8.20 (s, 1H), 4.41 (t, *J* = 7.4 Hz, 2H), 1.99–1.62 (m, 2H), 1.39–1.16 (m, 6H), 0.84 (t, *J* = 5.7 Hz, 3H). ^13^C NMR (101 MHz, CDCl_3_) δ 164.38–164.21 (d, *J*_CF_ = 17.1 Hz), 158.59–156.43 (d, *J*_CF_ = 217.6 Hz), 150.66, 145.83–145.80 (d, *J*_CF_ = 3.1 Hz), 144.61–144.43 (d, *J*_CF_ = 18.3 Hz), 47.72, 31.48, 31.09, 26.03, 22.40, 13.87. ^19^F NMR (376 MHz, CDCl_3_) δ −50.18. IR (KBr, cm^−1^): 2931, 1607, 1578, 1549, 1485, 1333, 1037. ESI/MS calcd. for (C_11_H_14_ClFN_4_ [M + H]^+^): 257.1. Found: 257.1.

#### 5.1.3. General Procedure for the Synthesis of Compounds **3a**–**3c**

A stirred solution of **2a**–**2c** derivatives (1.0 mmol), (4-(trifluoromethoxy)phenyl)boronic acid (1.0 mmol), 2 M aqueous solution of potassium carbonate (1 mL per mmol of **3a**–**3c**), and palladium(II)bis(triphenylphosphine) dichloride (0.1 mmol) in dioxane (5 mL) was heated to reflux during 3 h. Then, the solution was cooled to room temperature and extracted with ethyl acetate. The organic layer was dried with anhydrous sodium sulphate, filtered and concentrated under vacuum. The crude product was purified on a silica gel column using dichloromethane as eluent.

9-Butyl-2-fluoro-6-(4-(trifluoromethoxy)phenyl)-9H-purine (**3a**) White solid, yield 41%, m.p. 67–69 °C. ^1^H NMR (400 MHz, CDCl_3_) δ 8.73 (d, *J* = 8.8 Hz, 2H), 7.92 (s, 1H), 7.22 (d, *J* = 8.5 Hz, 2H), 4.10 (t, *J* = 7.3 Hz, 2H), 1.81–1.71 (m, 2H), 1.24 (m, 2H), 0.82 (t, *J* = 7.4 Hz, 3H).^13^C NMR (101 MHz, CDCl_3_) δ 159.80–157.68 (d, *J*_CF_ = 212.9 Hz), 155.63–155.48 (d, *J*_CF_ = 15.3 Hz), 155.16–155.01 (d, *J*_CF_ = 16.7 Hz), 151.71, 145.12–145.09 (d, *J*_CF_ = 3.0 Hz), 133.03, 131.79 (2C), 129.68–129.63 (d, *J*_CF_ = 4.3 Hz), 121.70–119.14 (d, *J*_CF_ = 4.3 Hz), 120.66 (2C), 43.94, 31.76, 19.88, 13.47. ^19^F NMR (376 MHz, CDCl_3_) δ −50.65, −57.56 (3F). IR (KBr, cm^−1^): 2964, 1597, 1578, 1517, 1359, 1250, 1219, 1117. ESI/MS calcd. for (C_16_H_14_F_4_N_4_O [M + H]^+^): 355.1. Found: 355.2.

2-Fluoro-9-pentyl-6-(4-(trifluoromethoxy)phenyl)-9H-purine (**3b**) White solid, yield 57%, m.p. 72–73 °C. ^1^H NMR (400 MHz, CDCl_3_) δ 8.73 (d, *J* = 8.8 Hz, 2H), 7.93 (s, 1H), 7.22 (d, *J* = 8.5 Hz, 2H), 4.09 (t, *J* = 7.3 Hz, 2H), 1.82–1.74 (m, 2H), 1.26–1.15 (m, 4H), 0.74 (t, *J* = 6.9 Hz, 3H).^13^C NMR (101 MHz, CDCl_3_) δ 159.79–157.67 (d, *J*_CF_ = 213.0 Hz), 155.61–155.45 (d, *J*_CF_ = 14.7 Hz), 155.15–154.98 (d, *J*_CF_ = 15.0 Hz), 151.72, 145.11, 133.02, 131.79 (2C), 129.67, 121.70–119.13 (d, *J*_CF_ = 4.3 Hz), 120.65 (2C), 44.19, 29.45, 28.72, 22.10, 13.82. ^19^F NMR (376 MHz, CDCl_3_) δ −50.67, −57.57 (3F). IR (KBr, cm^−1^): 2964, 1596, 1576, 1514, 1360, 1293, 1095. ESI/MS calcd. for (C_17_H_16_F_4_N_4_O [M + H]^+^): 369.1. Found: 369.2.

2-Fluoro-9-hexyl-6-(4-(trifluoromethoxy)phenyl)-9H-purine (**3c**) White solid, yield 51%, m.p. 80–82 °C. ^1^H NMR (400 MHz, CDCl_3_) δ 8.73 (d, *J* = 8.8 Hz, 2H), 7.92 (s, 1H), 7.22 (d, *J* = 8.5 Hz, 2H), 4.09 (t, *J* = 7.3 Hz, 2H), 1.81–1.72 (m, 2H), 1.26–1.07 (m, 6H), 0.72 (t, *J* = 6.8 Hz, 3H). ^13^C NMR (101 MHz, CDCl_3_) δ 159.79–157.68 (d, *J*_CF_ = 213.0 Hz), 155.62–155.47 (d, *J*_CF_ = 15.4 Hz), 155.15–154.99 (d, *J*_CF_ = 16.8 Hz), 151.71, 145.13–145.10 (d, *J*_CF_ = 3.0 Hz), 133.04 (2C), 129.64, 129.67–129.63 (d, *J*_CF_ = 4,2 Hz), 121.70–119.13 (d, *J*_CF_ = 259.6 Hz), 120.66 (2C), 44.21, 31.14, 29.73, 26.30, 22.43, 13.91. ^19^F NMR (376 MHz, CDCl_3_) δ −50.66, −57.56 (3F). IR (KBr, cm^−1^): 2921, 1596, 1573, 1515, 1371, 1305, 1278, 1155. ESI/MS calcd. for (C_18_H_18_F_4_N_4_O [M + H]^+^): 383.1. Found: 383.2.

#### 5.1.4. General Procedure for the Synthesis of Compounds **4a**–**4s**

Compounds **3a**–**3c** (1.0 mmol), the respective amine (3.0 mmol) and DIPEA (3.0 mmol) were dissolved in *n*-BuOH (5 mL) and the mixture was heated to 110 °C for 12 h. Then the reaction mixture was cooled and concentrated under vacuum. The crude products were purified on silica gel columns using chloroform as mobile phase.

9-Butyl-N-cyclopentyl-6-(4-(trifluoromethoxy)phenyl)-9H-purin-2-amine (**4a**) Yellow solid, yield 89%, m.p. 93–95 °C. ^1^H NMR (400 MHz, CDCl_3_) δ 8.76 (d, *J* = 8.8 Hz, 2H), 7.73 (s, 1H), 7.35 (d, *J* = 8.3 Hz, 2H), 5.19 (d, *J* = 6.7 Hz, 1H), 4.45–4.33 (m, 1H), 4.11 (t, *J* = 7.1 Hz, 2H), 2.11 (m, 2H), 1.92–1.82 (m, 2H), 1.82–1.60 (m, 4H), 1.55 (m, 2H), 1.36 (m, 2H), 0.96 (t, *J* = 7.4 Hz, 3H). ^13^C NMR (101 MHz, CDCl_3_) δ 159.17, 154.70, 153.59, 150.71–150.70 (d, *J*_CF_ = 1.7 Hz), 141.22, 135.00, 131.12 (2C), 124.84, 121.77–119.21 (d, *J*_CF_ = 258.6 Hz), 120.52 (2C), 53.51, 42.95, 33.36 (2C), 31.74, 23.85 (2C), 19.83, 13.49. ^19^F NMR (188 MHz, CDCl_3_) δ −57.61 (3F). IR (KBr, cm^−1^): 3387, 2965, 1603, 1503, 1270, 1221, 1165. HRMS calcd. for (C_21_H_24_F_3_N_5_O [M + H]^+^): 420.2013. Found: 420.1996. 

N-cyclopentyl-9-pentyl-6-(4-(trifluoromethoxy)phenyl)-9H-purin-2-amine (**4b**) Yellow solid, yield 87%, m.p. 62–63 °C. ^1^H NMR (400 MHz, CDCl_3_) δ 8.77 (d, *J* = 8.8 Hz, 2H), 7.73 (s, 1H), 7.35 (d, *J* = 8.4 Hz, 2H), 5.19 (d, *J* = 6.7 Hz, 1H), 4.44–4.32 (m, 1H), 4.10 (t, *J* = 7.1 Hz, 2H), 2.12 (td, *J* = 12.1, 6.5 Hz, 2H), 1.93–1.84 (m, 2H), 1.82–1.60 (m, 4H), 1.60–1.48 (m, 2H), 1.42–1.27 (m, 4H), 0.90 (t, *J* = 7.0 Hz, 3H). ^13^C NMR (101 MHz, CDCl_3_) δ 159.17, 154.69, 153.58, 150.72–150.70 (d, *J*_CF_ = 1.7 Hz), 141.23, 135.00, 131.11 (2C), 124.86, 121.77–119.21 (d, *J*_CF_ = 258.6 Hz), 120.52 (2C), 53.51, 43.26, 33.36 (2C), 29.36, 28.77, 23.85 (2C), 22.12, 13.84. ^19^F NMR (188 MHz, CDCl_3_) δ −57.61 (3F). IR (KBr, cm^−1^): 3275, 2967, 1604, 1505, 1275, 1219, 1163. HRMS calcd. for (C_22_H_26_F_3_N_5_O [M + H]^+^): 434.2169. Found: 434.2154.

N-cyclopentyl-9-hexyl-6-(4-(trifluoromethoxy)phenyl)-9H-purin-2-amine (**4c**) Yellow solid, yield 97%, m.p. 58–60 °C. ^1^H NMR (400 MHz, CDCl_3_) δ 8.64 (d, *J* = 8.7 Hz, 2H), 7.61 (s, 1H), 7.22 (d, *J* = 8.5 Hz, 2H), 5.07 (d, *J* = 6.6 Hz, 1H), 4.32–4.21 (m, 1H), 3.97 (t, *J* = 7.1 Hz, 2H), 2.03–1.96 (m, 2H), 1.80–1.71 (m, 2H), 1.70–1.50 (m, 4H), 1.45–1.39 (m, 2H), 1.28–1.12 (m, 6H), 0.75 (t, *J* = 6.8 Hz, 3H). ^13^C NMR (101 MHz, CDCl_3_) δ 159.16, 154.69, 153.56, 150.72–150.70 (d, *J*_CF_ = 1.6 Hz), 141.23, 135.00, 131.12 (2C), 124.85, 121.77–119.21 (d, *J*_CF_ = 257.6 Hz), 120.51 (2C), 53.51, 43.26, 33.36 (2C), 31.19, 29.63, 26.30, 23.85 (2C), 22.44, 13.92. ^19^F NMR (188 MHz, CDCl_3_) δ −57.61 (3F). IR (KBr, cm^−1^): 3277, 2958, 1606, 1506, 1257, 1221, 1205, 1163. HRMS calcd. for (C_23_H_28_F_3_N_5_O [M + H]^+^): 448.2326. Found: 448.2313.

9-Butyl-N-cyclohexyl-6-(4-(trifluoromethoxy)phenyl)-9H-purin-2-amine (**4d**) White solid, yield 90%, m.p. 92–94 °C. ^1^H NMR (400 MHz, CDCl_3_) δ 8.71 (d, *J* = 8.9 Hz, 2H), 7.70 (s, 1H), 7.31 (d, *J* = 8.2 Hz, 2H), 5.02 (d, *J* = 7.4 Hz, 1H), 4.09 (s, 2H), 3.99–3.85 (m, 1H), 2.10 (m, 2H), 1.89–1.81 (m, 2H), 1.80–1.73 (m, 2H), 1.68–1.60 (m, 1H), 1.43 (dd, *J* = 16.4, 3.3 Hz, 2H), 1.36 (dd, *J* = 15.0, 7.4 Hz, 2H), 1.32–1.20 (m, 3H), 0.95 (t, *J* = 7.4 Hz, 3H). ^13^C NMR (101 MHz, CDCl_3_) δ 158.78, 154.77, 153.71, 150.73 (d, *J*_CF_ = 1.7 Hz), 141.20, 134.98, 131.09 (2C) 124.85, 121.77–119.21 (d, *J*_CF_ = 258.6 Hz), 120.56 (2C), 50.29, 42.94, 33.23 (2C), 31.77, 25.91, 25.00 (2C), 19.85, 13.52. ^19^F NMR (188 MHz, CDCl_3_) δ −57.60 (3F). IR (KBr, cm^−1^): 3445, 2932, 1611, 1529, 1271, 1209, 1158. HRMS calcd. for (C_22_H_26_F_3_N_5_O [M + H]^+^): 434.2169. Found: 448.2313. 

N-cyclohexyl-9-pentyl-6-(4-(trifluoromethoxy)phenyl)-9H-purin-2-amine (**4e**) White solid, yield 77%, m.p. 108–110 °C. ^1^H NMR (400 MHz, CDCl_3_) δ 8.76 (d, *J* = 8.8 Hz, 2H), 7.73 (s, 1H), 7.35 (d, *J* = 8.4 Hz, 2H), 5.10 (d, *J* = 7.7 Hz, 1H), 4.09 (t, *J* = 7.2 Hz, 2H), 4.01–3.87 (m, 1H), 2.19–2.08 (m, 2H), 1.95–1.84 (m, 2H), 1.84–1.74 (m, 2H), 1.53–1.19 (m, 10H), 0.90 (t, *J* = 7.0 Hz, 3H). ^13^C NMR (101 MHz, CDCl_3_) δ 158.78, 154.75, 153.66, 150.72–150.70 (d, *J*_CF_ = 1.7 Hz), 141.21, 135.00, 131.10 (2C), 124.83, 121.77–119.21 (d, *J*_CF_ = 258.6 Hz), 120.53 (2C), 50.28, 43.23, 33.20 (2C), 29.38, 28.76, 25.89, 24.99 (2C), 22.10, 13.84. ^19^F NMR (188 MHz, CDCl_3_) δ −57.61 (3F). IR (KBr, cm^−1^): 3440, 2932, 1611, 1529, 1270, 1209, 1160. HRMS calcd. for (C_23_H_28_F_3_N_5_O [M + H]^+^): 448.2326. Found: 448.2308.

N-cyclohexyl-9-hexyl-6-(4-(trifluoromethoxy)phenyl)-9H-purin-2-amine (**4f**) Yellow solid, yield 96%, m.p. 84–86 °C. ^1^H NMR (400 MHz, CDCl_3_) δ 8.63 (d, *J* = 8.7 Hz, 2H), 7.61 (s, 1H), 7.22 (d, *J* = 8.4 Hz, 2H), 5.03 (s, 1H), 3.97 (t, *J* = 7.1 Hz, 2H), 3.87–3.78 (m, 1H), 2.00 (d, *J* = 9.4 Hz, 2H), 1.81–1.61 (m, 2H), 1.40–1.07 (m, 12H), 0.81–0.68 (m, 5H). ^13^C NMR (101 MHz, CDCl_3_) δ 158.68, 154.79, 153.56, 150.76–150.74 (d, *J*_CF_ = 1.6 Hz), 141.30, 134.85, 131.13 (2C), 124.80, 121.77–119.21 (d, *J*_CF_ = 257.6 Hz), 120.54 (2C), 50.28, 43.27, 33.18 (2C), 31.19, 29.64, 26.31, 25.89, 24.98 (2C), 22.44, 13.94. ^19^F NMR (188 MHz, CDCl_3_) δ −57.61 (3F). IR (KBr, cm^−1^): 3442, 2921, 1610, 1509, 1278, 1188, 1158. HRMS calcd. for (C_24_H_30_F_3_N_5_O [M + H]^+^): 462.2482. Found: 462.2467.

9-Butyl-N-(cyclohexylmethyl)-6-(4-(trifluoromethoxy)phenyl)-9H-purin-2-amine (**4g**) White solid, yield 94%, m.p. 88–89 °C. ^1^H NMR (400 MHz, CDCl_3_) δ 8.67 (d, *J* = 8.8 Hz, 2H), 7.64 (s, 1H), 7.26 (d, *J* = 8.4 Hz, 2H), 5.19 (s, 1H), 4.01 (t, *J* = 7.1 Hz, 2H), 3.29 (t, *J* = 6.3 Hz, 2H), 1.81–173 (m, 4H), 1.70–1.61 (m, 2H), 1.61–1.48 (m, 2H), 1.35–1.23 (m, 2H), 1.23–1.06 (m, 3H), 0.95 (m, 2H), 0.88 (t, *J* = 7.4 Hz, 3H). ^13^C NMR (101 MHz, CDCl_3_) δ 159.70, 154.71, 153.58, 150.70 (d, *J*_CF_ = 1.7 Hz), 141.19, 135.00, 131.12 (2C), 124.83, 121.77–119.21 (d, *J*_CF_ = 258.6 Hz), 120.52 (2C), 53.40, 48.35, 42.94, 38.22, 31.74, 31.12 (2C), 26.55, 26.01, 19.84, 13.49. ^19^F NMR (188 MHz, CDCl_3_) δ −57.60 (3F). IR (KBr, cm^−1^): 3326, 2926, 1604, 1508, 1294, 1259, 1208. HRMS calcd. for (C_23_H_28_F_3_N_5_O [M + H]^+^): 448.2326. Found: 448.2303.

N-(cyclohexylmethyl)-9-pentyl-6-(4-(trifluoromethoxy)phenyl)-9H-purin-2-amine (**4h**) White solid, yield 82%, m.p. 78–79 °C. ^1^H NMR (400 MHz, CDCl_3_) δ 8.62 (d, *J* = 8.5 Hz, 2H), 7.61 (s, 1H), 7.22 (d, *J* = 8.3 Hz, 2H), 5.10 (s, 1H), 3.98 (t, *J* = 7.1 Hz, 2H), 3.26 (t, *J* = 6.1 Hz, 2H), 1.82–1.69 (m, 4H), 1.68–1.50 (m, 4H), 1.32–1.04 (m, 7H), 0.95–0.87 (m, 2H), 0.78 (t, *J* = 6.8 Hz, 3H). ^13^C NMR (101 MHz, CDCl_3_) δ 159.71, 154.71, 153.64, 150.74–150.72 (d, *J*_CF_ = 1.7 Hz), 141.21, 134.98, 131.12 (2C), 124.87, 121.77–119.21 (d, *J*_CF_ = 258.6 Hz), 120.55 (2C), 48.37, 43.27, 38.26, 31.14 (2C), 29.39, 28.78, 26.57, 26.02 (2C), 22.15, 13.89. ^19^F NMR (188 MHz, CDCl_3_) δ −57.61 (3F). IR (KBr, cm^−1^): 3332, 2926, 1606, 1506, 1258, 1218, 1172. HRMS calcd. for (C_24_H_30_F_3_N_5_O [M + H]^+^): 462.2482. Found: 462.2465.

N-(cyclohexylmethyl)-9-hexyl-6-(4-(trifluoromethoxy)phenyl)-9H-purin-2-amine (**4i**) White solid, yield 93%, m.p. 85–86 °C. ^1^H NMR (400 MHz, CDCl_3_) δ 8.65 (d, *J* = 8.8 Hz, 2H), 7.63 (s, 1H), 7.24 (d, *J* = 8.4 Hz, 2H), 5.12 (s, 1H), 4.00 (t, *J* = 7.2 Hz, 2H), 3.28 (t, *J* = 6.3 Hz, 2H), 1.83–1.53 (m, 7H), 1.31–1.04 (m, 10H), 1.01–0.85 (m, 2H), 0.78 (t, *J* = 6.9 Hz, 3H). ^13^C NMR (101 MHz, CDCl_3_) δ 159.70, 154.70, 153.62, 150.73–150.71 (d, *J*_CF_ = 1.6 Hz), 141.20, 134.99, 131.11 (2C), 124.87, 121.77–119.21 (d, *J*_CF_ = 257.6 Hz), 120.54 (2C), 48.36, 43.29, 38.26, 31.22, 31.13 (2C), 29.65, 26.56 (2C), 26.32, 26.02, 22.46, 13.96. ^19^F NMR (188 MHz, CDCl_3_) δ −57.61 (3F). IR (KBr, cm^−1^): 3320, 2928, 1609, 1507, 1255, 1221, 1165. HRMS calcd. for (C_25_H_32_F_3_N_5_O [M + H]^+^): 476.2639. Found: 476.2622.

9-Pentyl-2-(4-phenylpiperazin-1-yl)-6-(4-(trifluoromethoxy)phenyl)-9H-purine (**4J**) Brown solid, yield 90%, m.p. 119–120 °C. ^1^H NMR (400 MHz, CDCl_3_) δ 9.04 (d, *J* = 8.8 Hz, 2H), 7.99 (s, 1H), 7.66–7.41 (m, 4H), 7.22 (d, *J* = 8.2 Hz, 2H), 7.11 (t, *J* = 7.2 Hz, 1H), 4.47–4.24 (m, 6H), 3.63–3.44 (m, 4H), 2.23–2.00 (m, 2H), 1.70–1.40 (m, 4H), 1.12 (t, *J* = 7.0 Hz, 3H). ^13^C NMR (101 MHz, CDCl_3_) δ 158.70, 154.66, 152.97, 151.46, 150.74–150.73 (d, *J*_CF_ = 1.6 Hz), 141.72, 135.11, 131.15 (2C), 129.15 (2C), 124.65, 121.73–119.17 (d, *J*_CF_ = 257.7 Hz), 120.50 (2C), 120.04, 116.47 (2C), 49.41 (2C), 44.52 (2C), 43.18, 29.32, 28.73, 22.06, 13.85. ^19^F NMR (188 MHz, CDCl_3_) δ −57.62 (3F). IR (KBr, cm^−1^): 2932, 2856, 1531, 1269, 1229, 1160. HRMS calcd. for (C_27_H_29_F_3_N_6_O [M + H]^+^): 511.2435. Found: 511.2415. 

9-Hexyl-2-(4-phenylpiperazin-1-yl)-6-(4-(trifluoromethoxy)phenyl)-9H-purine (**4k**) Yellow solid, yield 81%, m.p. 99–101°C. ^1^H NMR (400 MHz, CDCl_3_) δ 8.69–8.66 (m, 2H), 7.64 (s, 1H), 7.22 (d, *J* = 8.2 Hz, 2H), 7.17 (t, *J* = 8.3 Hz, 2H), 6.90 (d, *J* = 7.8 Hz, 2H), 6.78 (t, *J* = 7.2 Hz, 1H), 4.00 (t, *J* = 7.0 Hz, 6H), 3.23–3.17 (m, 4H), 1.82–1.71 (m, 2H), 1.30–1.14 (m, 6H), 0.75 (t, *J* = 7.0 Hz, 3H). ^13^C NMR (101 MHz, CDCl_3_) δ 158.73, 154.72, 153.07, 150.82 (d, *J*_CF_ = 1.6 Hz), 141.80 (2C), 135.13, 131.21 (2C), 129.27 (2C), 124.74, 124.35, 121.78–119.22 (d, *J*_CF_ = 258.6 Hz), 120.57 (2C), 116.71 (2C), 49.69 (2C), 44.49 (2C), 43.27, 31.19, 29.65, 26.33, 22.49, 13.99. ^19^F NMR (188 MHz, CDCl_3_) δ −57.60 (3F). IR (KBr, cm^−1^): 2954, 1530, 1266, 1228, 1158. HRMS calcd. for (C_28_H_31_F_3_N_6_O [M + H]^+^): Calcd: 525.2591. Found: 525.2581.

9-Pentyl-2-(4-(4-clorophenyl)piperazin-1-yl)-6-(4-(trifluoromethoxy)phenyl)-9H-purine (**4l**) Yellow solid, yield 82%, m.p. 147–149 °C. ^1^H NMR (400 MHz, CDCl_3_) δ 8.73 (d, *J* = 8.9 Hz, 2H), 7.71 (s, 1H), 7.28 (d, *J* = 8.3 Hz, 2H), 7.15 (d, *J* = 8.7 Hz, 2H), 6.83 (d, *J* = 12.1 Hz, 2H), 4.21–3.83 (m, 6H), 3.32–3.07 (m, 4H), 1.91–1.68 (m, 2H), 1.42–1.05 (m, 4H), 0.83 (t, *J* = 6.7 Hz, 3H). ^13^C NMR (50 MHz, CDCl_3_) δ 158.63, 154.69, 153.03, 150.82–150.78 (d, *J*_CF_ = 1.8 Hz), 149.98, 141.82 (2C), 135.08, 131.19 (2C), 129.05 (2C), 125.03, 124.74, 123.04–117.90 (d, *J*_CF_ = 257.7 Hz), 120.54, 117.72 (2C), 49.49 (2C), 44.38 (2C), 43.25, 29.36, 28.77, 22.10, 13.88. ^19^F NMR (188 MHz, CDCl_3_) δ −57.61 (3F). IR (KBr, cm^−1^): 2931, 1521, 1256, 1224, 1166. HRMS calcd. for (C_27_H_28_ClF_3_N_6_O [M + H]^+^): 545.2045. Found: 545.2031.

2-(4-(4-Chlorophenyl)piperazin-1-yl)-9-hexyl-6-(4-(trifluoromethoxy)phenyl)-9H-purine (**4m**) White solid, yield 81%, m.p. 126–128 °C. ^1^H NMR (400 MHz, CDCl_3_) δ 8.82 (d, *J* = 8.8 Hz, 2H), 7.79 (s, 1H), 7.36 (d, *J* = 8.4 Hz, 2H), 7.24 (d, *J* = 8.9 Hz, 2H), 6.93 (d, *J* = 8.8 Hz, 2H), 4.15–4.11 (m, 6H), 3.34–3.22 (m, 4H), 1.96–1.83 (m, 2H), 1.45–1.19 (m, 6H), 0.89 (t, *J* = 6.9 Hz, 3H). ^13^C NMR (101 MHz, CDCl_3_) δ 158.64, 154.70, 153.06, 150.82 (d, *J*_CF_ = 1.7 Hz), 149.94, 141.84, 135.09, 131.21 (2C), 129.08 (2C), 125.13, 124.75, 121.78–119.22 (d, *J*_CF_ = 258.6 Hz), 120.56 (2C), 117.78 (2C), 49.55 (2C), 44.39 (2C), 43.27, 31.18, 29.64, 26.32, 22.48, 13.99. ^19^F NMR (188 MHz, CDCl_3_) δ −57.60 (3F). IR (KBr, cm^−1^): 2931, 1529, 1270, 1224, 1159. HRMS calcd. for (C_28_H_30_ClF_3_N_6_O [M + H]^+^): 559.2200. Found: 559.2171. 

9-Pentyl-2-(4-(4-nitrophenyl)piperazin-1-yl)-6-(4-(trifluoromethoxy)phenyl)-9H-purine (**4n**) Yellow solid, yield 86%, m.p. 180–181 °C. ^1^H NMR (400 MHz, CDCl_3_) δ 8.82 (d, *J* = 8.8 Hz, 2H), 8.15 (d, *J* = 9.4 Hz, 2H), 7.82 (s, 1H), 7.36 (d, *J* = 8.3 Hz, 2H), 6.87 (d, *J* = 9.4 Hz, 2H), 4.24–4.03 (m, 6H), 3.69–3.55 (m, 4H), 2.00–1.85 (m, 2H), 1.45–1.28 (m, 4H), 0.91 (t, *J* = 7.0 Hz, 3H). ^13^C NMR (101 MHz, CDCl_3_) δ 158.34, 154.78, 154.64, 153.07, 150.85–150.83 (d, *J*_CF_ = 2.0 Hz), 141.96, 138.49, 134.90, 131.17 (2C), 125.98 (2C), 124.85, 121.72–119.26 (d, *J*_CF_ = 256.5 Hz), 120.53 (2C), 112.58 (2C), 46.83 (2C), 43.87 (2C), 43.28, 29.33, 28.74, 22.08, 13.87. ^19^F NMR (188 MHz, CDCl_3_) δ −57.60 (3F). IR (KBr, cm^−1^): 2925, 2859, 1598, 1316, 1230. HRMS calcd. for (C_27_H_28_F_3_N_7_O_3_ [M + H]^+^): 556.2286. Found: 556.2280.

9-Hexyl-2-(4-(4-nitrophenyl)piperazin-1-yl)-6-(4-(trifluoromethoxy)phenyl)-9H-purine (**4o**) Yellow solid, yield 90%, m.p. 142–144 °C. ^1^H NMR (400 MHz, CDCl_3_) δ 8.66 (d, *J* = 8.8 Hz, 2H), 7.99 (d, *J* = 9.3 Hz, 2H), 7.72 (s, 1H), 7.22 (d, *J* = 8.3 Hz, 2H), 6.72 (d, *J* = 9.3 Hz, 2H), 4.07–3.95 (m, 6H), 3.50–3.42 (m, 4H), 1.84–1.72 (m, 2H), 1.30–1.08 (m, 6H), 0.80–0.46 (m, 3H). ^13^C NMR (101 MHz, CDCl_3_) δ 158.41, 154.81, 154.59, 153.14, 150.93 (2C), 141.94, 138.57, 134.82, 131.22 (2C), 126.02, 124.48, 120.60 (2C), 112.64 (2C), 46.88 (2C), 43.91 (2C), 43.41, 31.18, 29.63, 26.32, 22.61, 22.48, 13.98. ^19^F NMR (188 MHz, CDCl_3_) δ −57.60 (3F). IR (KBr, cm^−1^): 2931, 2855, 1597, 1531, 1329, 1266, 1231. HRMS calcd. for (C_28_H_30_F_3_N_7_O_3_ [M + H]^+^): 570.2442. Found: 570.2424.

9-Pentyl-2-(4-(4-methoxyphenyl)piperazin-1-yl)-6-(4-(trifluoromethoxy)phenyl)-9H-purine (**4p**) Brown solid, yield 84%, m.p. 118–120 °C. ^1^H NMR (400 MHz, CDCl_3_) δ 8.83 (d, *J* = 8.9 Hz, 2H), 7.78 (s, 1H), 7.37 (d, *J* = 8.3 Hz, 2H), 6.99 (d, *J* = 9.0 Hz, 2H), 6.88 (d, *J* = 9.0 Hz, 2H), 4.27–4.05 (m, 6H), 3.78 (s, 3H), 3.35–3.06 (m, 4H), 2.08–1.77 (m, 2H), 1.46–1.21 (m, 4H), 0.91 (t, *J* = 7.1 Hz, 3H). ^13^C NMR (101 MHz, CDCl_3_) δ 158.70, 154.66, 154.09, 152.93, 150.72–150.70 (d, *J*_CF_ = 1.7 Hz), 145.81, 141.68, 135.13, 131.14 (2C), 124.61, 121.72–119.16 (d, *J*_CF_ = 257.7 Hz), 120.47 (2C), 118.68 (2C), 114.45 (2C), 55.48, 50.95 (2C), 44.64 (2C), 43.16, 29.30, 28.72, 22.05, 13.83. ^19^F NMR (188 MHz, CDCl_3_) δ −57.59 (3F). IR (KBr, cm^−1^): 2927, 2865, 1600, 1265. HRMS calcd. for (C_28_H_31_F_3_N_6_O_2_ [M + H]^+^): 541.2541. Found: 541.2514.

9-Hexyl-2-(4-(4-methoxyphenyl)piperazin-1-yl)-6-(4-(trifluoromethoxy)phenyl)-9H-purine (**4q**) Yellow solid, yield 80%, m.p. 144–145 °C. ^1^H NMR (400 MHz, CDCl_3_) δ 8.68 (d, *J* = 8.8 Hz, 2H), 7.65 (s, 1H), 7.22 (d, *J* = 8.3 Hz, 2H), 6.88 (d, *J* = 8.3 Hz, 2H), 6.74 (d, *J* = 9.0 Hz, 2H), 4.00 (t, *J* = 6.9 Hz, 6H), 3.64 (s, 3H), 3.13–3.04 (m, 4H), 1.82–1.72 (m, 2H), 1.29–1.07 (m, 6H), 0.75 (t, *J* = 6.9 Hz, 3H). ^13^C NMR (101 MHz, CDCl_3_) δ 158.15, 154.14, 152.48, 150.22 (d, *J*_CF_ = 1.4 Hz), 141.20 (2C), 134.57, 130.62 (2C), 124.13, 121.73, 121.20–118.64 (d, *J*_CF_ = 258.6 Hz), 119.98 (2C), 118.34 (2C), 113.99 (2C), 55.00, 50.62 (2C), 44.02 (2C), 42.68, 30.61, 29.07, 25.75, 21.90, 13.40. ^19^F NMR (188 MHz, CDCl_3_) δ −57.61 (3F). IR (KBr, cm^−1^): 2929, 1510, 1265, 1216, 1124. HRMS calcd. for (C_29_H_33_F_3_N_6_O_2_ [M + H]^+^): 555.2697. Found: 555.2668.

9-Pentyl-2-(4-(pyridin-4-yl)piperazin-1-yl)-6-(4-(trifluoromethoxy)phenyl)-9H-purine (**4r**) Yellow solid, yield 70%, m.p. 109–110°C. ^1^H NMR (400 MHz, CDCl_3_) δ 8.66 (d, *J* = 8.8 Hz, 2H), 7.99 (d, *J* = 9.3 Hz, 2H), 7.66 (s, 1H), 7.21 (d, *J* = 8.4 Hz, 2H), 6.71 (d, *J* = 9.4 Hz, 2H), 4.07–3.85 (m, 6H), 3.51–3.36 (m, 4H), 1.90–1.69 (m, 2H), 1.32–1.16 (m, 4H), 0.76 (t, *J* = 7.0 Hz, 3H). ^13^C NMR (101 MHz, CDCl_3_) δ 159.51, 158.71, 154.64, 152.93, 150.72–150.70 (d, *J*_CF_ = 1.6 Hz), 147.94, 137.48 (2C), 135.08, 124.62, 121.71–119.15 (d, *J*_CF_ = 258.7 Hz), 120.47 (2C), 113.40 (2C), 107.16 (2C), 45.11 (2C), 44.25 (2C), 43.16, 29.28, 28.69, 22.03, 13.81. ^19^F NMR (188 MHz, CDCl_3_) δ −57.60 (3F). IR (KBr, cm^−1^): 2926, 1531, 1265, 1228, 1158. HRMS calcd. for (C_26_H_28_F_3_N_7_O [M + H]^+^): 512.2387. Found: 512.2369.

9-Hexyl-2-(4-(pyridin-4-yl)piperazin-1-yl)-6-(4-(trifluoromethoxy)phenyl)-9H-purine (**4s**) Yellow solid, yield 67%, m.p. 153–155 °C. ^1^H NMR (400 MHz, DMSO-d6) δ 8.91 (d, *J* = 8.8 Hz, 2H), 8.30 (s, 1H), 8.20 (d, *J* = 5.2 Hz, 2H), 7.56 (d, *J* = 8.3 Hz, 2H), 6.88 (d, *J* = 6.0 Hz, 2H), 4.15 (t, *J* = 6.9 Hz, 2H), 3.99 (s, 4H), 3.50 (s, 4H), 1.90–1.80 (m, 2H), 1.28 (m, 6H), 0.85 (t, *J* = 6.8 Hz, 3H). ^13^C NMR (101 MHz, DMSO-d6) δ 158.36, 155.07, 154.93, 151.61, 150.38 (d, *J*_CF_ = 1.2 Hz), 150.27 (2C), 150.21, 144.38, 135.57, 131.65 (2C), 124.64, 121.25 (2C), 108.87 (2C), 45.59 (2C), 44.00 (2C), 43.02, 31.02, 29.29, 26.08, 22.39, 14.29. ^19^F NMR (188 MHz, DMSO-d6) δ −57.60 (3F). IR (KBr, cm^−1^): 2933, 2852, 1482, 1207. HRMS calcd. for (C_27_H_30_F_3_N_7_O [M + H]^+^): 526.2544. Found: 526.2529.

### 5.2. Biology: In Vitro Assays

#### 5.2.1. Materials

Cell culture media, foetal bovine serum, and penicillin-streptomycin (P/S) were purchased from Biological Industries. Vismodegib, cisplatin, etoposide, 5-FU, gemcitabine, 3-(4,5-dymethylthiazol-2-yl)-2,5-diphenyl tetrazolium bromide (MTT), propidium iodide (PI) and dimethyl sulfoxide (DMSO) were obtained from Sigma-Aldrich and AK Scientific. Human colon cancer HCT116 (ATCC^®^ CCL-247EMT™), pancreatic cancer BxPC-3 (ATCC^®^ CRL-1687™) and AsPC-1 (ATCC^®^ CRL-1682™), lung cancer NCI-H1975 (ATCC^®^ CRL-5908™), HEK293 (ATCC^®^ CRL-1573™) and B16F10 (ATCC^®^ CRL-6475™) cells were obtained from ATCC. Medulloblastoma Daoy cells were kindly provided by Dr. Verónica Palma, Universidad de Chile. HT29 cells were obtained from Dr. Juan Villena, Universidad de Valparaíso, Chile. Purine derivatives and control drugs were prepared as fresh DMSO solutions immediately prior to any experiment or stored at -20 °C in amber vials for additional experiments.

#### 5.2.2. Cell Culture and Treatment

Cells were cultured in DMEM-F12 or RPMI 1640 culture medium as appropriate and supplemented with 10% (*v*/*v*) foetal bovine serum (FBS) and 100 UI/mL penicillin-streptomycin and cultured at 37 °C under 5% CO_2_ in a humidified atmosphere. DMSO alone (<1%) was used as a control vehicle. Compounds dissolved in DMSO were brought to room temperature before use. Cell viability was determined by exclusion of trypan blue dye. Cell numbers were counted in a Neubauer chamber by light microscopy.

#### 5.2.3. Cytotoxicity Study

Cytotoxicity assays were performed by using the MTT reduction method as described previously [35]. Briefly, cancer cell lines were plated in flat-bottom 96-well plates at a cell concentration of 5 × 10^3^ cells/well for the fastest growing lines, such as HEK293 and HT29 cells and 1 × 10^4^ cells/well for more slowly growing lines, such as HCT116, H1975, BxPC-3, AsPC-1, B16-F10 and DAOY. Then, cells were incubated with the synthesised compounds at different concentrations (from 0.05 to 50 µM) in 200 µL with 10% FBS in RPMI 1640 or DMEM F12 culture medium at 37 °C for 72 h. Later, 10 µL of MTT were added at a final concentration of 0.5 mg/mL, incubated at 37 °C for 4 h, and then solubilised with 10% sodium dodecyl sulphate (SDS) in 0.1 mM HCl and incubated overnight at 37 °C. Formazan formation was measured at 570 nm in a multiwell plate reader (Cytation 5, Biotek Instruments, Winooski, VT, USA). Three independent biological replicates were performed.

In the case of experiments using nanoemulsions, cell proliferation was evaluated after treatments. To do this the complete medium containing treatments was removed and replaced with fresh culture medium supplemented with 10% tetrazolium compound of the MTS^®^ assay (CellTiter 96^®^ Aqueous Non-Radioactive Cell Proliferation Assay), according to the manufacturer’s instructions (Promega, Madison, WI, USA). The soluble formazan produced by live cells was detected as absorbance at 490 nm on a Multiscan Reader (Synergy-H4, Biotek). Background values contributed by excess of cell debris and bubbles were measured at 650 nm and were subtracted.

#### 5.2.4. Proliferation Assays

HT29 and HEK293 cells were resuspended at 1 × 10^6^/mL in DMEM F12 without FBS. A 5 mM CFSE stock solution carboxyfluorescein diacetate succinimidyl ester (Cell Proliferation Kit, Life Technologies, Carlsbad, CA, USA) in DMSO was added to a final concentration of 5 µM and incubated at 37 °C for 15 min protected from light. At the end of the incubation period, the cells were immediately washed twice in cold PBS 1X/no-FBS. Cells were washed three times in PBS 1×/10%-FBS and resuspended in the same medium and plated in 12-well plates at a density of 1 × 10^5^ cells/200 µL medium and grown overnight at 37 °C in a 5% CO_2_ humidified atmosphere. Then, the cells were treated with **4s**, vismodegib (19 µM) or control condition (1% DMSO staining CFSE, or 1% DMSO non-staining CFSE) during 24, 48 and 72 h. The cells were collected and then analysed by flow cytometry (BD; FACScanto II, Mountain View, CA, USA) and data were processed using FCS Express v6 software. Two independent biological replicates were performed.

#### 5.2.5. Cell Cycle Analysis

HT29 and HEK293 cells were plated in 6-well plates at a density of 2.0 × 10^5^ cells/1.5 mL/well and grown overnight at 37 °C in a 5% CO_2_ humidified atmosphere. Cells were treated with **4s** or vismodegib (19 µM) or 1% DMSO (vehicle) for 24 h. Aliquots of cells were collected, pelleted, washed, and fixed with ethanol (70% *v*/*v*) for at least 30 min at 4 °C. Cells were resuspended twice in PBS and, after centrifugation and elimination of the supernatant, they were finally resuspended in a PBS solution containing propidium iodide (PI; 50 mg/mL, Invitrogen), RNase A (20 mg/mL, Invitrogen). After a final incubation for 1 h in the dark at 37 °C, the PI signal was measured using a FACS Canto II Flow-cytometer (BD; Mountain View, CA) with a 488 nm excitation laser, captured and FACS DIVA was used as the acquisition software. The percentage of cells in each phase was analysed using the FCS Express v6 software. Two independent biological replicates were performed.

#### 5.2.6. Cell Viability Assessed by Annexin /PI Assay

HT29 cells were seeded in a 12-well plate (5 × 10^5^ cells per well) and incubated overnight in DMEM F12. Then they were treated with **4s** or vismodegib (19 µM each) for 3–12 h at 37 °C. The cells were collected and then processed for apoptosis analysis using the Annexin-V/PI Alexa Fluor 488 Apoptosis Kit (Invitrogen, Waltham, MA, USA) according to the manufacturer’s instructions. Finally, cells were analysed by flow cytometry (BD; FACS Canto II, Mountain View, CA, USA) and data were processed using the FCS Express v6 software. Three independent replicates were performed.

#### 5.2.7. Determination of Tumour Colony Formation

HT29, HCT116, H1975 and HEK293 cells were seeded in a 6-well plate (1 × 10^4^ cells per well) and incubated overnight in DMEM F12 supplemented with 10% FBS. Then, the medium was replaced with fresh medium containing the test compound **4s** or vismodegib at 5 µM. After 72 h, the medium was replaced by fresh medium without stimuli, and cells were incubated for an additional 14 d to permit colony formation. The cells were fixed during 30 min with PFA 4% (*v*/*v*), washed and stained for 30 min at RT with a 5% solution of violet crystal for final colony counting. Three independent replicates were performed.

#### 5.2.8. BODIPY-Cyclopamine Binding Assay

HEK293T cells were transfected with genes encoding human WT SMO tagged with Myc-DDK or human SMO D473H mutant tagged with Myc. Cells were washed in PBS supplemented with 0.5% FBS, fixed in 4% paraformaldehyde in PBS for 10 min, and incubated for 6 h at 37 °C in the same medium supplemented with BODIPY-cyclopamine (5 nM), with and without **4s** at different concentrations (1 nm at 5 µM). The cells were permeabilised with 0.2% Triton X-100 (Sigma, St. Louis, MO, USA). Dako Fluorescent Mounting solution (Dako, Carpinteria, CA, USA) was used as mounting medium and the Hoechst reagent for staining of cell nuclei. BODIPY (green) and Hoechst (blue) signals were analysed in three representative fields per coverslip (×20 magnification). Data were expressed as the percentage of BC incorporation observed with BC alone.

#### 5.2.9. Cell Cultures, Transfection and Treatments

HEK293T and *Smo*^−/−^, *Ptch*^−/−^ MEFs cells were cultured in DMEM plus 10% FBS, 1% L-glutamine and antibiotics. Transient transfections were performed using DreamFectTM Gold transfection reagent (Oz Biosciences SAS, Marseille, France) following the manufacturer’s instructions and for varying periods of time depending on the experiment (24–72 h). Cells were treated with BODIPY-cyclopamine (5 nM, BioVision Inc., San Francisco, CA, USA), and compound **4s** or vismodegib (1 μM, Selleckchem, Munich, Germany). Vehicle (DMSO) was used as control.

#### 5.2.10. HH-Dependent Luciferase Reporter Assay

The luciferase assay was performed in HCT116 cells, stably incorporating a GLI responsive luciferase reporter and the pRL-TK renilla construct for normalisation, were treated for 24 h with purmorphamine (20 µM), vismodegib (1 µM) or **4s** (1.5 µM). Luciferase and renilla activity were assayed with a dual-luciferase assay system according to the manufacturer’s instructions (Cignal Reporter Gli-luciferase, ADN, Qiagen, Hilden, Germany). Results were expressed as luciferase/renilla ratios and represent the mean of triplicate values in one assay. 

#### 5.2.11. Analysis of Gene Expression: mRNA levels

Total RNA was isolated with Trizol (Invitrogen/Life Technologies, Carlsbad, CA, USA) from *Smo*^−/−^ MEFs, *Ptch*^−/−^ MEFs or HT29 cells treated for 24 h and/or 48 h with **4s** at different concentrations and reverse transcribed with the SensiFAST cDNA Synthesis Kit (Bioline Reagents Limited, London, UK). Quantitative real-time PCR (Q-PCR) analysis of Gli1 and housekeeping (HPRT, TBP, HMBS) mRNA levels was performed on each cDNA sample using the VIIA7 Real Time PCR System employing Assay-on-Demand Reagents (Life Technologies) as previously described [51]. A reaction mixture containing cDNA template, SensiFAST Probe Lo-ROX Kit (Bioline Reagents Limited) and primer probe mixture was amplified using FAST Q-PCR thermal cycler parameters. Each amplification reaction was performed in triplicate, the average of the three threshold cycles was used to calculate the amount of transcript in the sample (using SDS version 2.3 Software). mRNA quantification was expressed in arbitrary units as the ratio of the sample quantity to the quantity of the calibrator (ΔCt method). All values were normalised with the endogenous controls HPRT, TBP and HMBS as appropriate and are described in each assay.

Primers used are:

Ptch1 F: 5′-CCA CAG AAG CGC TCC TAC A-3′
Ptch1 R: 5′-CTG TAA TTT CGC CCC TTC C-3′
GLi1 F: 5′-GGG ATG ATC CCA CAT CCT CAG TC-3′
GLi1 R: 5′-CTG GAG CAG CCC CCC CAG T-3′
HHIP F: 5′-CCC ACA CTT CAA CAG CAC CA-3′
HHIP R: 5′-GCT TTG TCA CAG GAC TTT GC-3′
TBP F: 5′-TGC ACA GGA GCC AAG AGT GAA-3′
TBP R: 5′-CAC ATC ACA GCT CCC CAC CA-3′
HMBS F: 5′-AAG TGC GAG CCA AGG ACC AG-3′
HMBS R: 5′-TTA CGA GCA TGA TGC CTA CCA AC-3′
HPRT F: 5′-GCT TCC TCC TCA GAC CGC TT-3′
HPRT R: 5′-GG TCA TAA CCT GGT TCA TCA TCG-3′

### 5.3. In Silico Studies: Molecular Docking

The crystallographic structure of the smoothened receptor complexed with a SMO antagonist agent (PDB ID: 4QIN) [52] was used as a receptor in molecular modelling studies. Small molecules **4s** and vismodegib were sketched and built with the minimum energy at level B3LYP/6-31G** using Spartan 10 [80], while conformational analyses were performed with AutoDock 4.2 [81] using a grid size of 60 × 60 × 60 Å and the Lamarckian genetic algorithm, allowing storage of up to 50 conformers for each molecule. The lowest complex energy was selected to analyse the resulting structures. Pymol [82] and Maestro [83] were used to describe 3D and 2D contacts in protein/ligand complexes.

### 5.4. Preparation and Physicochemical Characterisation of Nanoemulsions Loaded with ***4s*** (***4s***-NEM)

The nanoemulsions were obtained following the solvent displacement methods and incorporating the drugs into the lipid phase [58,59,60,61]. The preparation consists of adding an organic phase containing 125 μL of Miglyol 812 (density 0.945 g mL^−1^), 30 mg of phosphatidylcholine-enriched fraction of soybean lecithin (Epikuron 145 V), (phosphatidylcholine-enriched fraction of soybean lecithin), 1.5 mg of **4s**, 4.5 mL of ethanol and 5 mL of acetone, to an aqueous phase containing only Milli-Q water (20 mL). The resulting solution was evaporated in a rotary evaporator until a volume of 5 mL remained in order to eliminate the organic solvents from the mixture (ethanol and acetone) as well as to concentrate the **4s** compound. Formation of the nanoemulsion was instantaneous and spontaneous, as evidenced by the milky appearance of the mixture.

To define the maximum concentration of **4s** contained in the nanoemulsions, firstly, a supernatant separation was carried out, and the free molecules were determined by fluorescence. Only 4.7 ± 3% of **4s** in was detected in supernatants after 1 h when 2.5 mg of this compound into a final volume of 5 mL of NEM formulation (Supplementary Materials, Figure S5). This quantity of **4s** employed was the equivalent to the maximum concentration that did not lead to colloidal phase alteration of NEM. Higher amounts of **4s** flocculate NEM. Moreover, this formulation only maintains the colloidal condition for a few hours. For this reason, the stability over 21 d was studied using 2 mg or 1.5 mg of **4s**, to yield stable **4s**-NEM at the highest available concentration of **4s**. To define the stability, size and charge were evaluated 1.5 mg formulation was more stable and no changes in size (Supplementary Materials, Figure S6A) and in values of surface charge (Supplementary Materials, Figure S6B) were observed. Conversely, 2 mg formulation did, increase size by around 40% after 21 d, and decrease magnitude of *z*-potential [56]. The size and zeta potential of the colloidal systems were determined by photon correlation spectroscopy and laser Doppler anemometry, with a Zetasizer Nano-ZS (Malvern Instruments, Malvern, UK).

### 5.5. In Vivo Studies

#### 5.5.1. Animals

C57BL/6 mice were obtained from the Instituto de Salud Pública (Santiago, Chile) and housed in the animal facility of the Centro de Estudios en Ejercicio, Metabolismo y Cáncer (CEMC, Instituto de Ciencias Biomédicas, Universidad de Chile). A total of 42 C57BL/6 mice (18 males and 24 females) between 8 and 12 weeks of age and average weight of 25 g were employed and evenly distributed between experimental groups. All animal procedures were following the Guidelines for Care and Use of Laboratory Animals of the University of Chile and approved by the official Bioethics Committee (approved protocol register CBA#889). This bioethics Committee on Animal Research follows guidelines issued by the Institutional Program for the Care and Use of Animals (PICUA), and the Guide for the Use and Care of Laboratory Animals of the National Research Council of The National Academies (USA).

#### 5.5.2. Recurrent Tumour Growth and Lung Metastasis in Animal Models

Studies of subcutaneous tumour growth of B16F10 cells and metastasis assays of B16F10 cells in C57BL/6 mice were developed as previously described [56]. A single dose of **4s**-NEM (0.3 mg/mL) or controls (NEM and PBS) were administered after excising the tumour and before suturing the wound. Volumes to be employed were calculated by correlating tumour volumes with those used for in vitro cell viability studies. For example, in a typical in vitro study, 10 μL of **4s**-NEM were administered to each well (0.3 cm^2^ per well considering plates of 96 wells). Thus, if the area of a tumour was on average 2.5 cm^2^, the volume of **4s**-NEM added post-surgery was 83.3 μL (2.5 cm^2^/0.3 cm^2^ × 10 μL).

#### 5.5.3. Statistical Analysis

Statistical analyses were performed using the StatView 4.1 software (Abacus Concepts, Berkeley, CA, USA) and GraphPad 6.0. Statistical tests were appropriately chosen for each experiment. For all other experiments, *p*-values were determined using a parametric *t*-test or 2-way ANOVA Tukey’s multiple comparisons tests for in vitro assays, or a non-parametric Mann–Whitney test in the case of in vivo assays; statistical significance was set at 95% confidence (*p* < 0.05). Results were expressed as mean ± standard deviation (SD) from an appropriate number of experiments. 

## Figures and Tables

**Table 1:** In vitro cytotoxicity (IC_50_) and Selectivity Index (SI) values for maximally active derivatives of purine **4a**–**4s** on six cancer cell lines and one non-cancer cell line.

	IC_50_ (µM)												
Compound	HH-Dependent Cell Lines						HH-Independent Cell Lines						
	HCT116 ^a^	SI	HT29 ^b^	SI	Daoy ^d^	SI	H1975 ^c^	SI	AsPC-1 ^e^	SI	BxPC-3 ^f^	SI	HEK293 ^g^
**4a**	32 ± 0.3	1.6	>50	-	18 ± 0.2	2.7	34 ± 0.9	1.5	20.1 ± 0.3	2.5	>50	-	>50
**4b**	47 ± 0.9	1.1	>50	-	>50	-	30 ± 1.0	1.7	23.2 ± 0.2	2.2	>50	-	>50
**4c**	>50	-	>50	-	31 ± 0.9	0.9	19 ± 0.8	2.6	>50	1.0	>50	-	>50
**4e**	>50	-	>50	-	>50	-	23 ± 0.3	2.2	>50	1.0	>50	-	>50
**4h**	>50	-	>50	-	19 ± 1.1	2.6	39 ± 1.2	1.3	>50	1.0	>50	-	>50
**4r**	19 ± 0.8	2.6	>50	-	6.5 ± 0.4	7.7	7.7 ± 0.4	6.5	>50	1.0	>50	-	>50
**4s**	1.3 ± 0.4	38	15 ± 1.2	3.3	1.4 ± 0.2	36	1.5 ± 0.3	33	1.7 ± 0.05	>29	4.1 ± 0.9	12	>50
Vismodegib	>50	-	>50	-	>50	-	>50	-	>50	-	>50	-	>50
Etoposide	9.8 ± 0.5	0.9	-	-	-	-	40 ± 0.2	0.2	-	-	-	-	8.4 ± 0.7
Cisplatin	-	-	-	-	12 ± 0.7	1.0	-	-	-	-	-	-	12 ± 1.4
5-FU	31 ± 0.3	0.03	0.51 ± 0.06	1.6	-	-	-	-	-	-	-		0.83 ± 0.07
Gemcitabine	-	-	-	-	-	-	-	-	0.3 ± 0.09	25	0.2 ± 0.07	38	7.5 ± 0.6

^a,b^ Human colon cancer cells. ^c^ Human lung cancer cells. ^d^ Human medulloblastoma cells. ^e,f^ Human pancreatic carcinoma cells. ^g^ Human embryonic kidney non-cancer cell line. IC_50_ = values were obtained from three separate experiments performed in triplicate in the concentration range of 0.05–50 μM of the test compounds. SI = IC_50_ of pure compound in the non-cancer cell line/IC_50_ of pure compound in the cancer cell line. In the case IC_50_ value on HEK was > 50, it was considered as 50 μM.

## Data Availability

Not applicable.

## Supplementary Materials

The following are available online at https://www.mdpi.com/article/10.3390/ijms22168372/s1.

## Abbreviations

SMO	Smoothened receptor
HH	Hedgehog
GLI1	Glioma1
SHH	Sonic Hedgehog
IHH	Indian Hedgehog
DHH	Desert Hedgehog
PTCH1	patched1
BCL2	B-cell lymphoma 2
c-MYC	c-MYC oncogene
SNAIL	Snail transcriptional factor
BMI1	B lymphoma Mo-MLV insertion region 1
BCC	basal cell carcinoma
MB	medulloblastoma
CDKs	cyclin-dependent kinases
Src	Src oncogene
VEGFR2	vascular endothelial growth factor receptor-2
5-FU	5-fluorouracil
MTT	3-(4,5-dymethylthiazol-2-yl)-2,5-diphenyl tetrazolium bromide
CFSE	carboxyfluorescein diacetate succinimidyl ester
PI	propidium iodide
HPRT	hypoxanthine guanine phosphoribosyltransferase
MEFs	murine embryonic fibroblasts
BC	BODIPY-cyclopamine
**4s**-NEM	**4s**-nanoemulsion
Ras-MEK/AKT	Ras-MEK/AKT signal transduction pathway
