# Supplementary material for: A New Smoothened Antagonist Bearing the Purine Scaffold Shows Antitumour Activity In Vitro and In Vivo
Source: Int J Mol Sci. 2021 Aug 4;22(16):8372. doi: 10.3390/ijms22168372 (PMC8395040; doi:10.3390/ijms22168372)
Supplement: Supplementary file 1 [file ijms-22-08372-s001.zip › ijms-1311410-supplementary.pdf]

Supplementary data

A new smoothened antagonist bearing the purine scaffold shows antitumor activity *in vitro* and *in vivo*

Ana María Zárate,[1] Christian Espinosa-Bustos,[2] Simón Guerrero,[3,4] Angélica Fierro,[1] Felipe Oyarzún-Ampuero,[3,5] Andrew F. G. Quest, [3,6] Lucia Di Marcotullio,[7] Elena Loricchio,[8] Miriam Caimano,[9] Andrea Calcaterra,[10] Matías González-Quiroz,[11] Adam Aguirre,[12] Jaime Meléndez,[2*] and Cristian O. Salas[1*]

[1] Departamento de Química Orgánica, Facultad de Química y de Farmacia, Pontificia Universidad Católica de Chile, Vicuna Mackenna 4860, Macul, 702843, Santiago, Chile.

[2] Departamento de Farmacia, Facultad de Química y de Farmacia, Pontificia Universidad Católica de Chile, Vicuna Mackenna 4860, Macul, 702843, Santiago, Chile.

[3] Advanced Center for Chronic Diseases (ACCDiS), Universidad de Chile, Sergio Livingstone 1007, Independencia, 8380492, Santiago, Chile.

[4] Instituto de Investigación Interdisciplinar en Ciencias Biomédicas, Universidad SEK (I3CBSEK). Facultad de Ciencias de la Salud, Fernando Manterola 0789, Providencia, 7520317, Santiago, Chile.

[5]Departamento de Ciencias y Tecnología Farmacéuticas, Facultad de Ciencias Químicas y Farmacéuticas, Universidad de Chile, Santos Dumont 964, Independencia, 8380494, Santiago, Chile.

[6]Laboratorio de Comunicaciones Celulares, Centro de Estudios en Ejercicio, Metabolismo y Cáncer (CEMC), Program of Cellular and Molecular Biology, Instituto de Ciencias Biomédicas (ICBM), Facultad de Medicina, Universidad de Chile. Independencia 1027, 8380453, Santiago, Chile.

[7] Department of Molecular Medicine, Laboratory affiliated to Insituto Pasteur Italia, Fondazione Cenci Bolognetti, Sapienza University of Rome, Viale Regina Elena 291, 00161 Rome, Italy.

[8] Center For Life Nano Science@Sapienza, Istituto Italiano di Tecnologia, Viale Regina Elena, 291 00161 Roma, Italy.

[9] Department of Molecular Medicine, Sapienza University of Rome, Viale Regina Elena 291, 00161 Rome, Italy.

[10] Department of Chemistry and Technology of Drugs, Sapienza University of Rome, Piazzale Aldo Moro 5, 00185 Rome, Italy.

[11] Biomedical Neuroscience Institute (BNI), Faculty of Medicine, Universidad de Chile, Santiago, Chile. Center for Geroscience, Brain Health and Metabolism (GERO), Santiago, Chile. Program of Cellular and Molecular Biology, Institute of Biomedical Sciences, Universidad de Chile, Independencia 1027, 8380453, Santiago, Chile.

[12] Laboratorio de Medicina Traslacional, Fundación Arturo López Pérez, Rancagua 878, Lower fifth floor, Providencia 8320000, Santiago, Chile.

* Authors to whom correspondence should be addressed; E-Mails: jgmelend@uc.cl; cosalas@uc.cl; Tel.: +56-2-2354-4427; Fax: +56-2-354-4474.

Table of Contents

Index

^{1}H NMR and ^{13}C NMR spectra of compounds **4a-s**..pp 3-21

HRMS spectra of compounds **4a-s**..pp 22-31

Table S1 ....pp 32

Figure S1-S9………………………………………..……………….……….... pp 33-39

Table S2: PAIN´s report for purine derivatives **4a-s**……………………………………pp 40

^{1}H NMR of compound **4a**

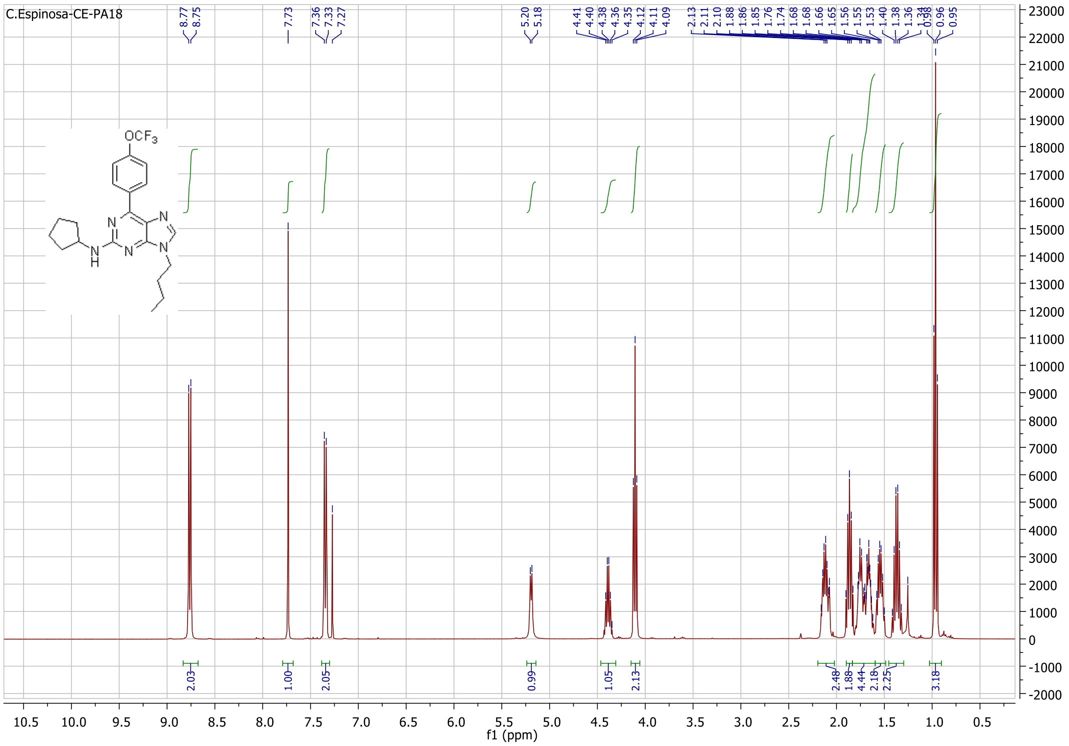

^{13}C NMR of compound **4a**

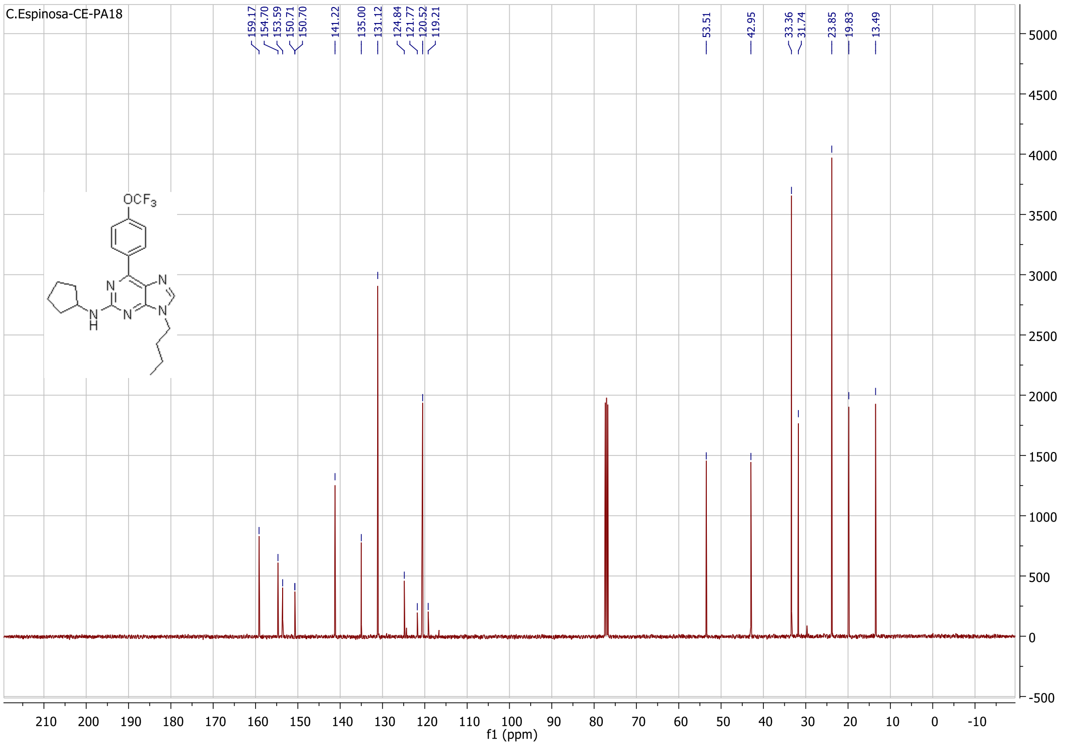

^{1}H NMR of compound **4b**

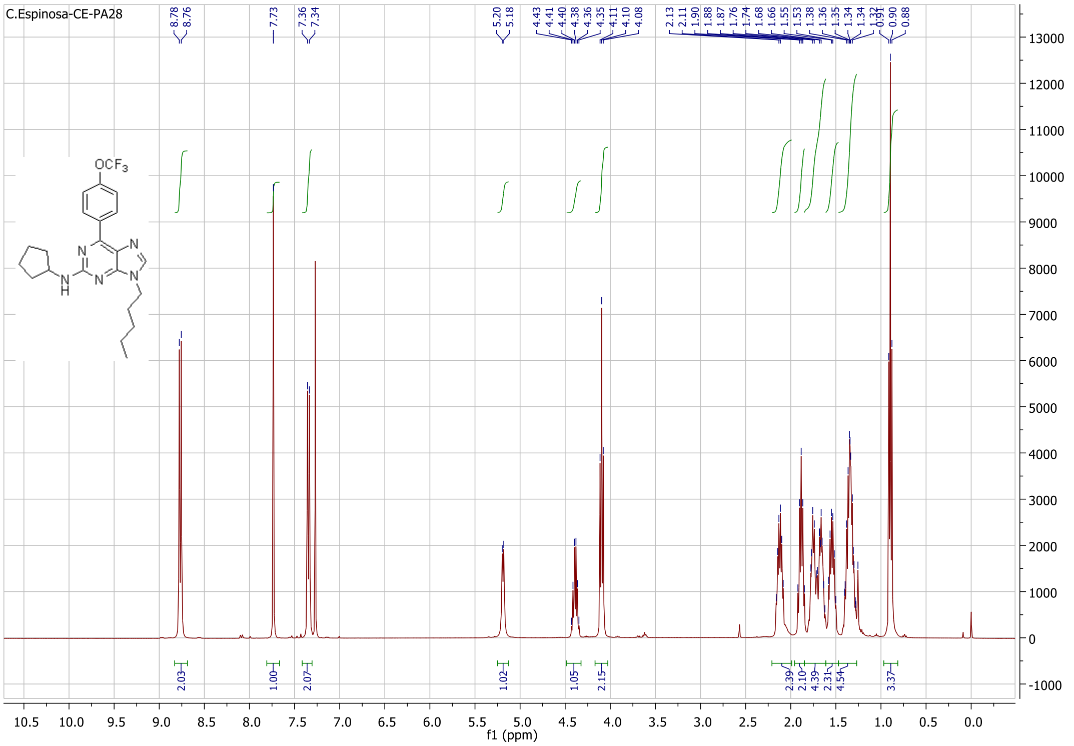

^{13}C NMR of compound **4b**

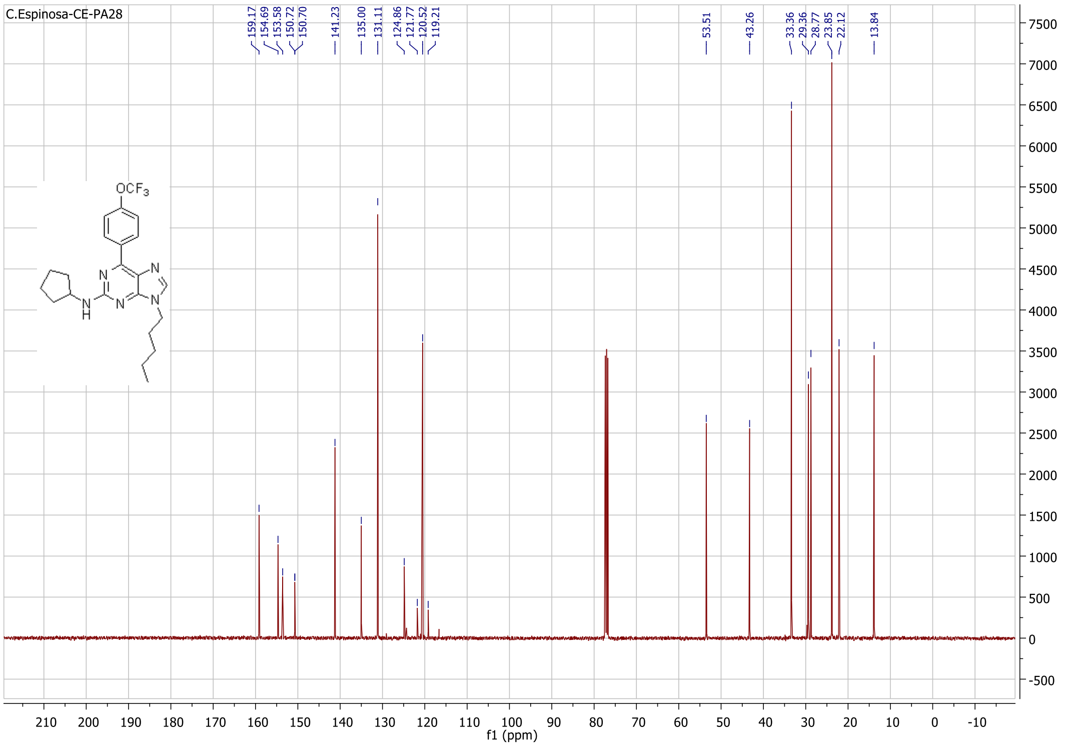

^{1}H NMR of compound **4c**

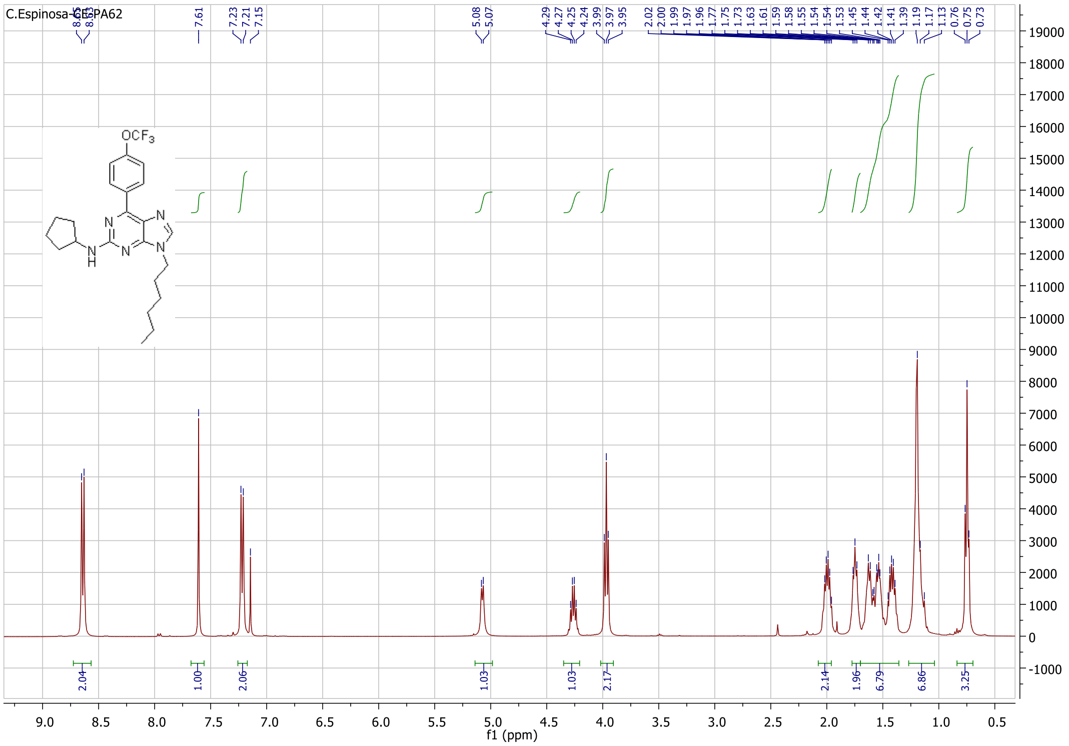

^{13}C NMR of compound **4c**

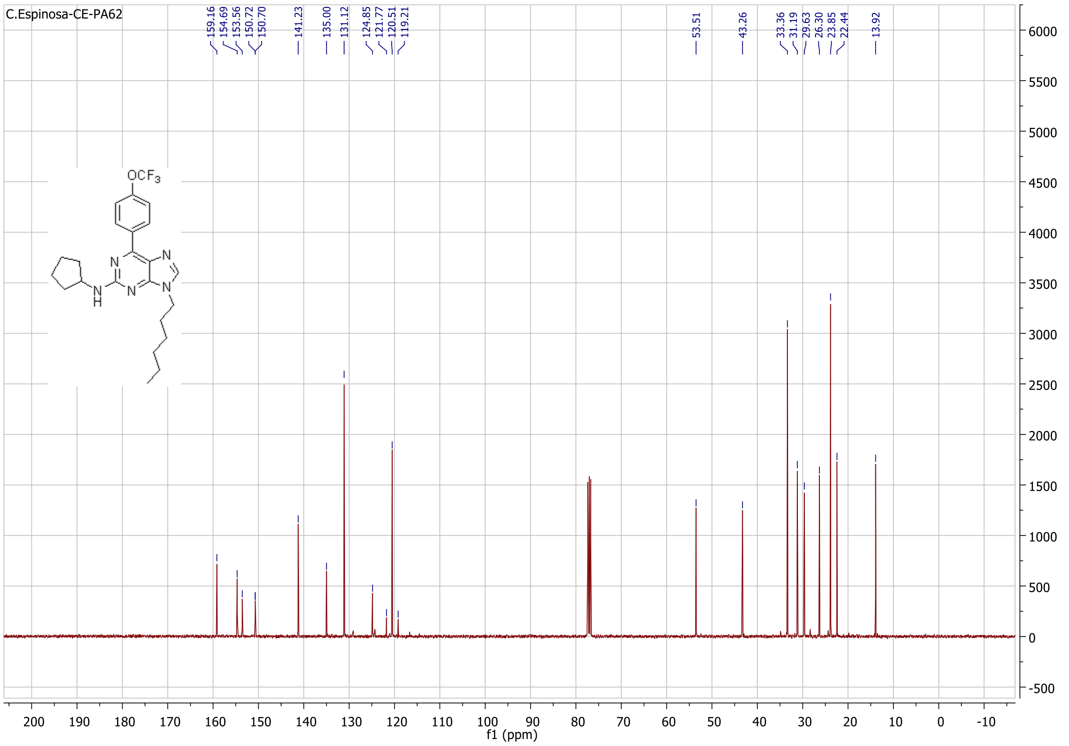

^{1}H NMR of compound **4d**

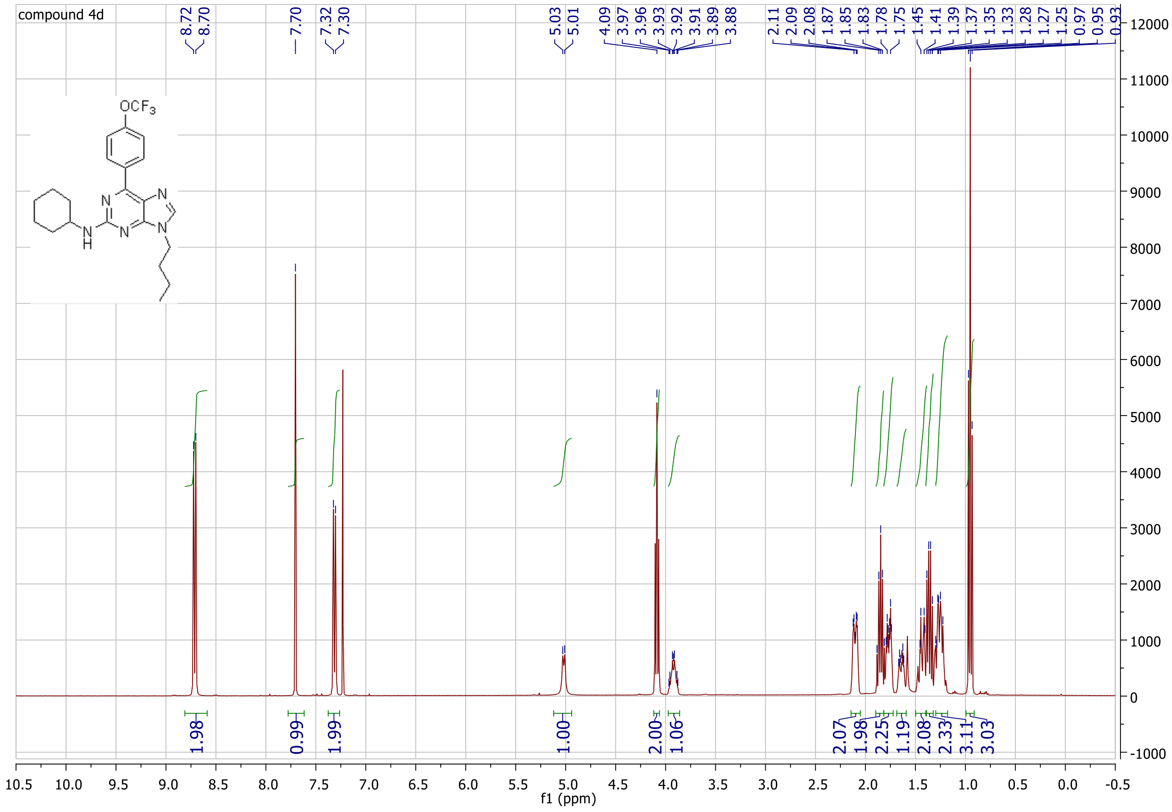

^{13}C NMR of compound **4d**

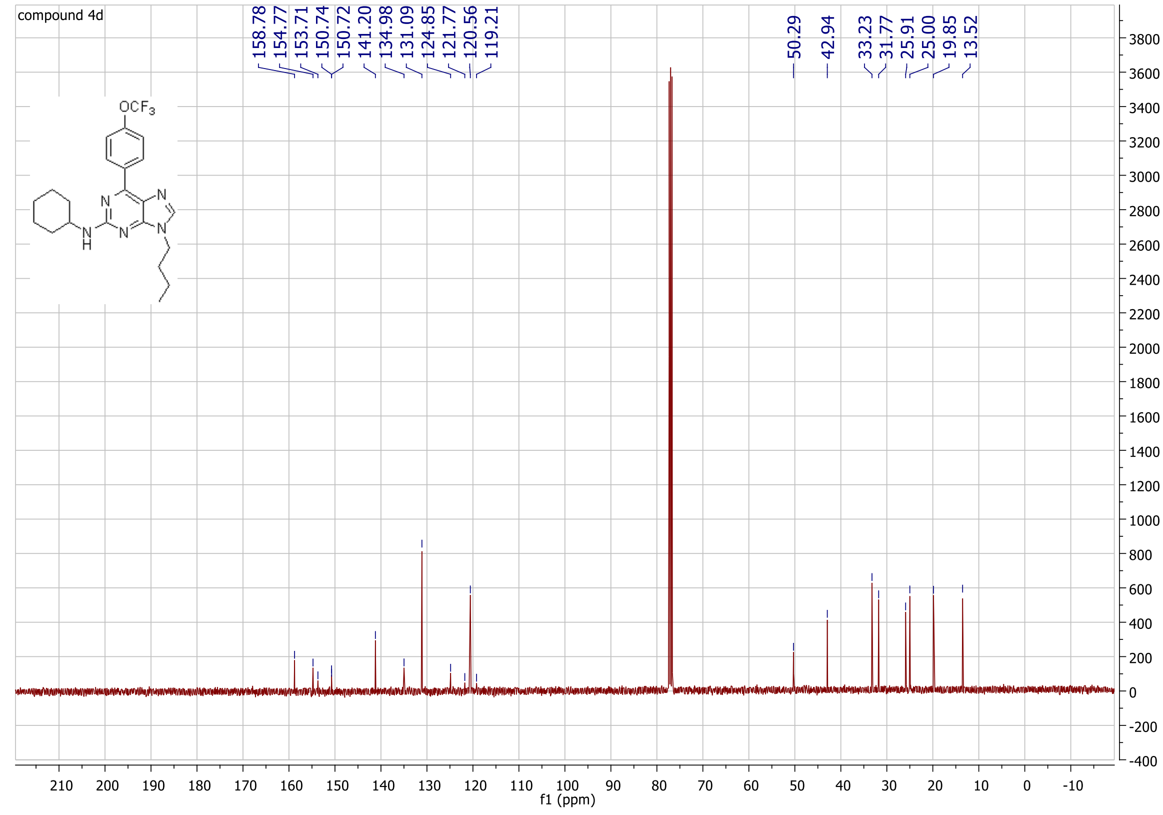

^{1}H NMR of compound **4e**

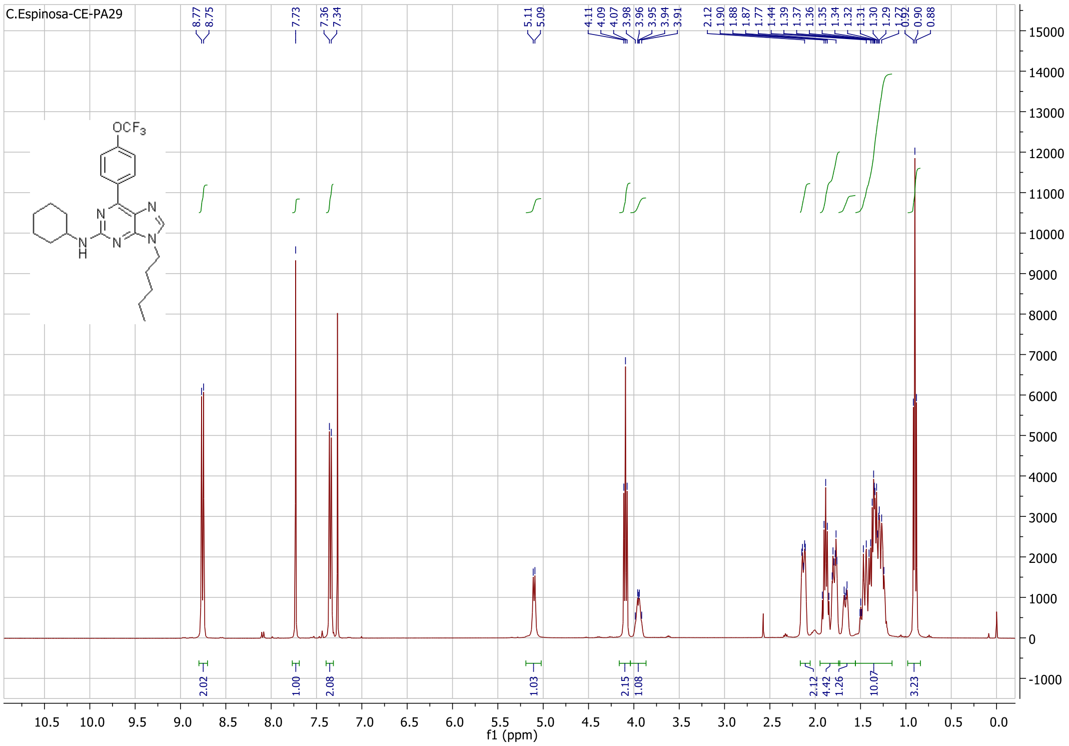

^{13}C NMR of compound **4e**

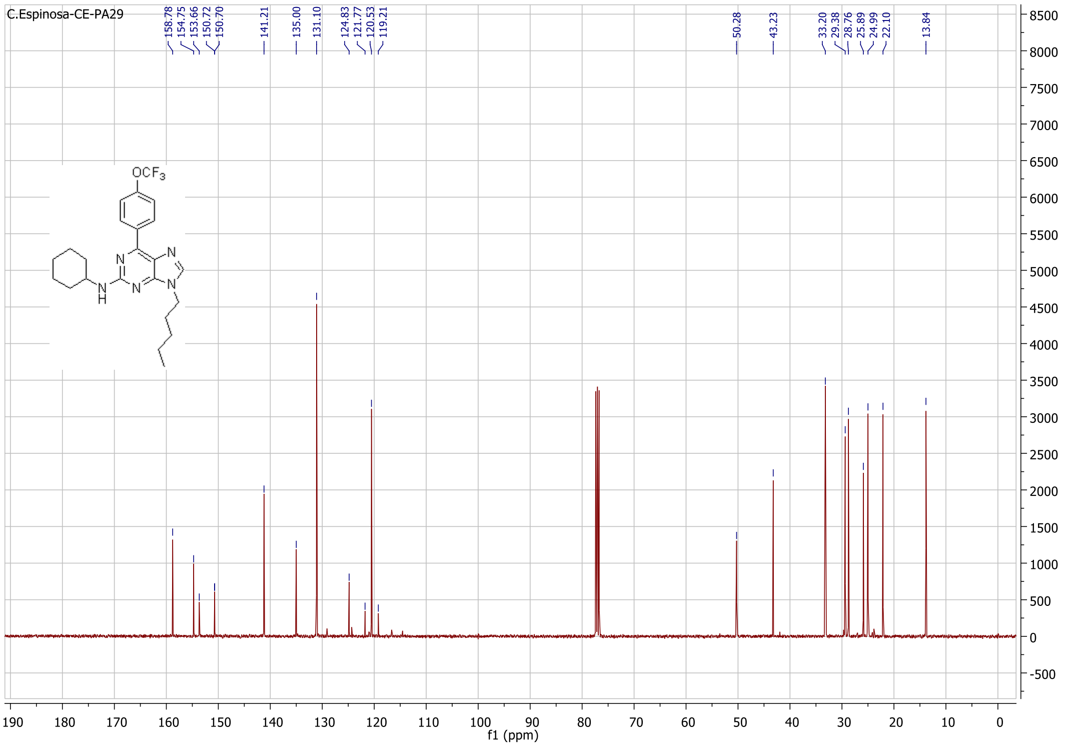

^{1}H NMR of compound **4f**

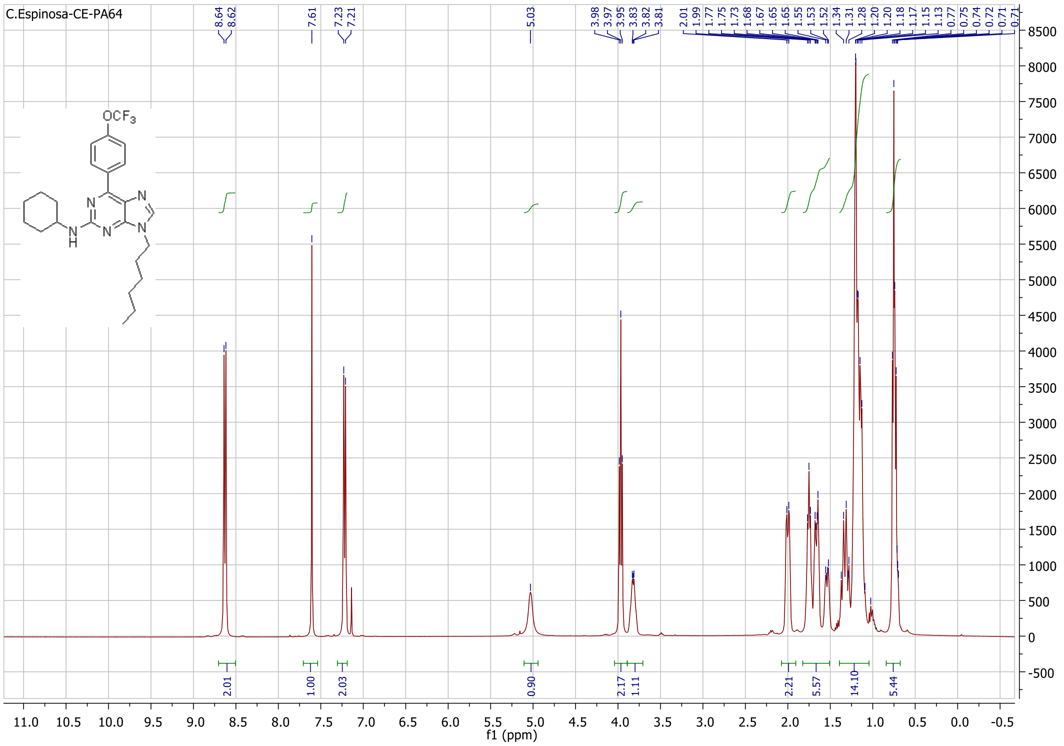

^{13}C NMR of compound **4f**

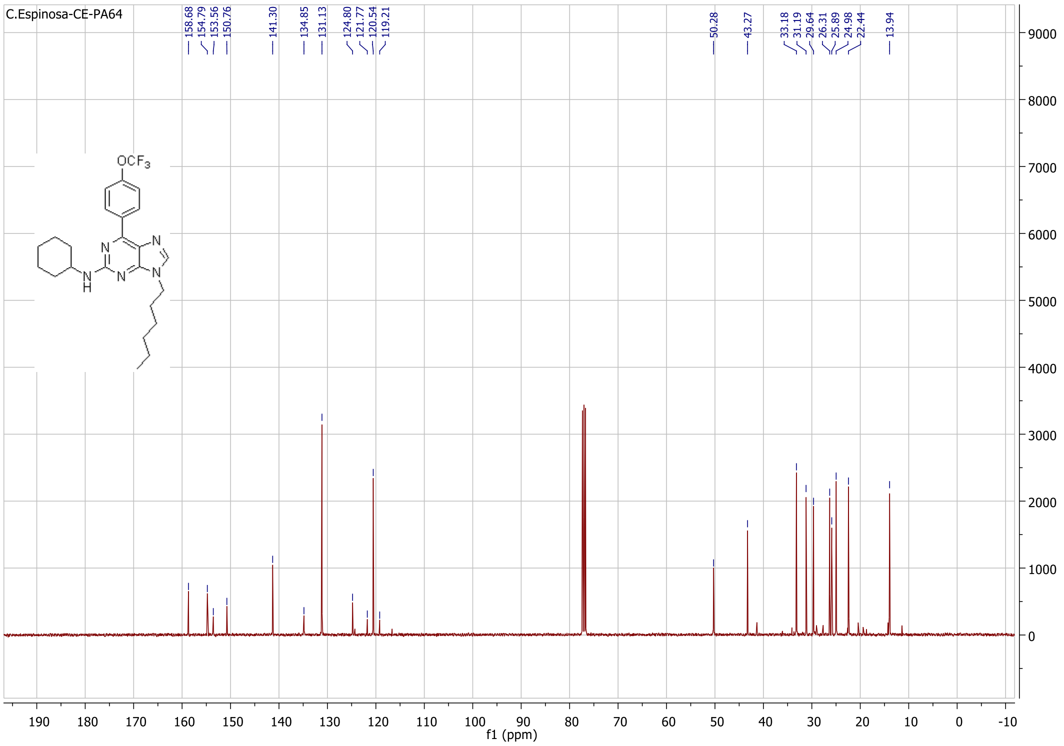

^{1}H NMR of compound **4g**

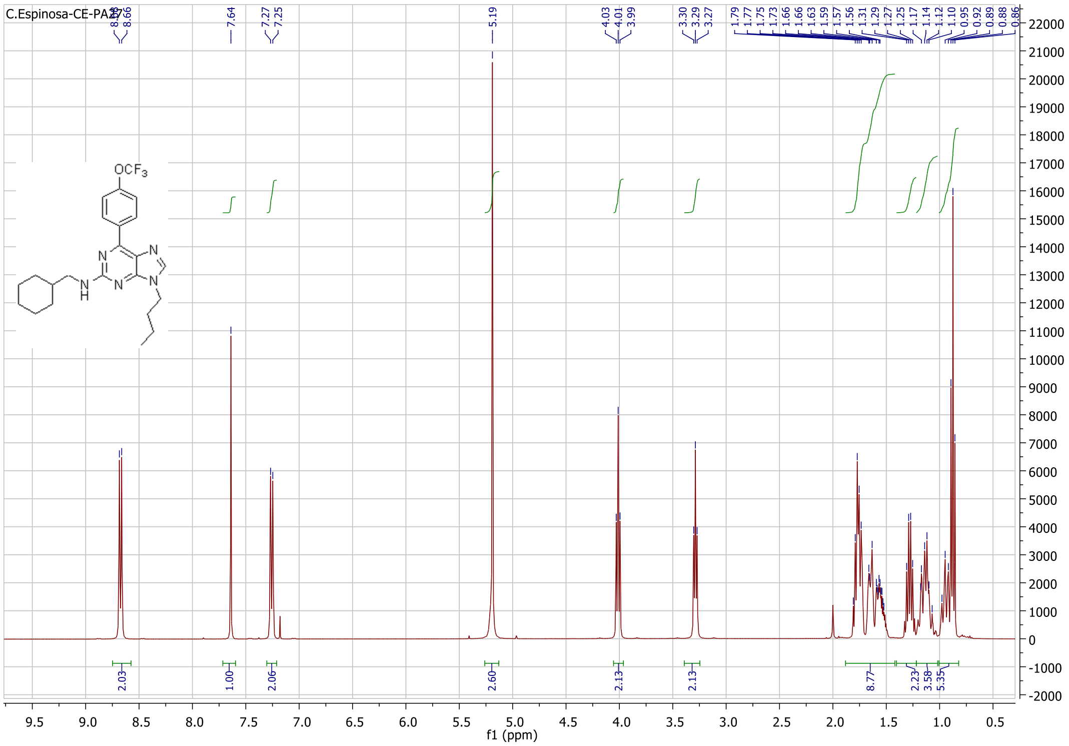

^{13}C NMR of compound **4g**

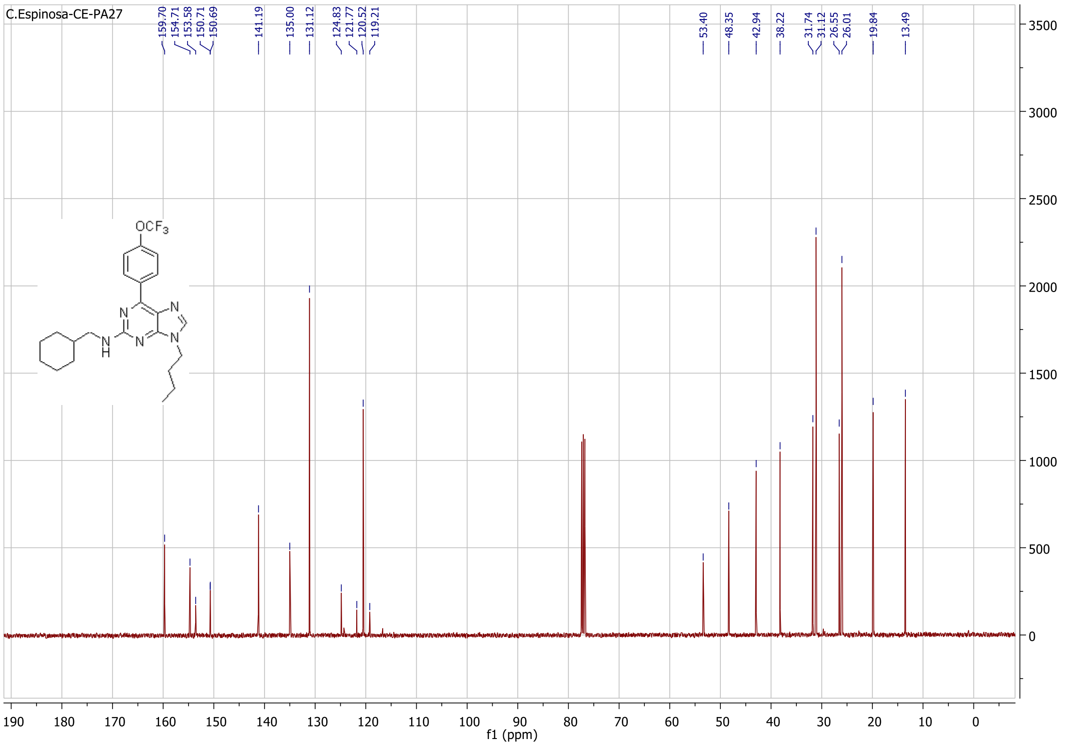

^{1}H NMR of compound **4h**

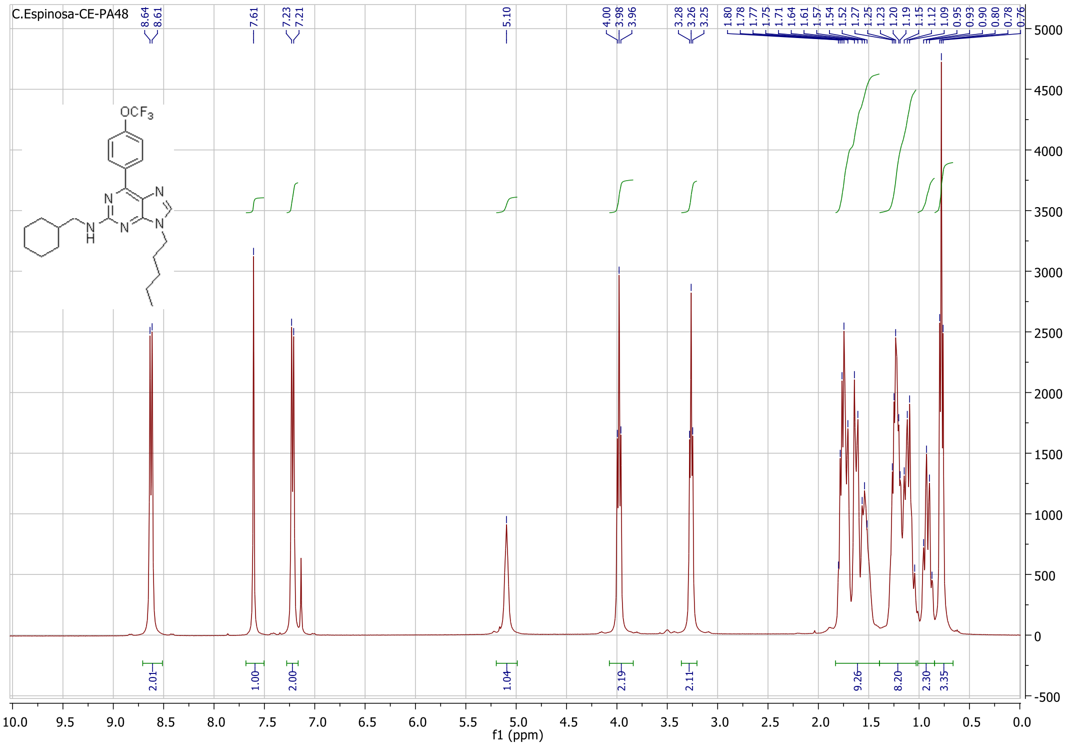

^{13}C NMR of compound **4h**

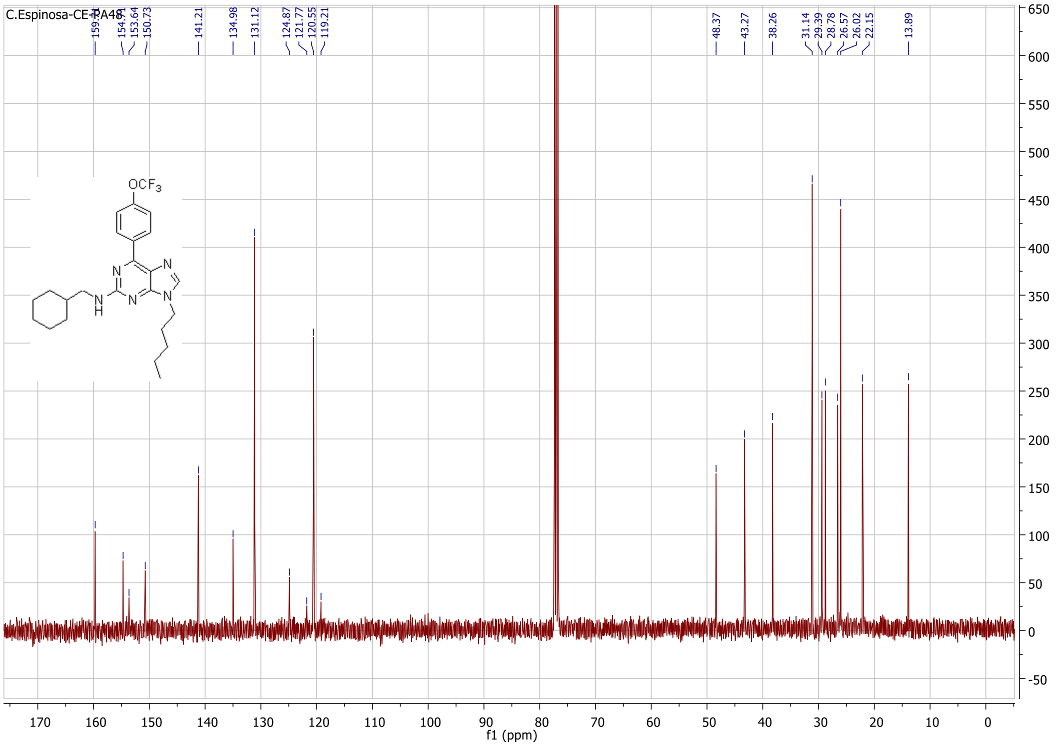

^{1}H NMR of compound **4i**

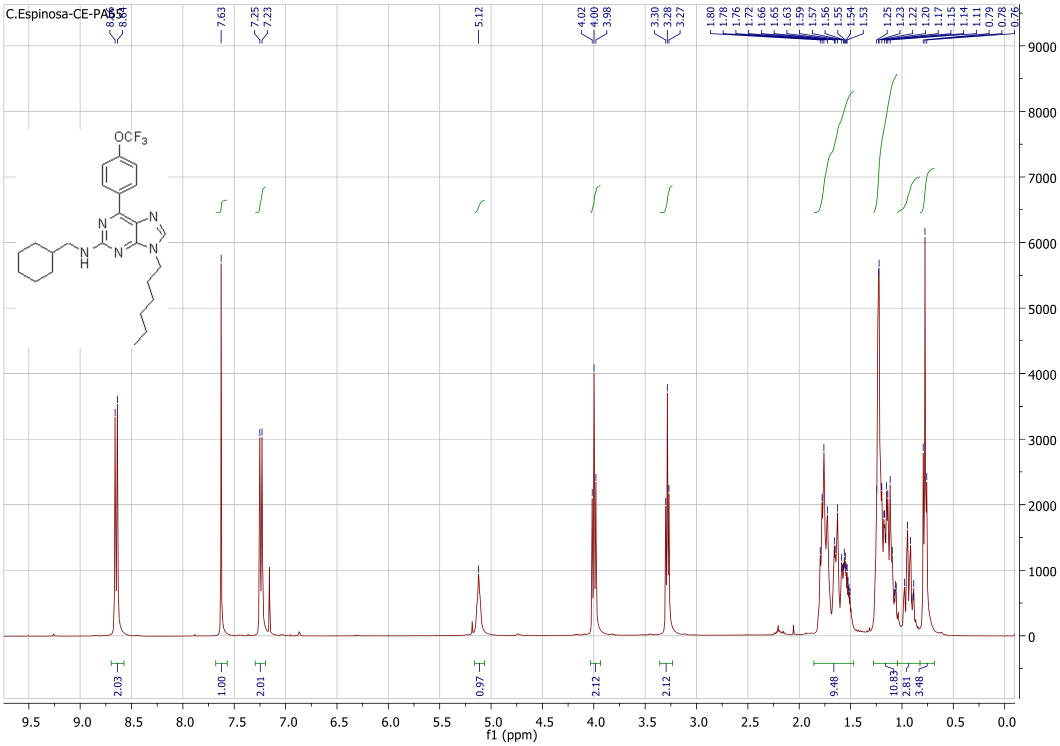

^{13}C NMR of compound **4i**

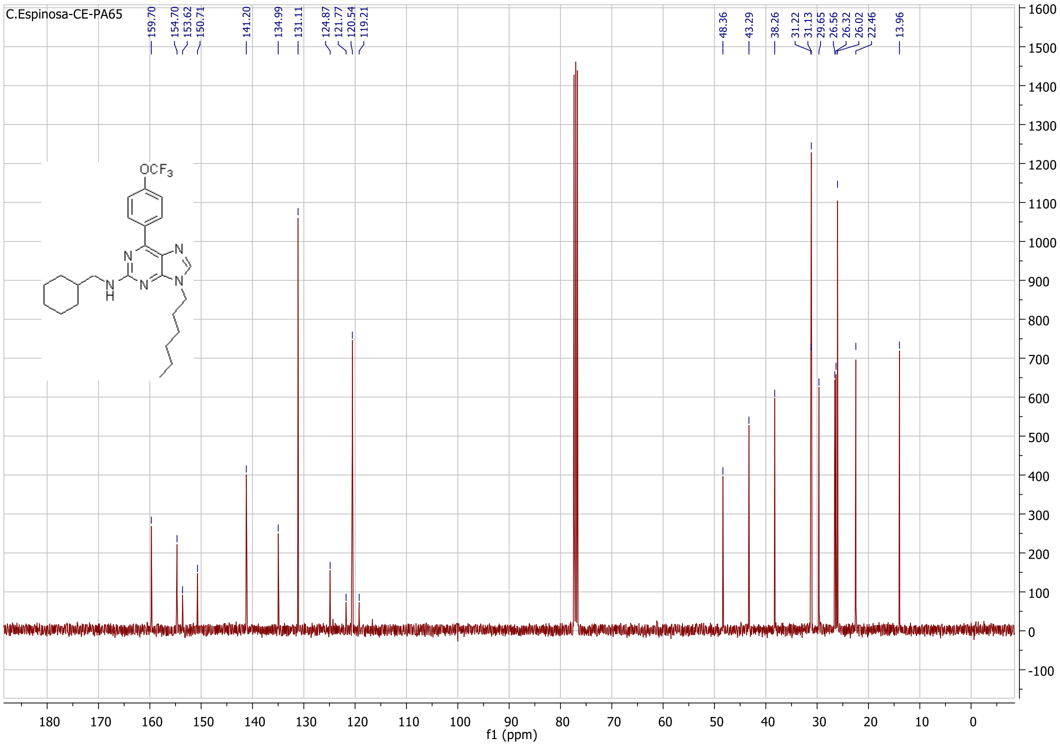

^{1}H NMR of compound **4j**

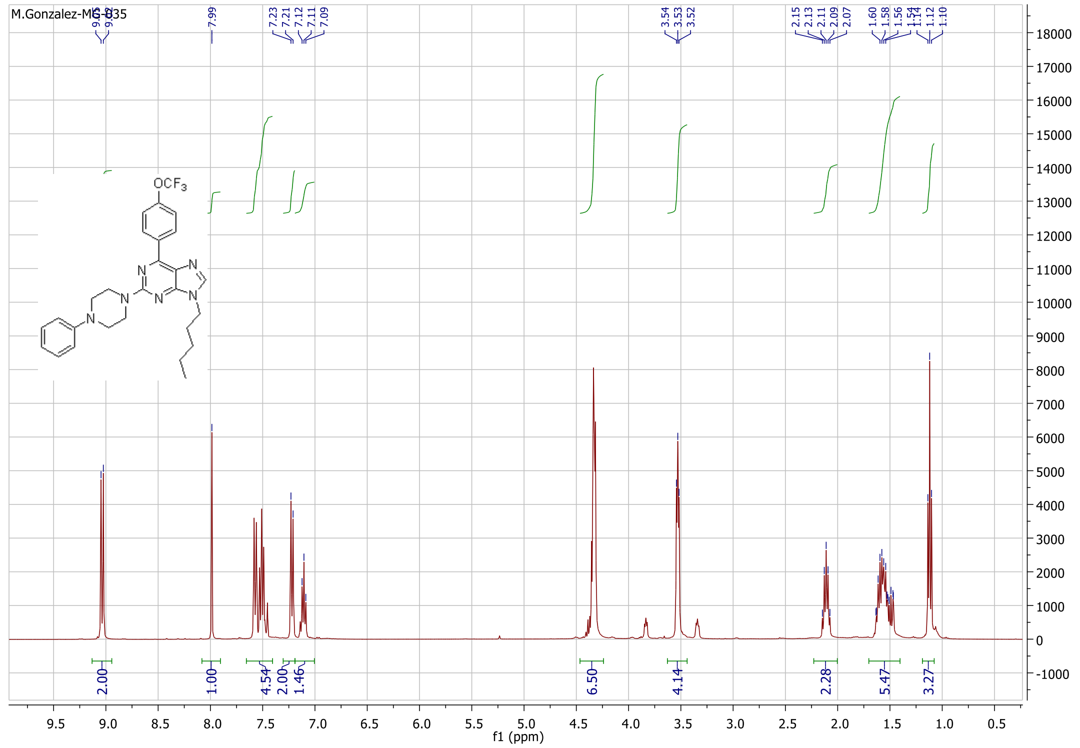

^{13}C NMR of compound **4j**

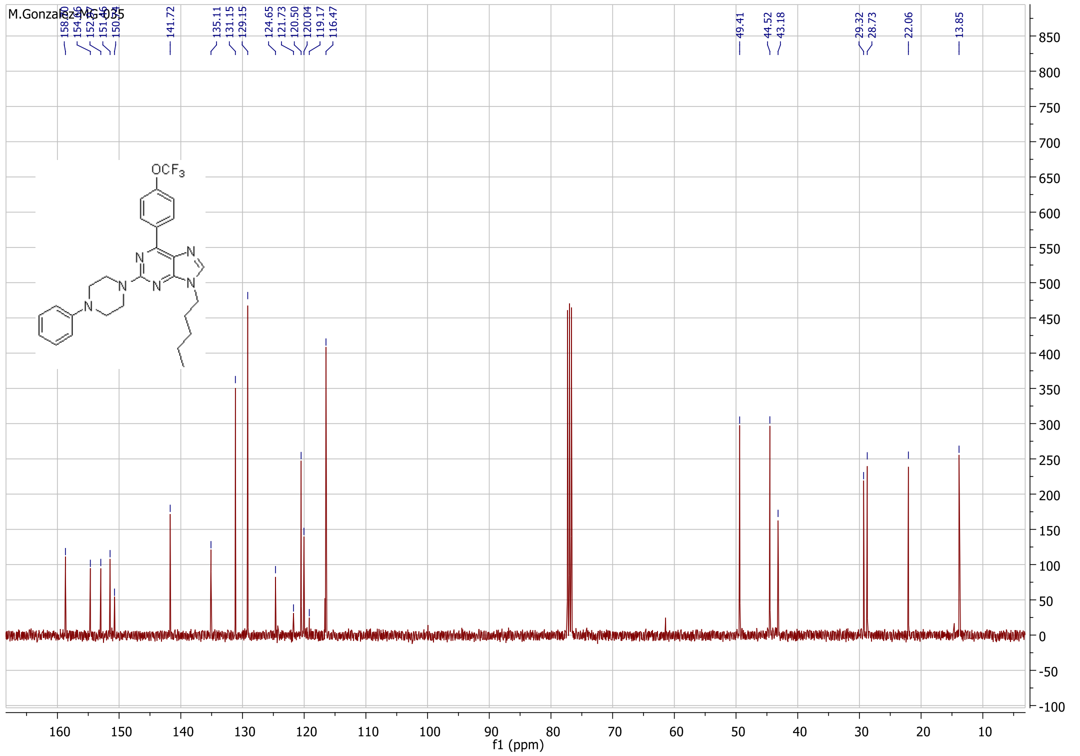

^{1}H NMR of compound **4k**

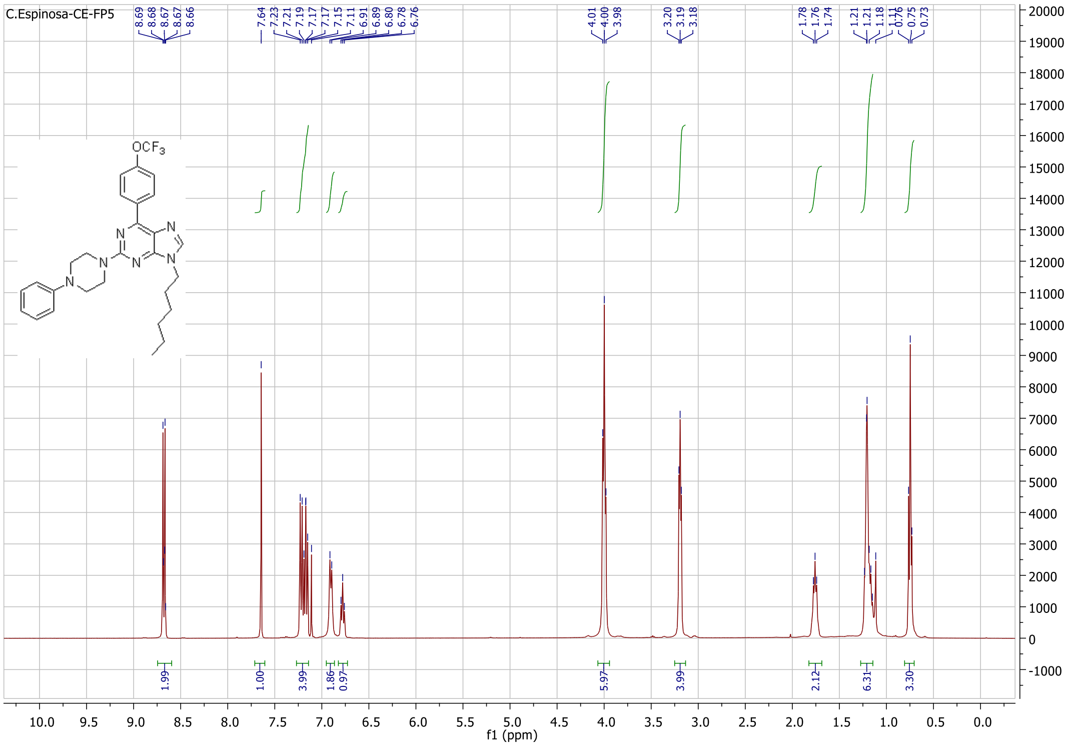

^{13}C NMR of compound **4k**

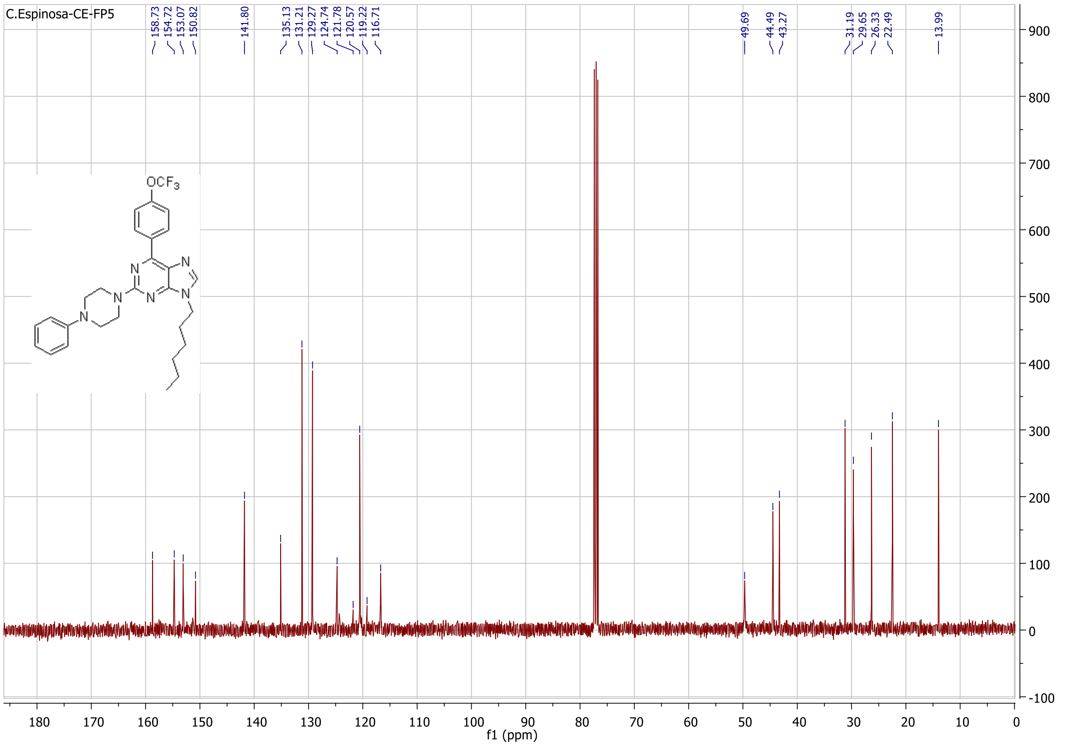

^{1}H NMR of compound **4l**

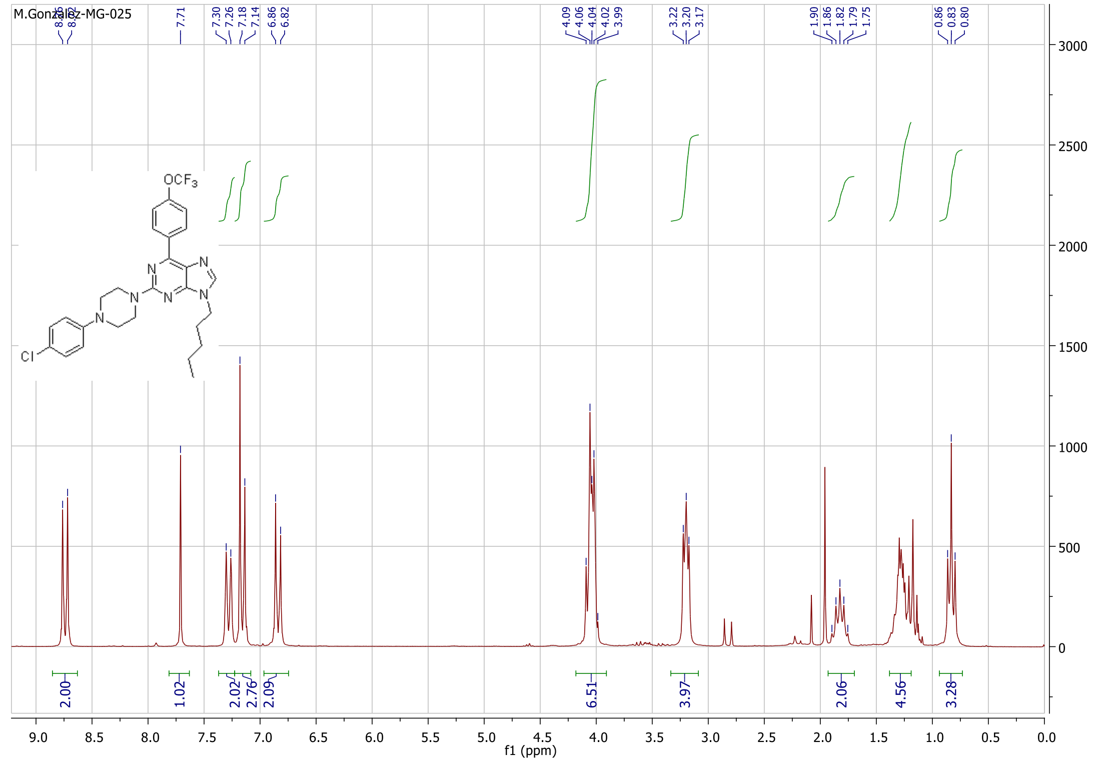

^{13}C NMR of compound **4l**

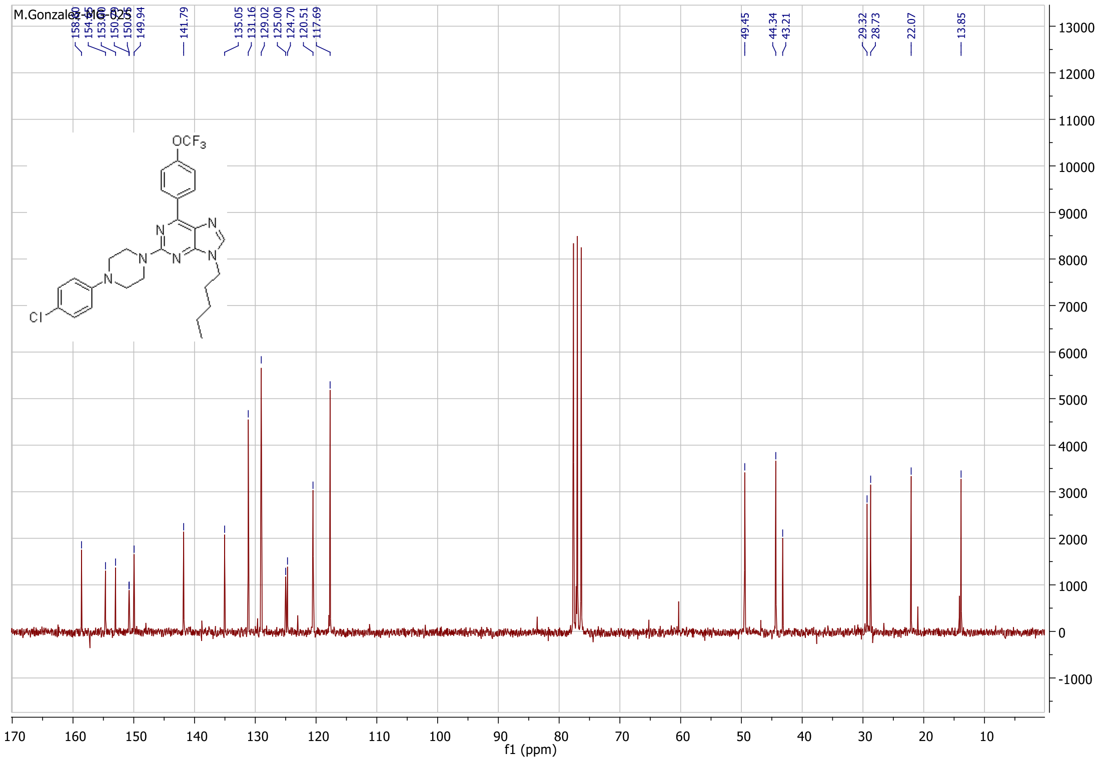

^{1}H NMR of compound **4m**

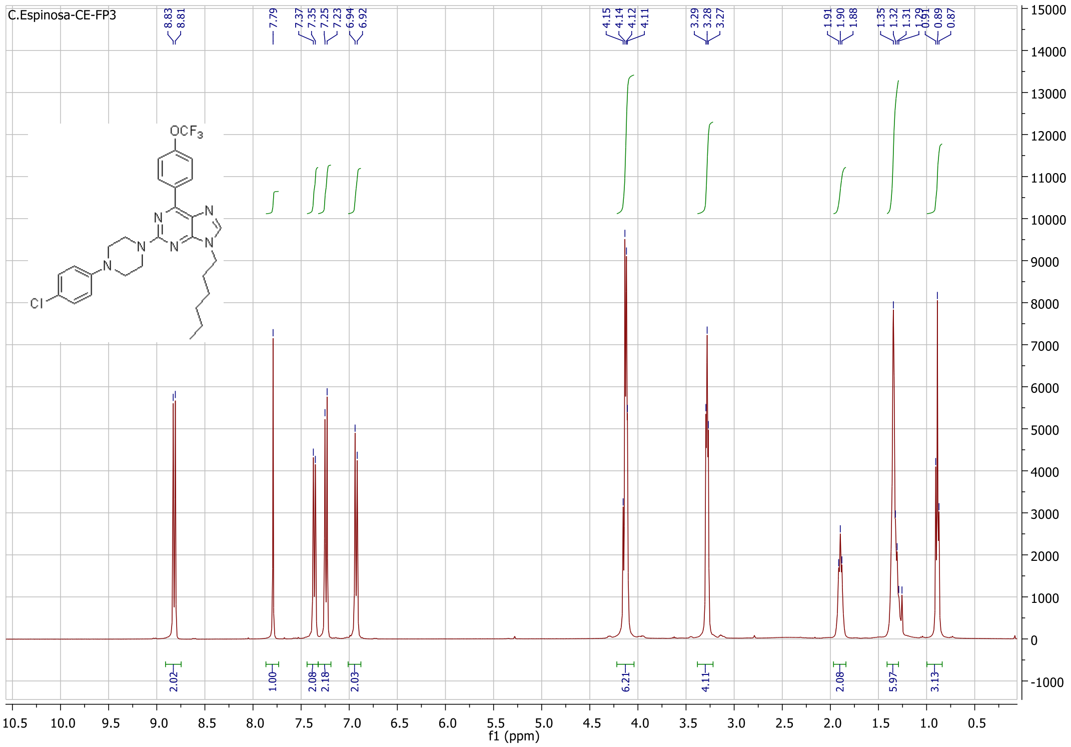

^{13}C NMR of compound **4m**

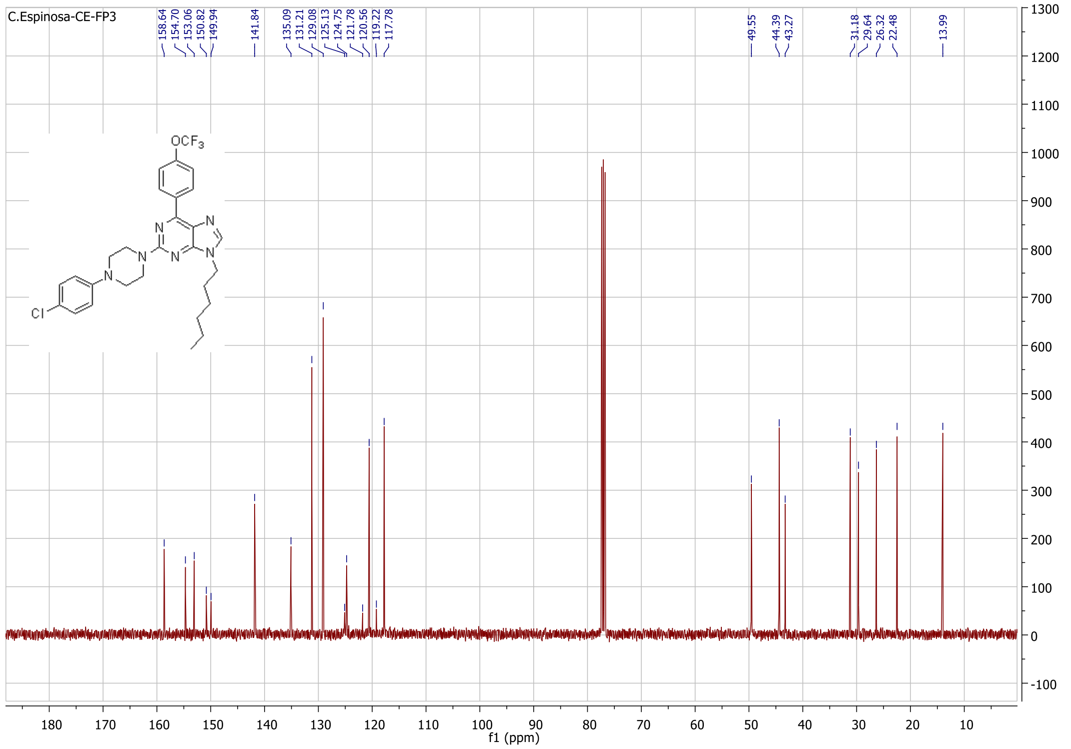

^{1}H NMR of compound **4n**

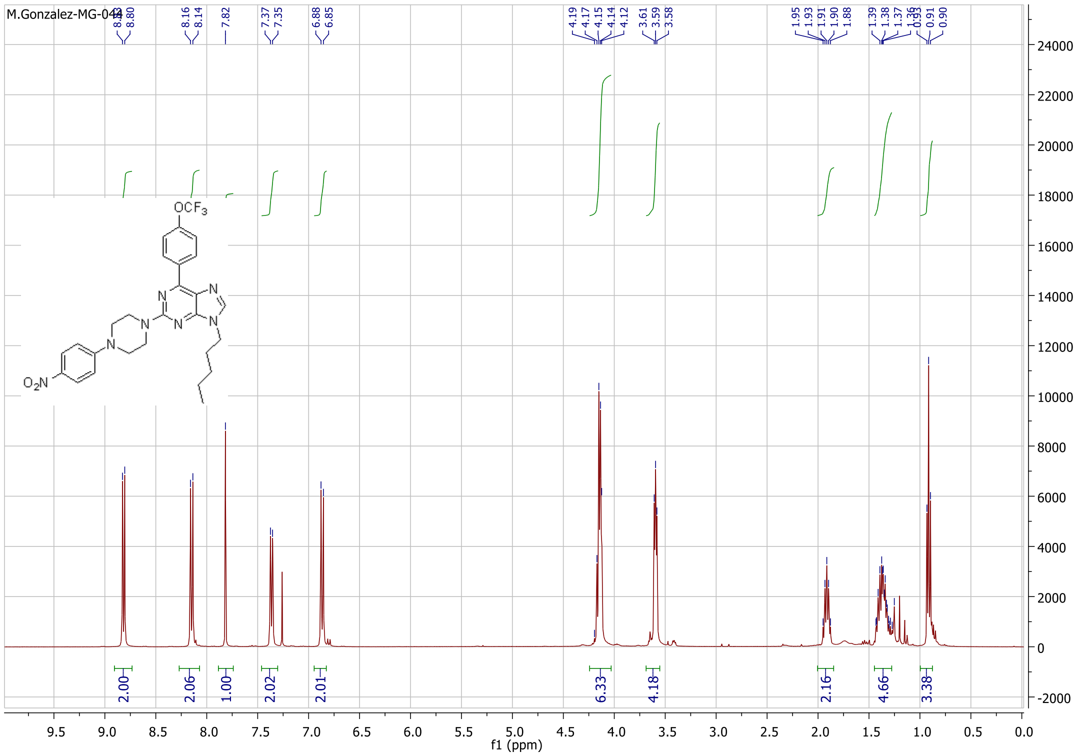

^{13}C NMR of compound **4n**

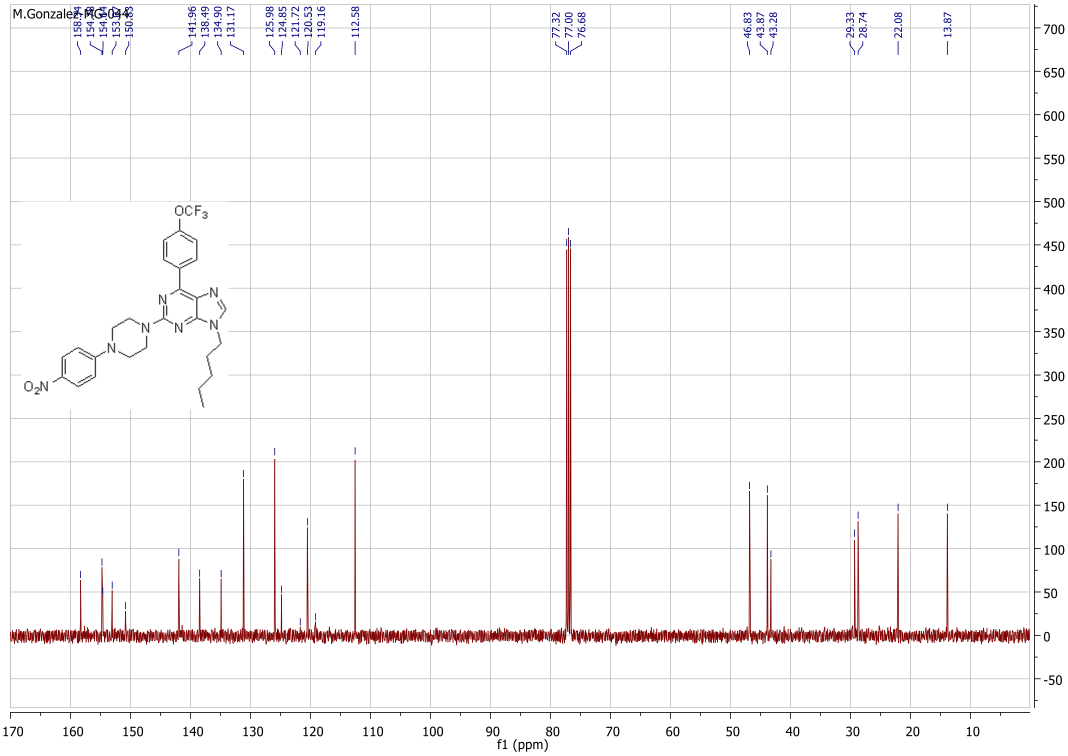

^{1}H NMR of compound **4o**

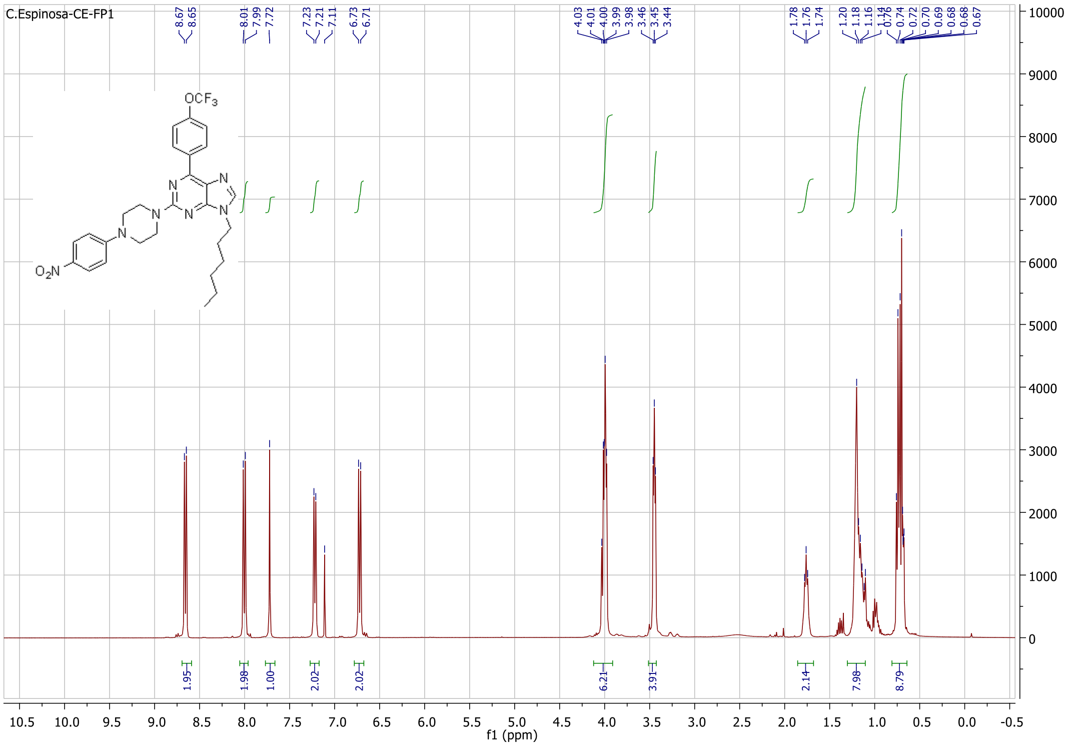

^{13}C NMR of compound **4o**

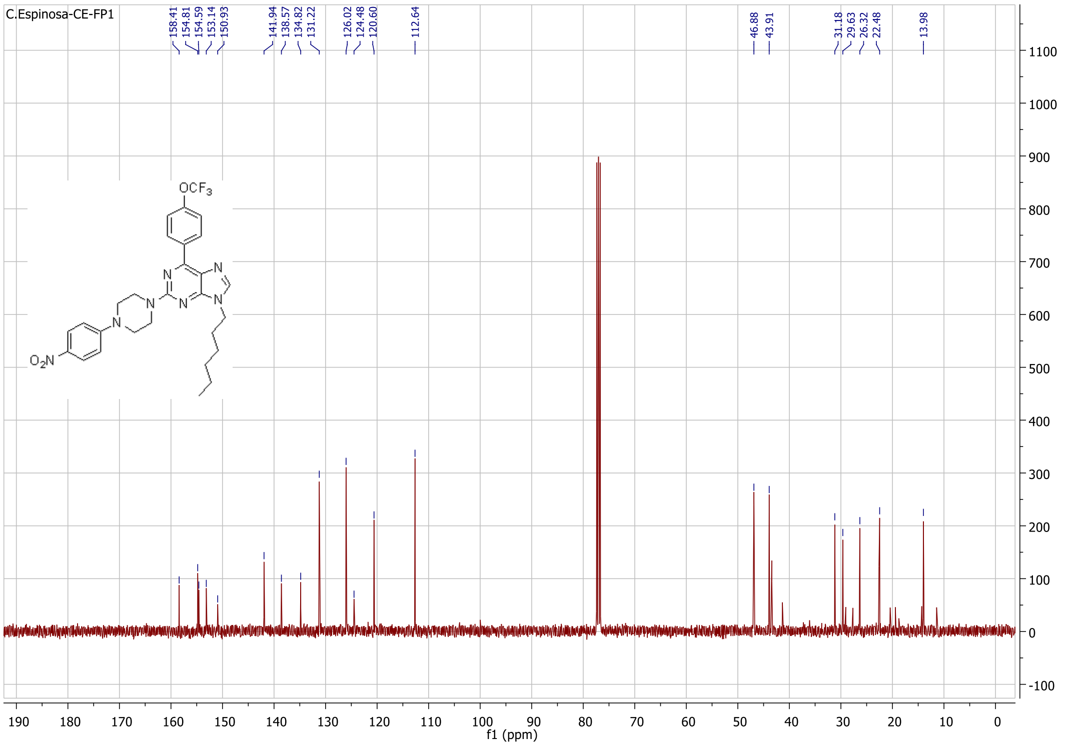

^{1}H NMR of compound **4p**

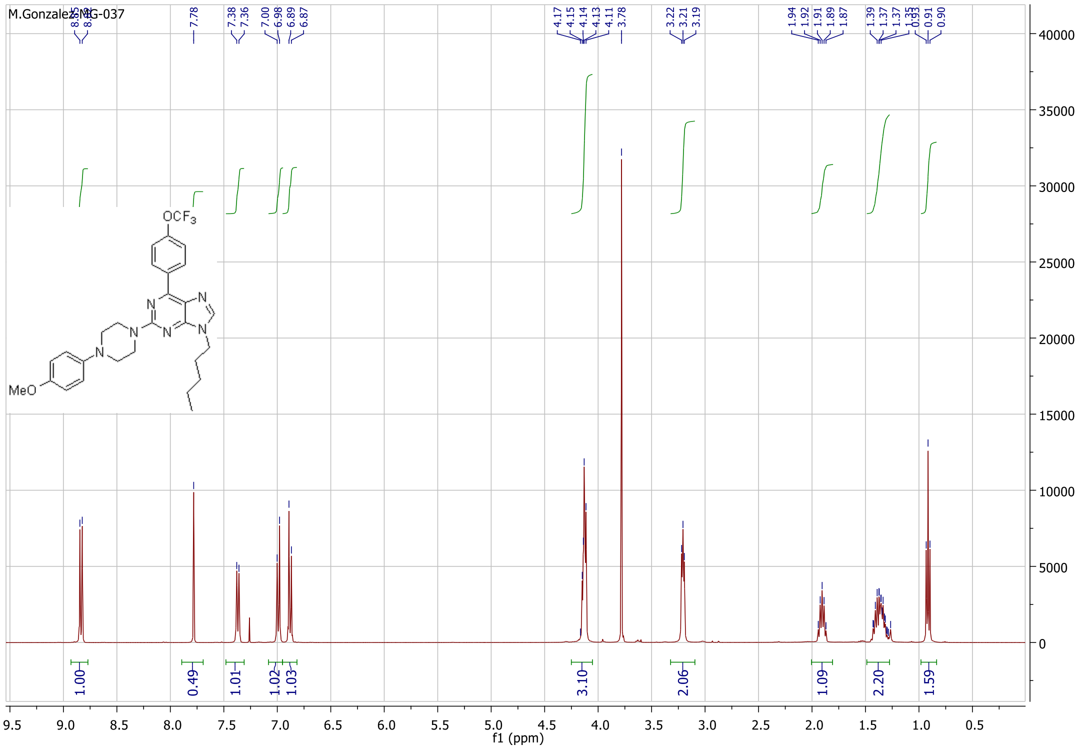

^{13}C NMR of compound **4p**

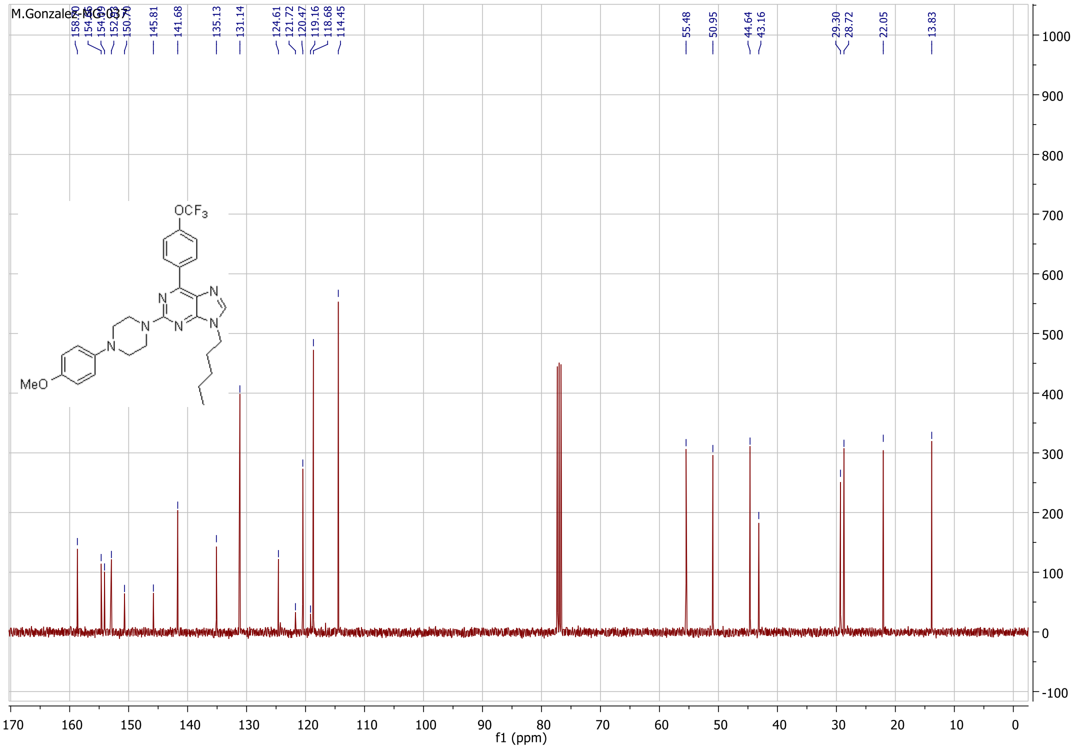

^{1}H NMR of compound **4q**

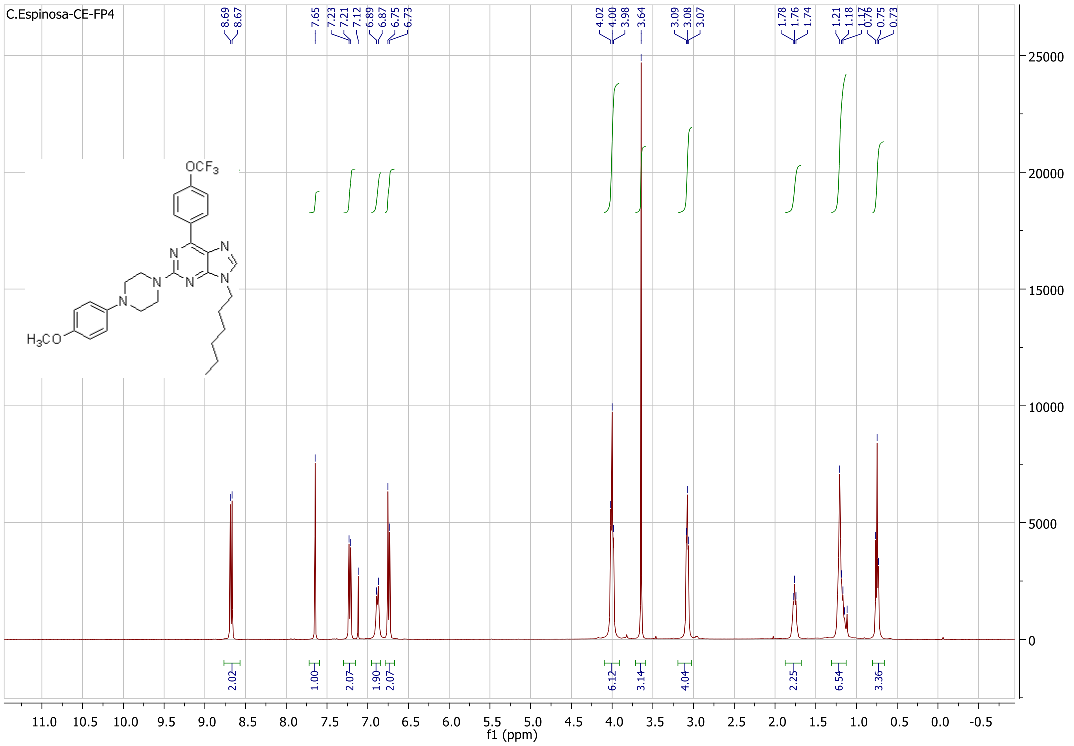

^{13}C NMR of compound **4q**

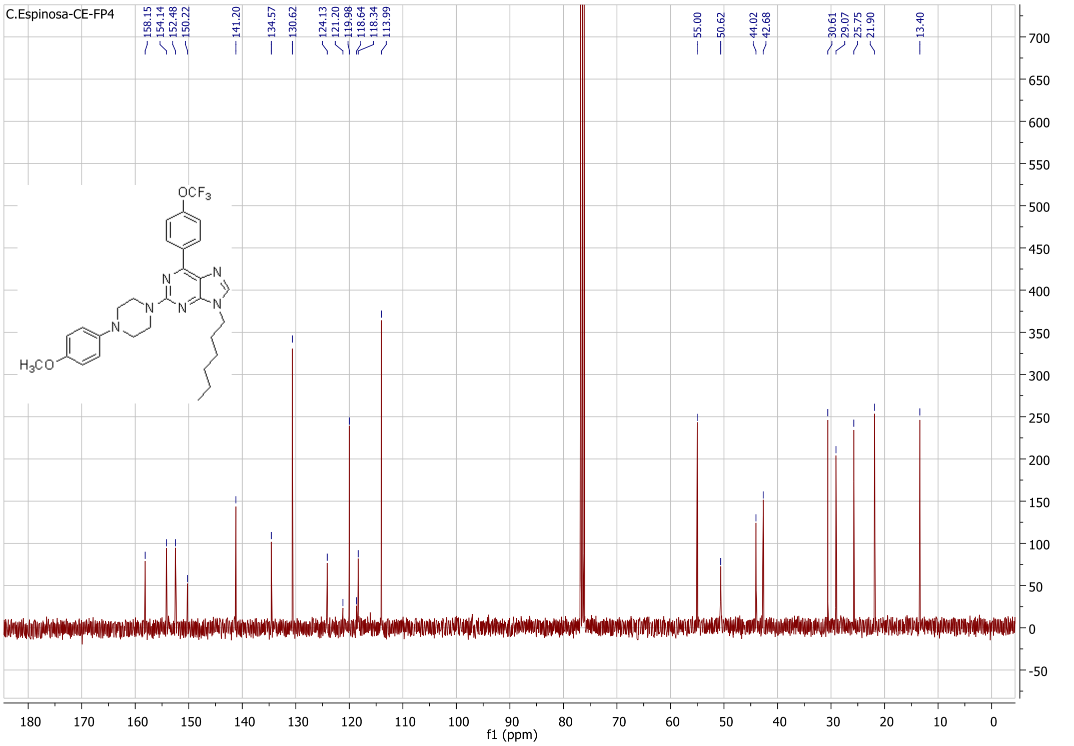

^{1}H NMR of compound **4r**

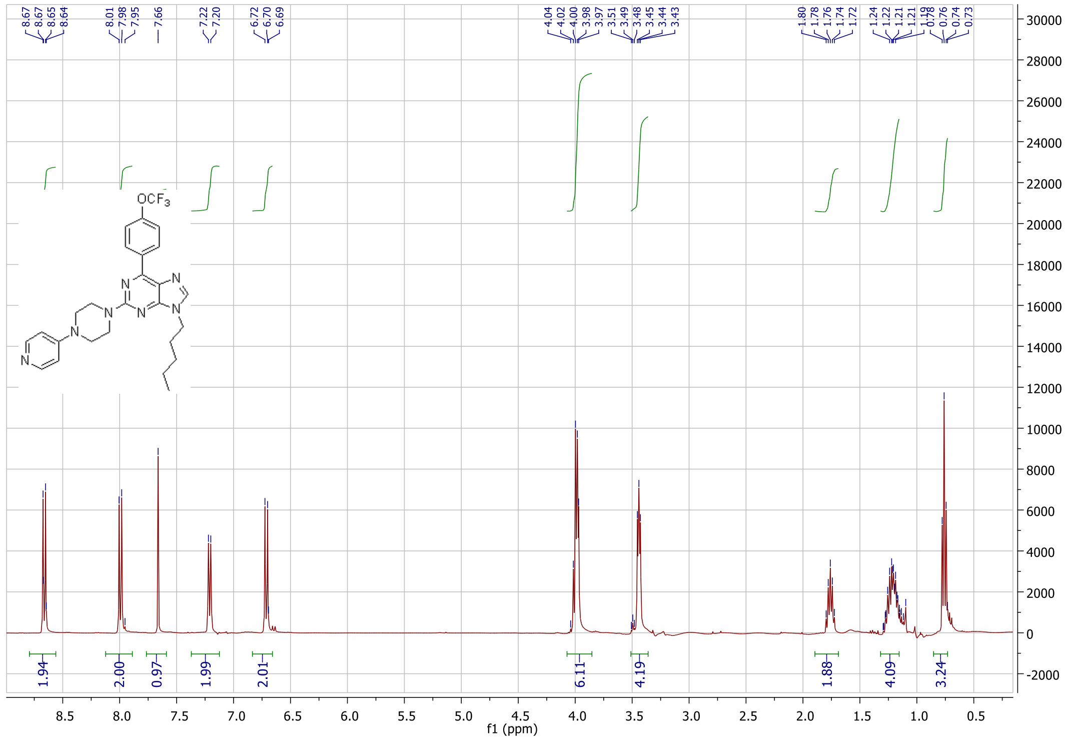

^{13}C NMR of compound **4r**

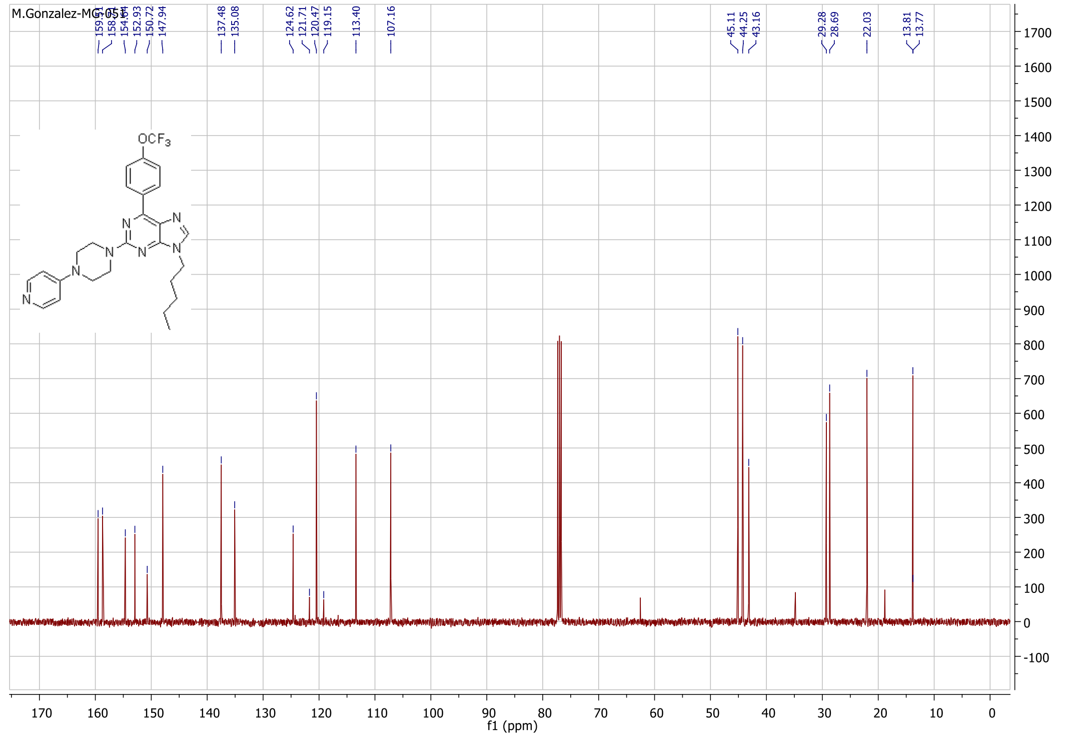

^{1}H NMR of compound **4s**

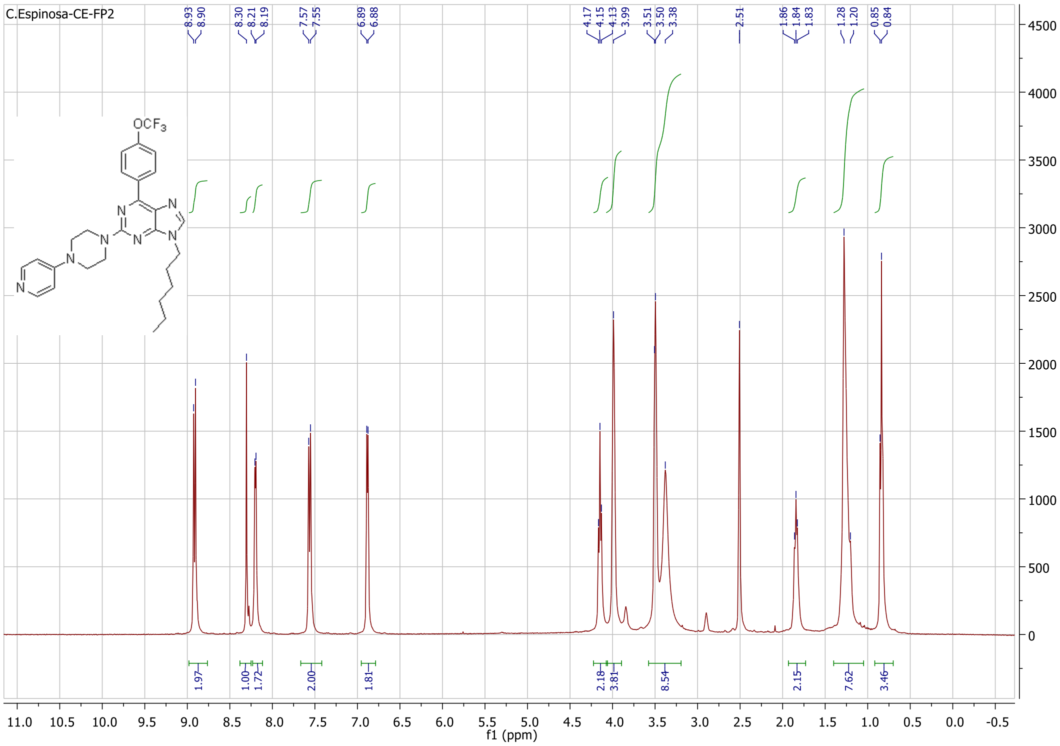

^{13}C NMR of compound **4s**

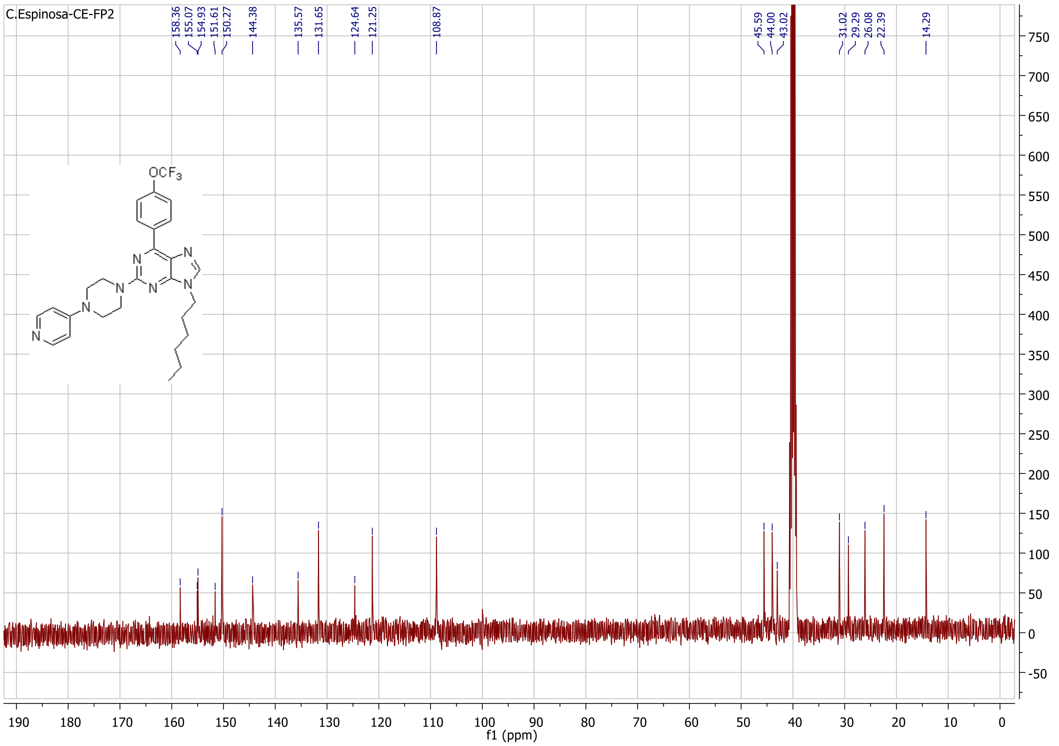

Mass spectrum of compound **4a**

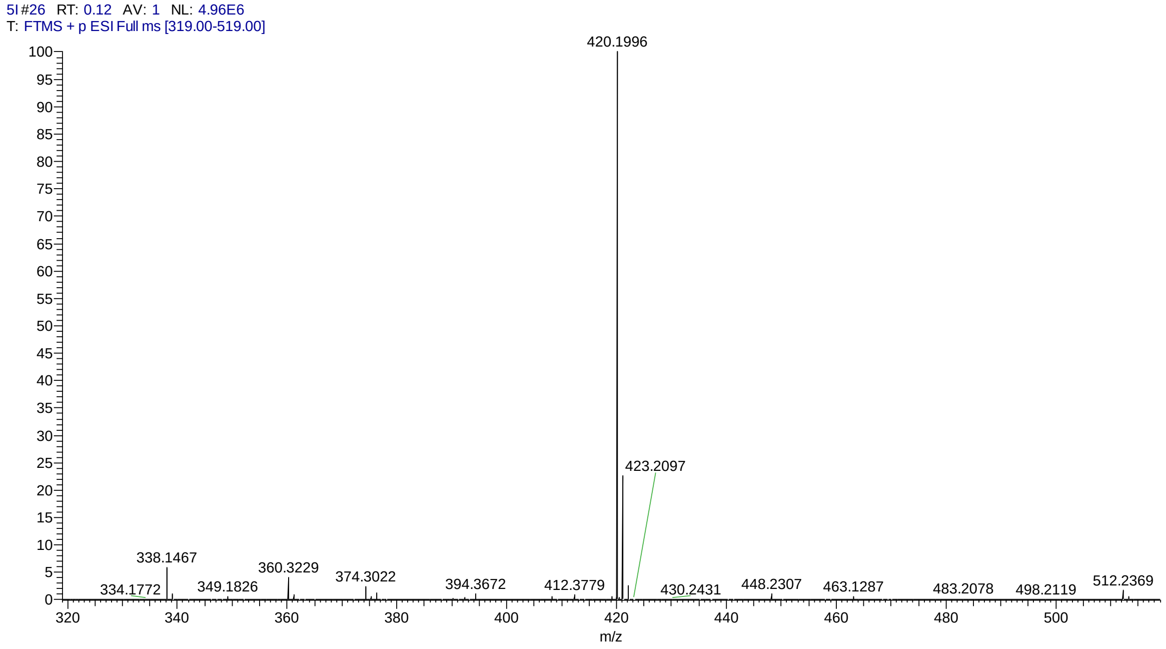

Mass spectrum of compound **4b**

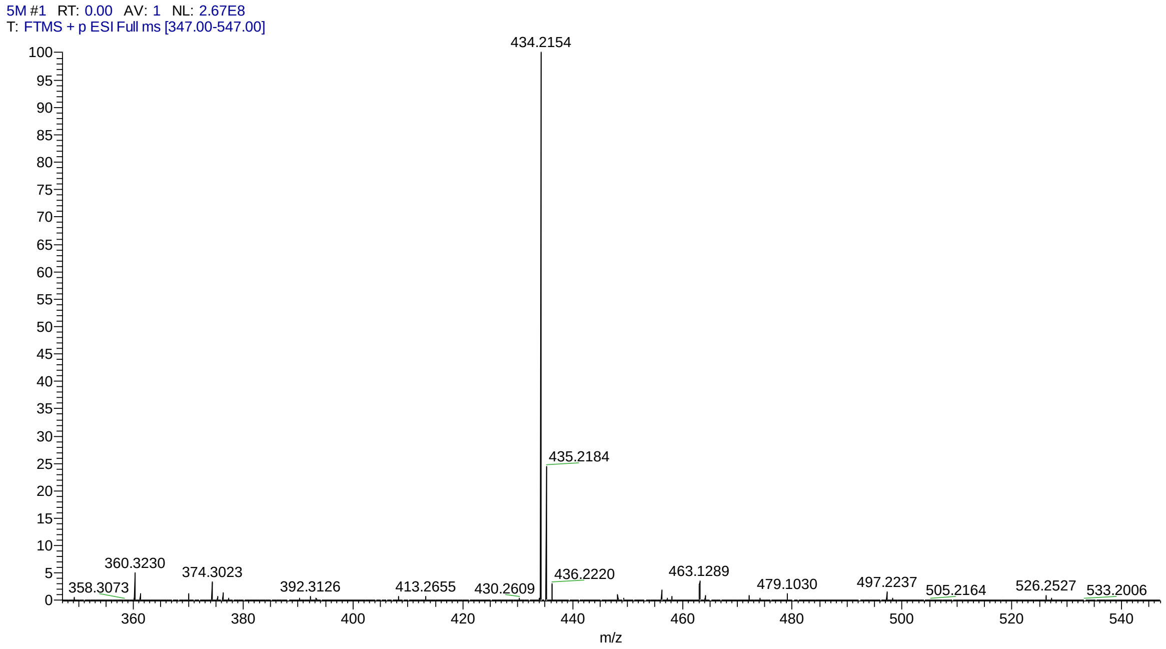

Mass spectrum of compound **4c**

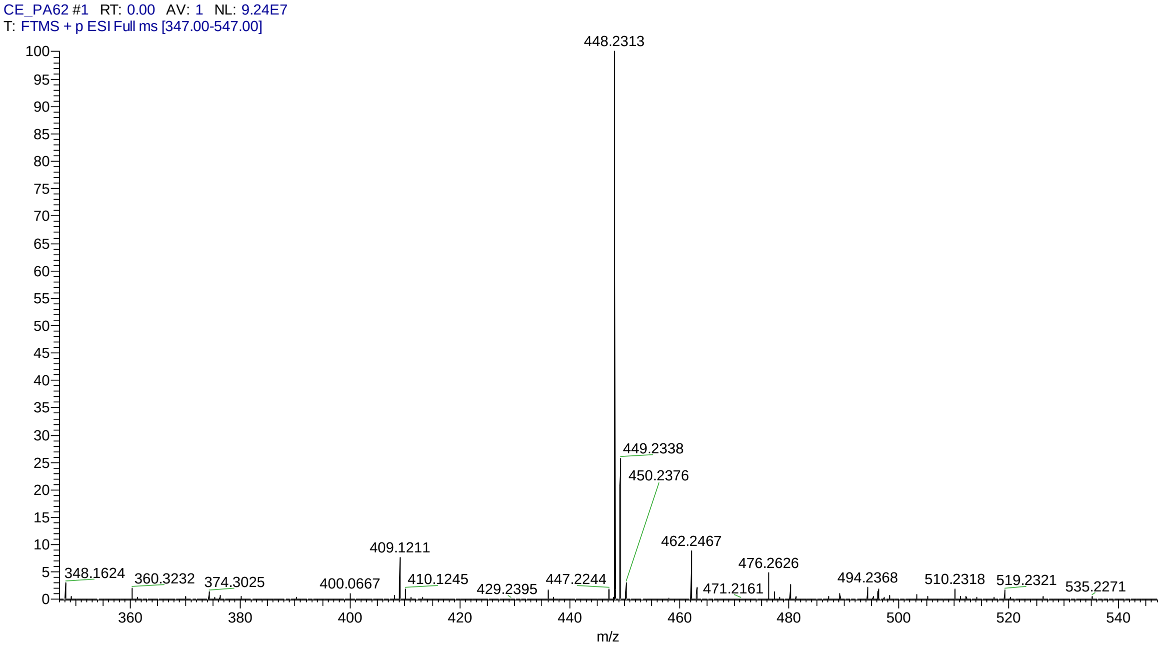

Mass spectrum of compound **4d**

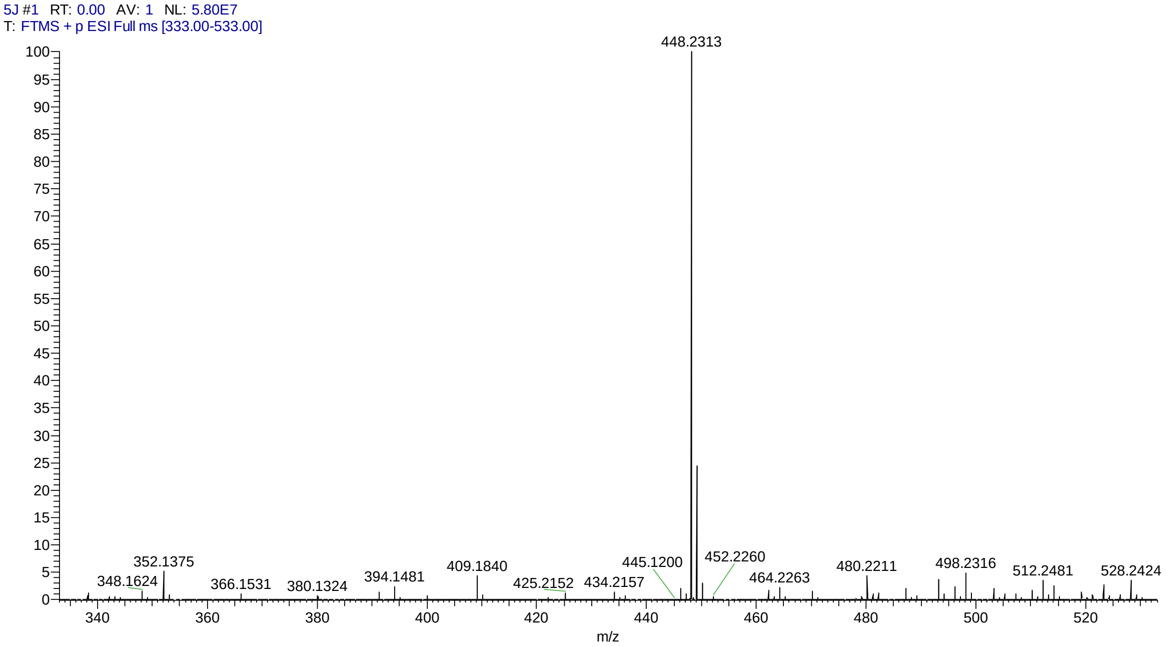

Mass spectrum of compound **4e**

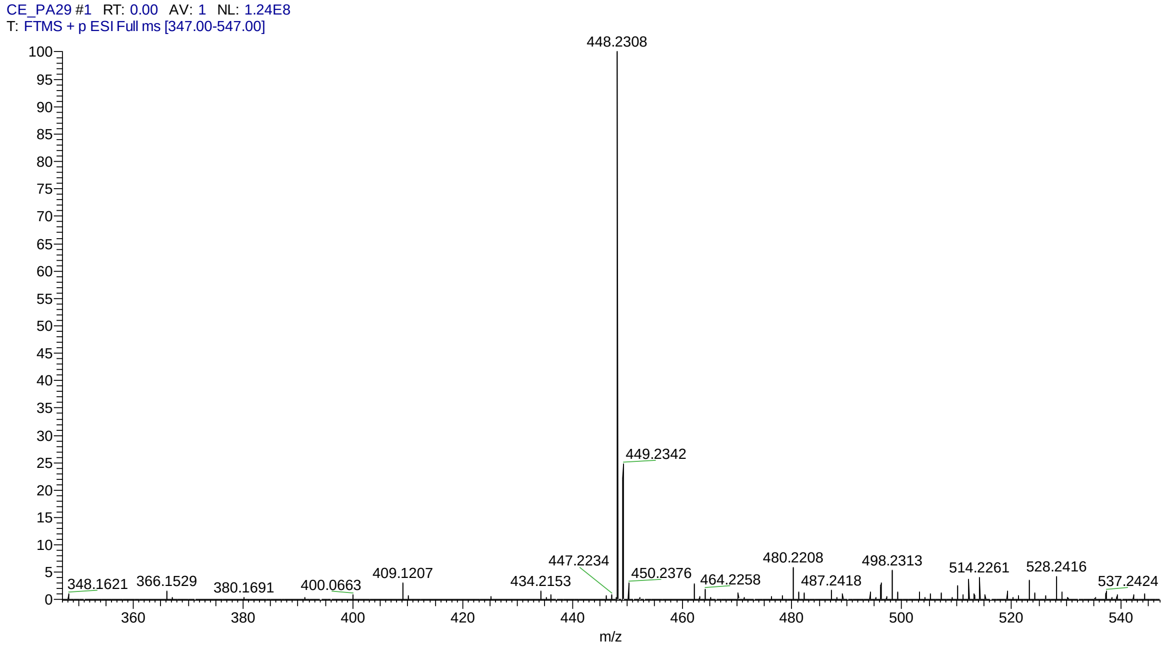

Mass spectrum of compound **4f**

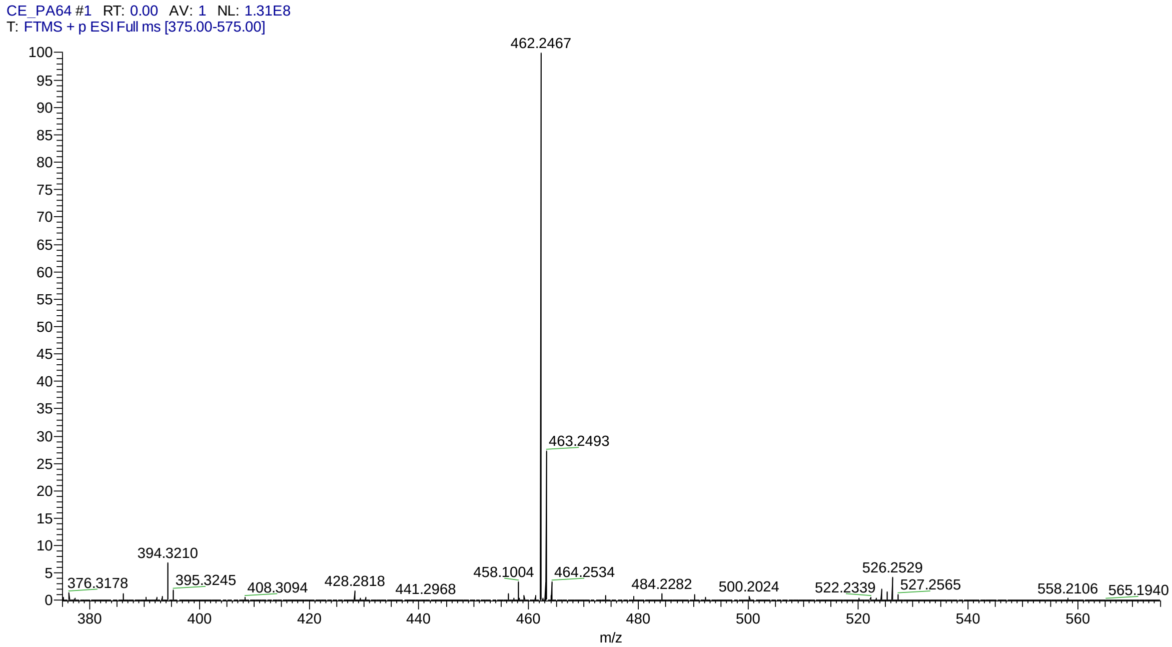

Mass spectrum of compound **4g**

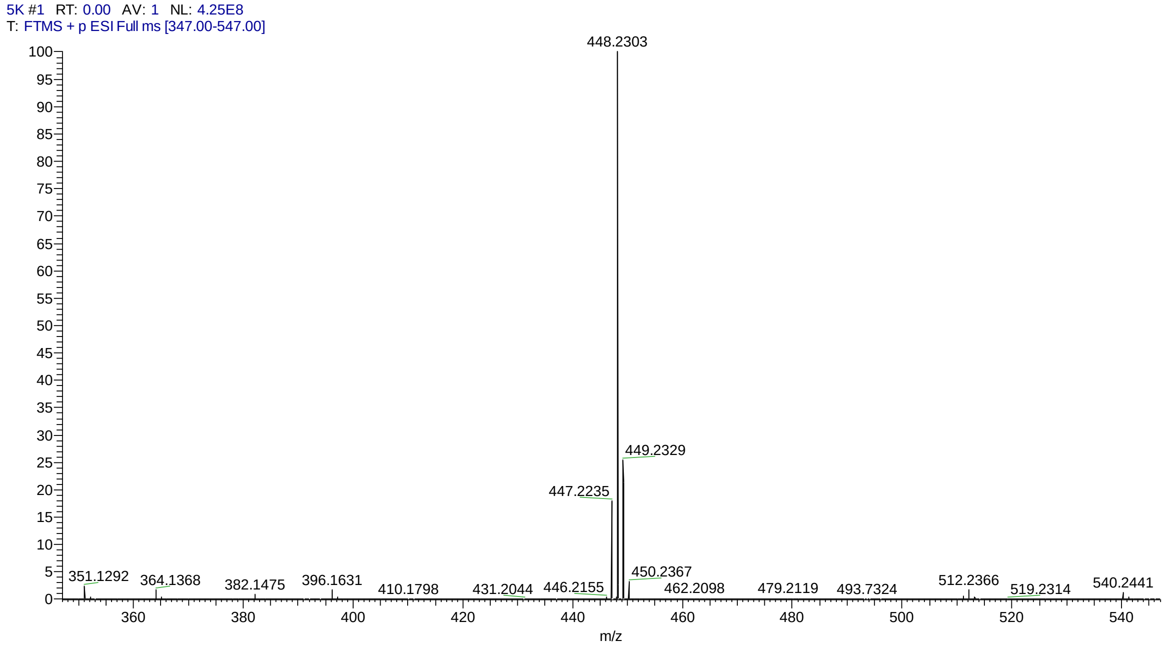

Mass spectrum of compound **4h**

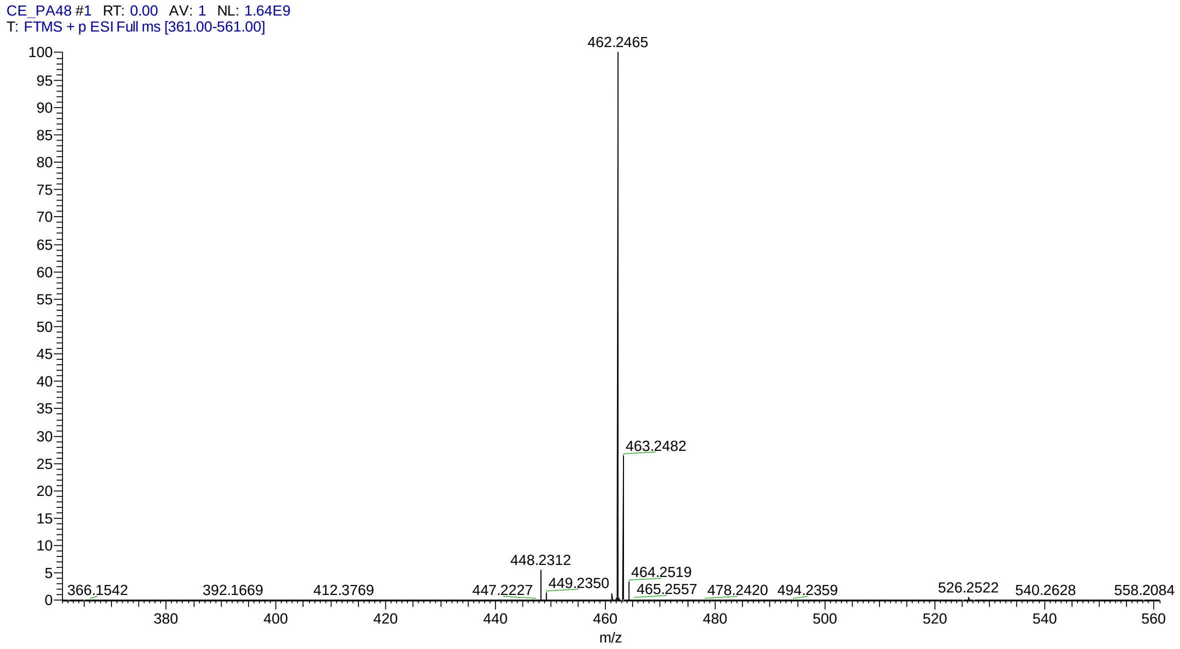

Mass spectrum of compound **4i**

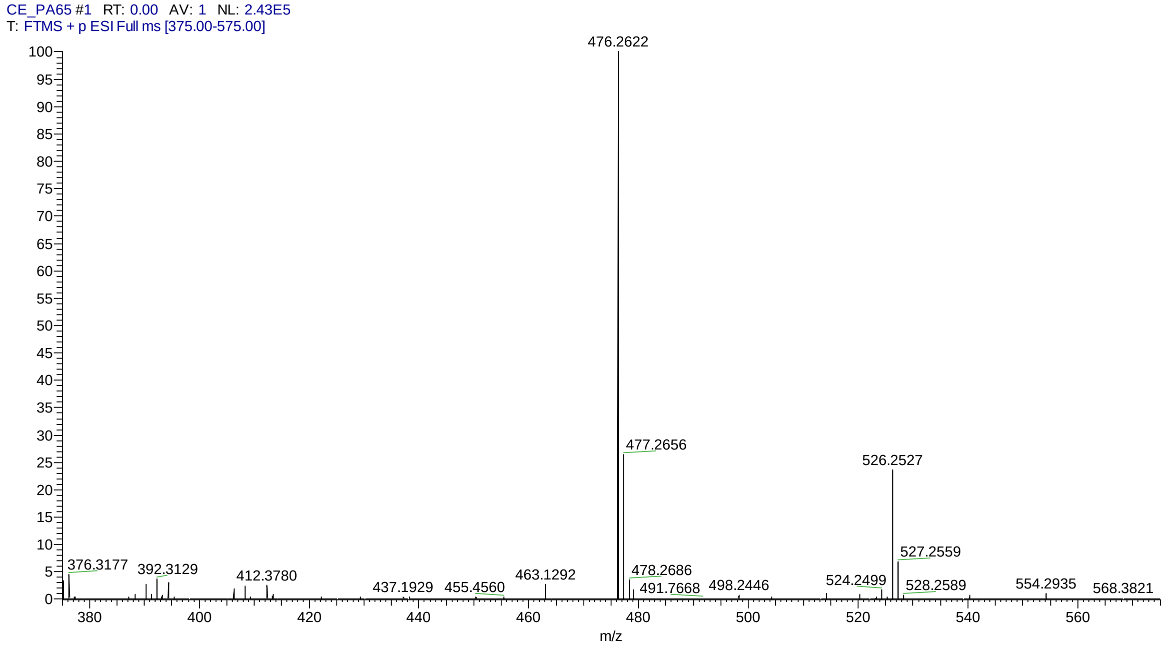

Mass spectrum of compound **4j**

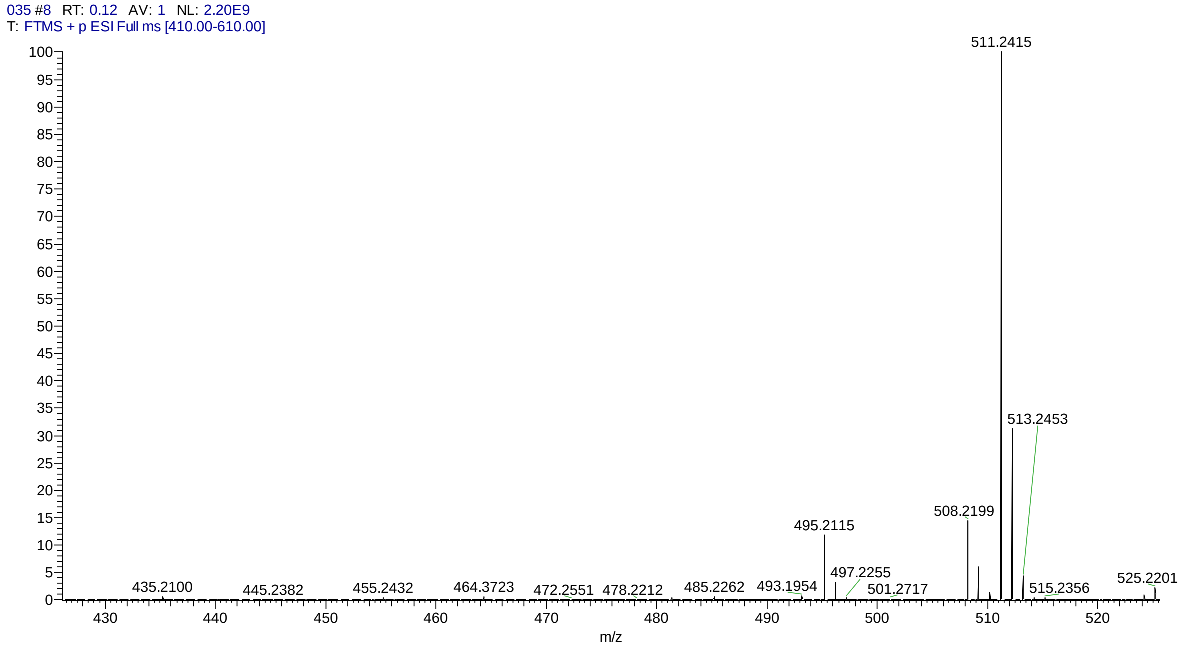

Mass spectrum of compound **4k**

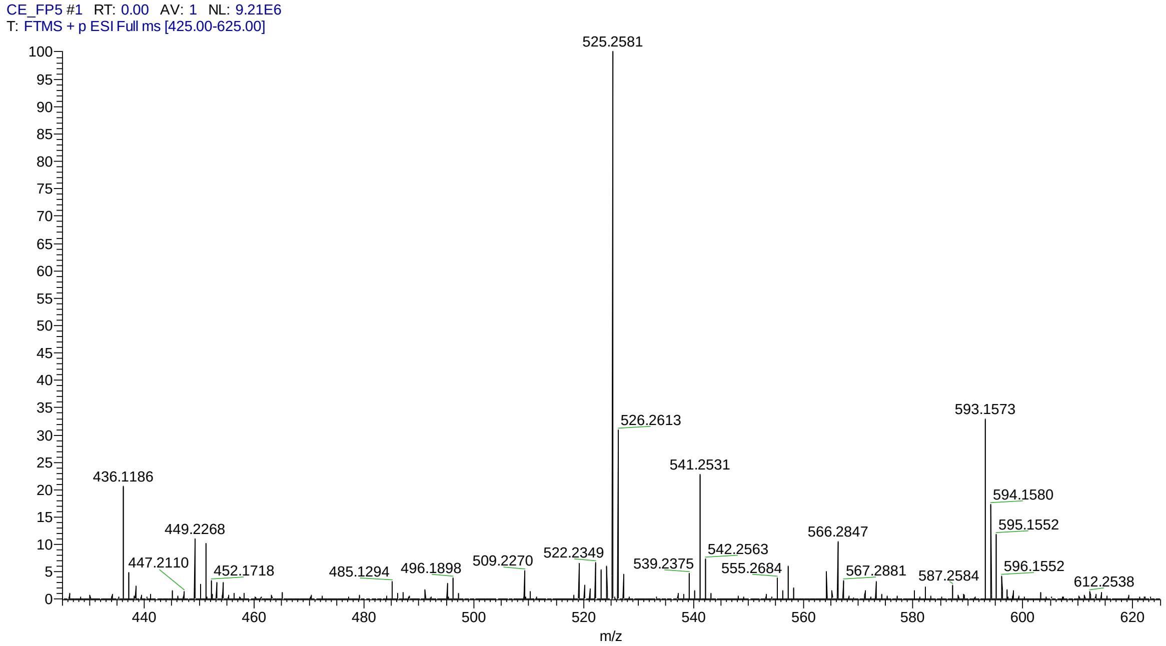

Mass spectrum of compound **4l**

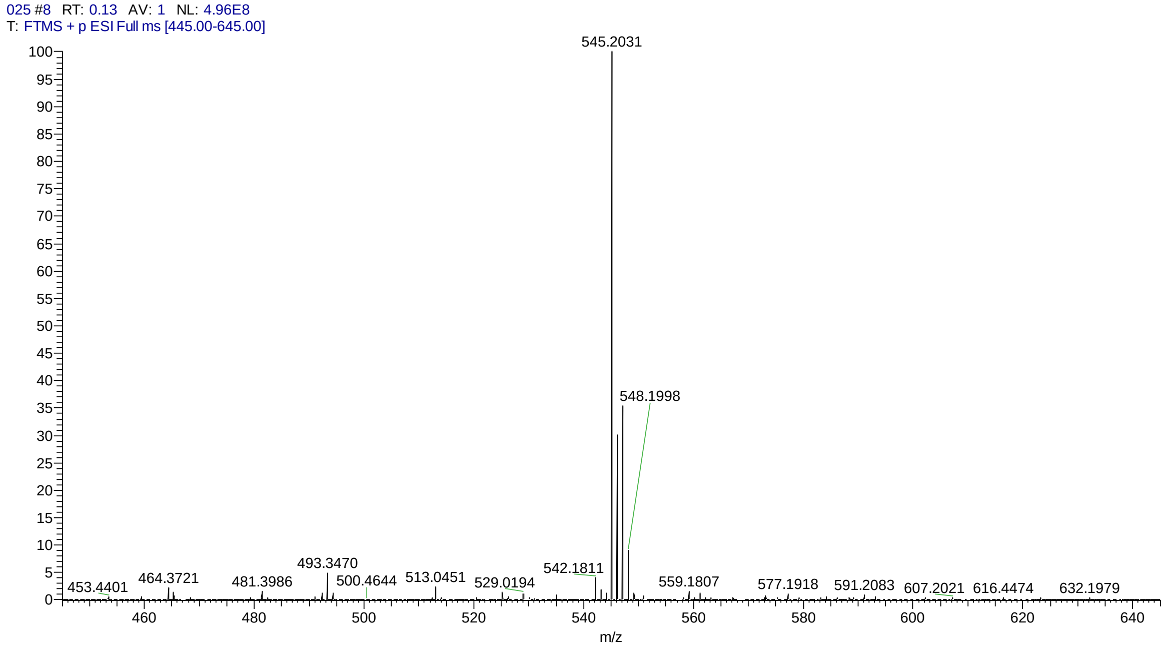

Mass spectrum of compound **4m**

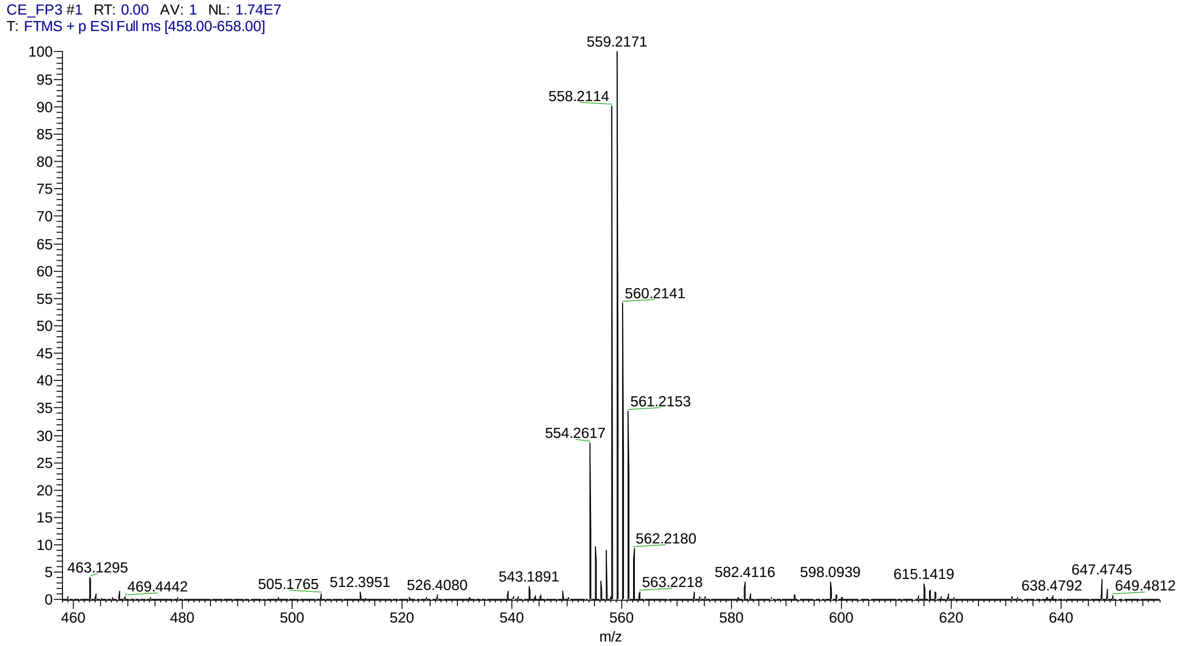

Mass spectrum of compound **4n**

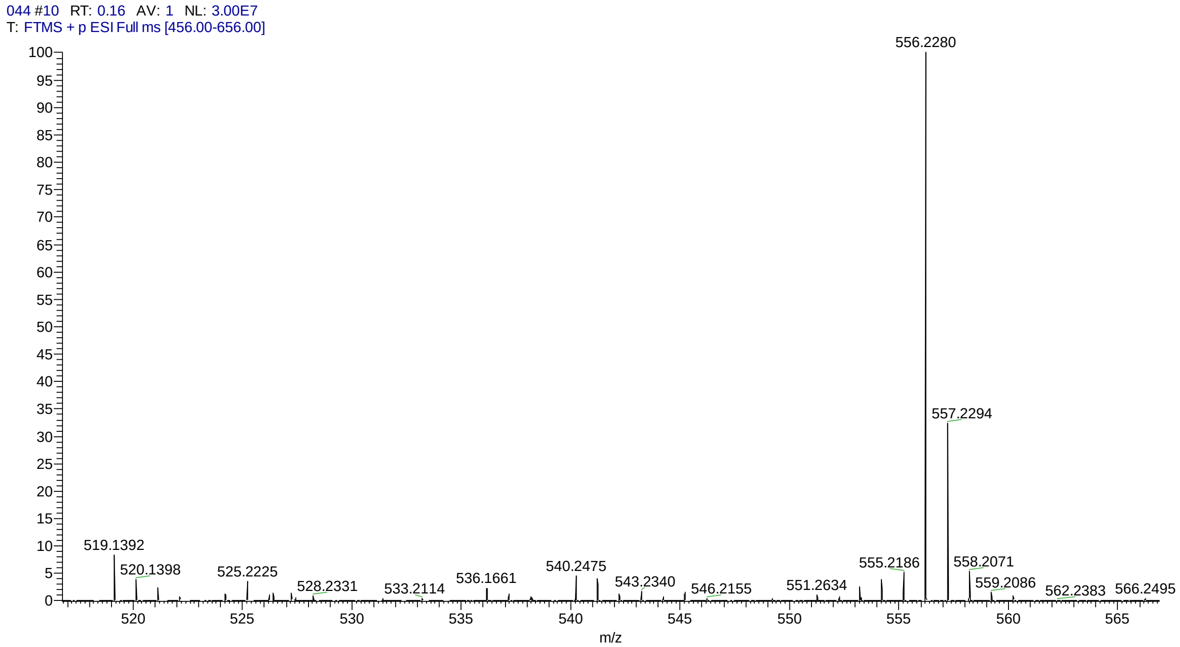

Mass spectrum of compound **4o**

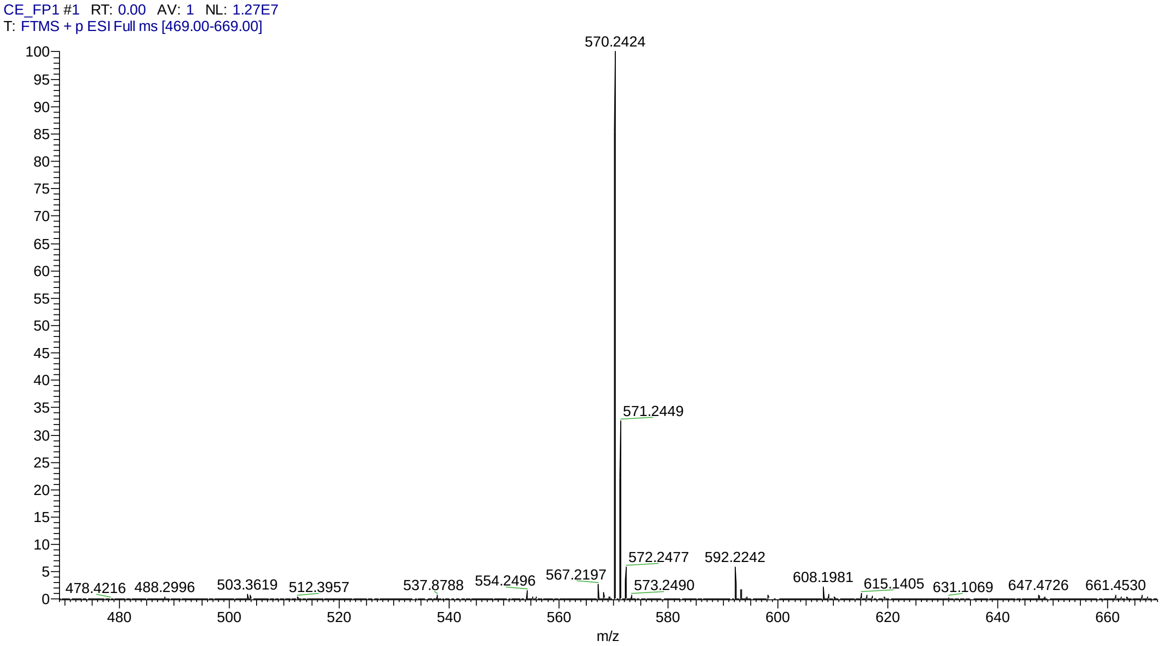

Mass spectrum of compound **4p**

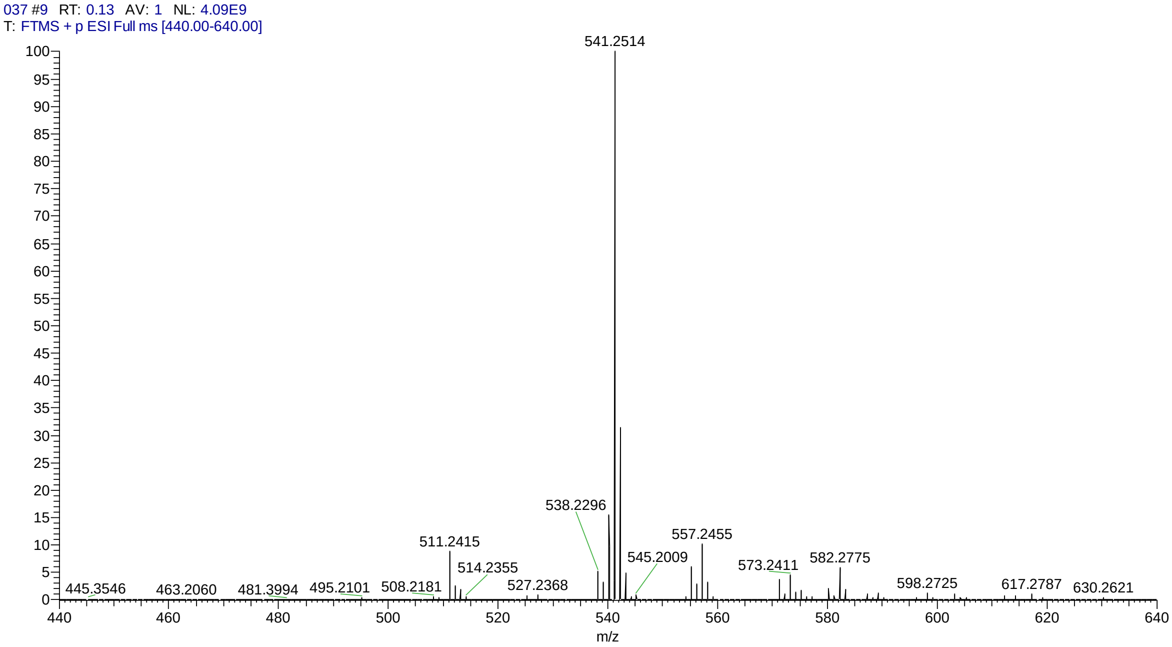

Mass spectrum of compound **4q**

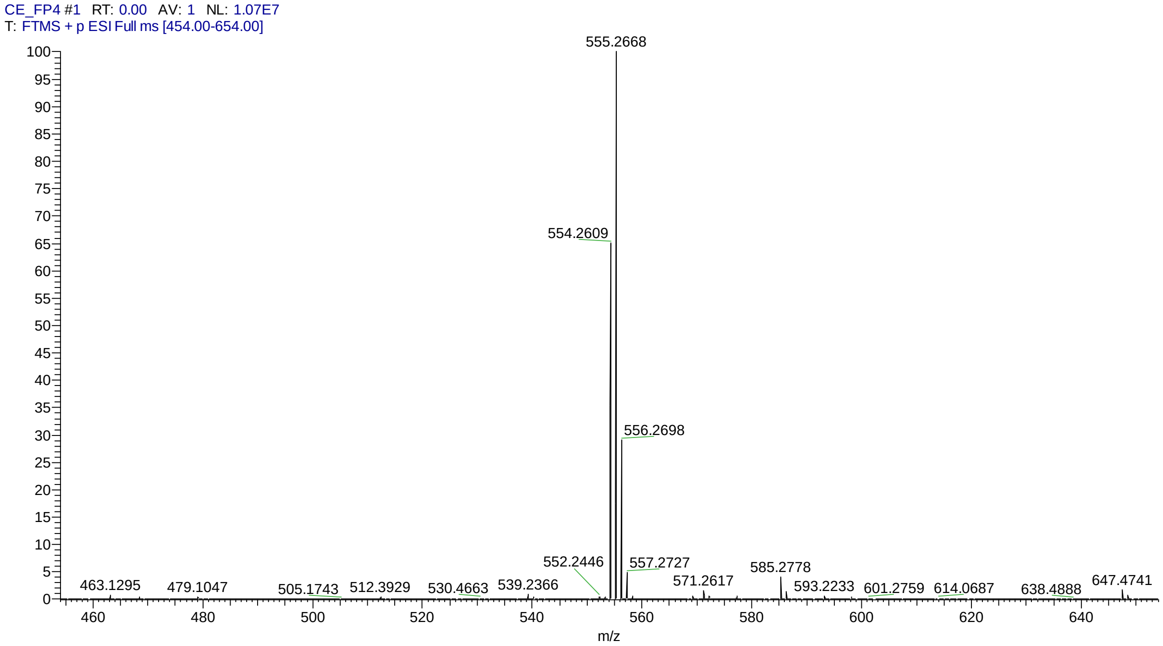

Mass spectrum of compound **4r**

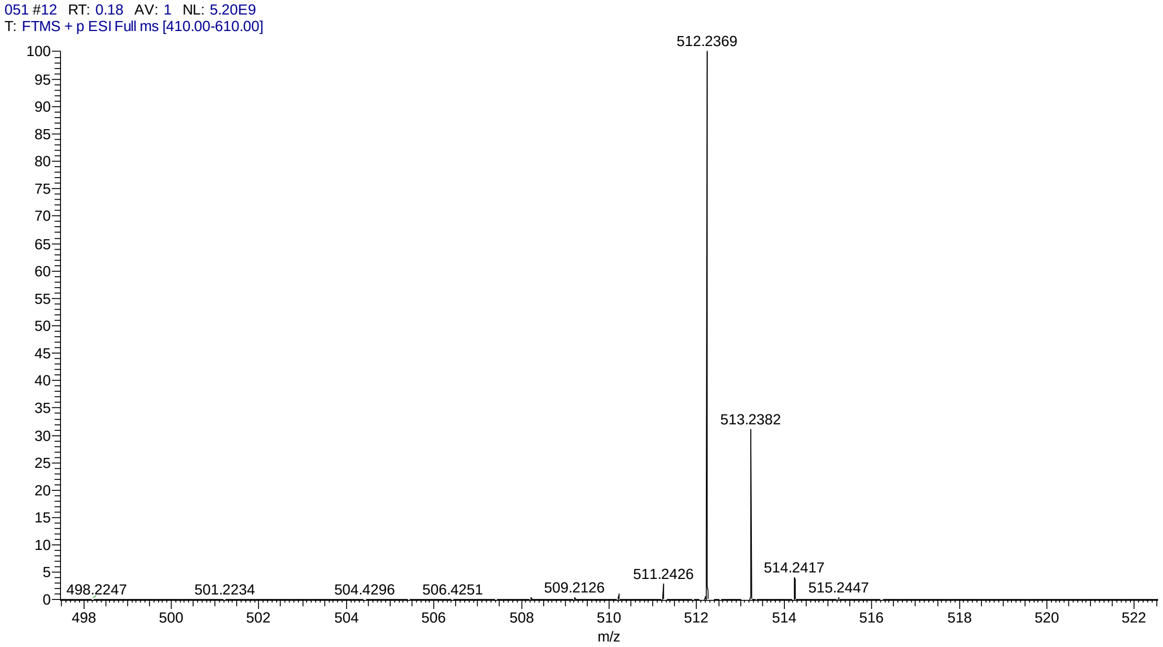

Mass spectrum of compound **4s**

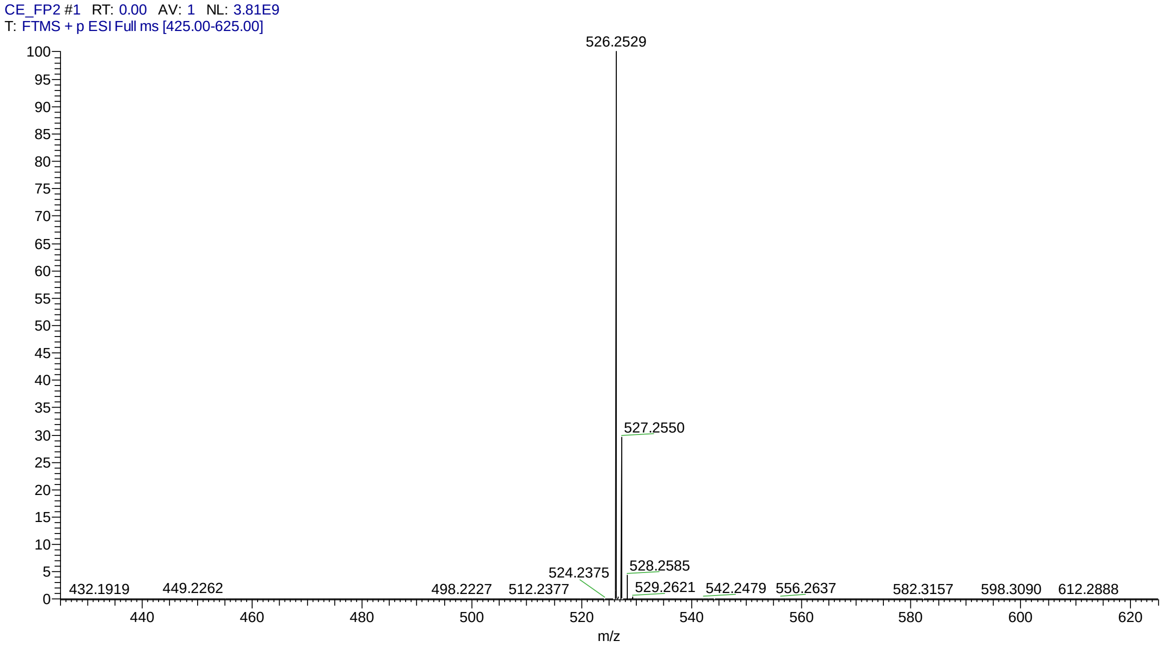

Supplemental Table S1: In vitro cytotoxicity of purine derivatives 4a-s at 50 µM on four cancer cell lines and control cells (HEK293).

Compound	% Cytotoxicity (50 µM)				
	**HCT116**	**HT29**	**H1975**	**DAOY**	**HEK293**
**4a**	68±0.3	<50	66±0.9	82±6.2	<50
**4b**	53±0.9	<50	30±1.0	94±0.4	<50
**4c**	<50	<50	19±0.8	69±0.9	<50
**4d**	<50	<50	<50	<50	<50
**4e**	<50	<50	<50	<50	<50
**4f**	<50	<50	<50	<50	<50
**4g**	<50	<50	<50	<50	<50
**4h**	<50	<50	61±1.2	81±1.1	<50
**4i**	<50	<50	<50	<50	<50
**4j**	<50	<50	<50	<50	<50
**4k**	<50	<50	<50	<50	<50
**4l**	<50	<50	<50	<50	<50
**4m**	<50	<50	<50	<50	<50
**4n**	<50	<50	<50	<50	<50
**40**	<50	<50	<50	<50	<50
**4p**	<50	<50	<50	<50	<50
**4q**	<50	<50	<50	<50	<50
**4r**	81±0.8	<50	<50	92±0.4	<50
**4s**	99±0.4	25±2.7	99±0.2	98±0.3	<50
**Vismodegib**	<50	<50	53±1.2	<50	<50
**Etoposide**	90±0.5	-	60±1.1	-	65±0.7
**Cisplatin**	-	-	-	88±0.7	88±0.7
**5-FU**	62±1.3	66±4.9-	-	-	<50

A)

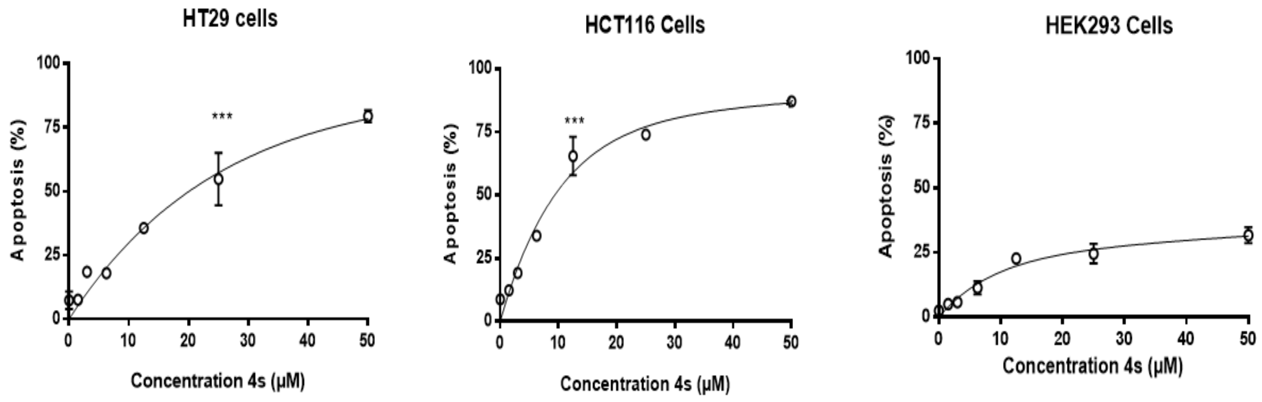

B)

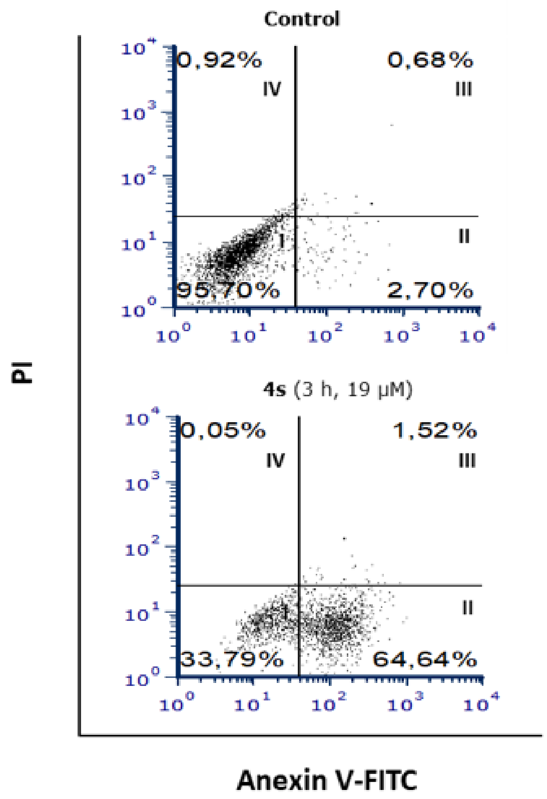

Supplemental Figure S1: 4s triggers apoptosis in various cancer cell lines. **A)** Dose-response curves are shown for four neoplastic cancer cell lines and HEK293 cells as a control. Cells were treated with **4s** at 1.5, 3.0, 6.25, 19, 25 and 50 µM for 72 h, stained with annexin V-FITC and propidium iodide (PI) and analyzed by flow cytometry. Results are presented as the mean ± SD of three independent experiments. (***) $p < 0.0001$ vs. control. Data were analyzed with a non-parametric *t*-test. **B)** Representative dot plot of HT29 cells untreated (control) or treated for 3 h with **4s** (19 µM).

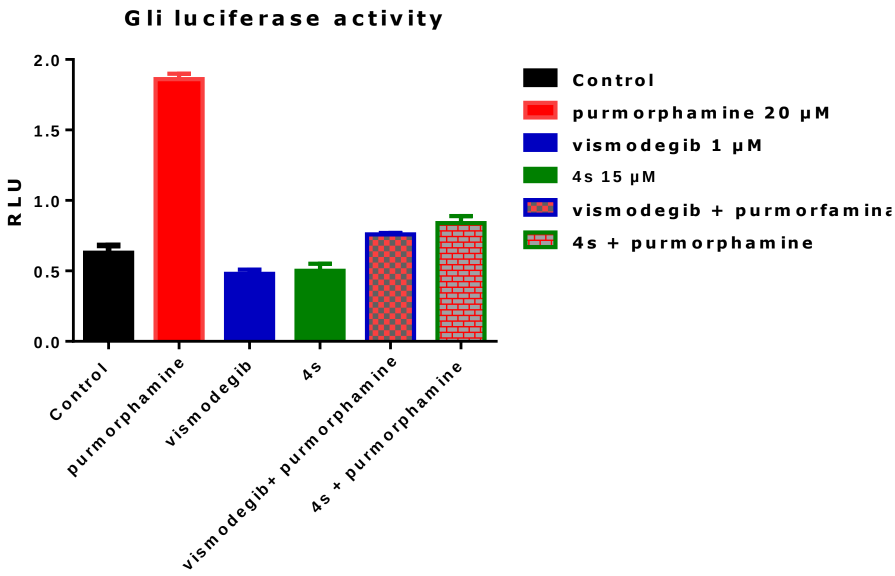

Supplemental Figure S2: **Luciferase reporter activity dependent on the Hh pathway.** This assay was performed in HCT116 cells stably transformed with a Gli responsive luciferase reporter gene and a renilla luciferase gene (normalization control), treated for 24 h with purmorphamine (20 µM), vismodegib (1 µM) or **4s** (1.5 µM). These concentrations were 100 times higher than the IC_{50} values reported for each compound. Luciferase and renilla activity were assayed with the dual-luciferase assay system Cignal Reporter Gli-luciferase (ADN, Qiagen). Results are expressed as luciferase/renilla activity ratios and represent the mean of triplicate values from one assay.

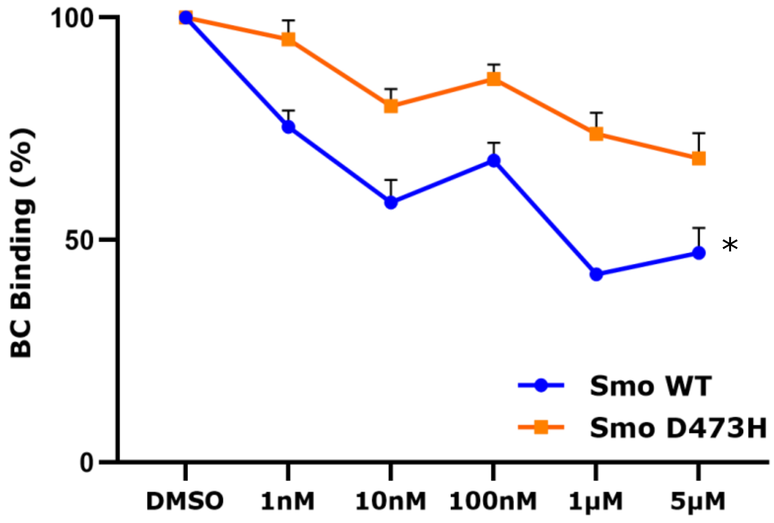

Supplemental Figure S3: **Binding of 4s to wild-type and D473H mutant SMO cells**. The concentration-response curves represent the percentage of BODIPY-cyclopamine (BC) incorporation into control HEK293T cells carrying either the wild type SMO gene (Smo WT, in blue) or the D473H mutant SMO gene (SMO-D473H, in orange) after treatment with **4s**. IC_{50} Smo WT = 0.95 µM. At concentrations greater than 5 µM, **4s** was toxic to SMO D473H. Data represent the average ± SD of three independent experiments. Data are the average BC intensity of five fluorescence microscopy images.

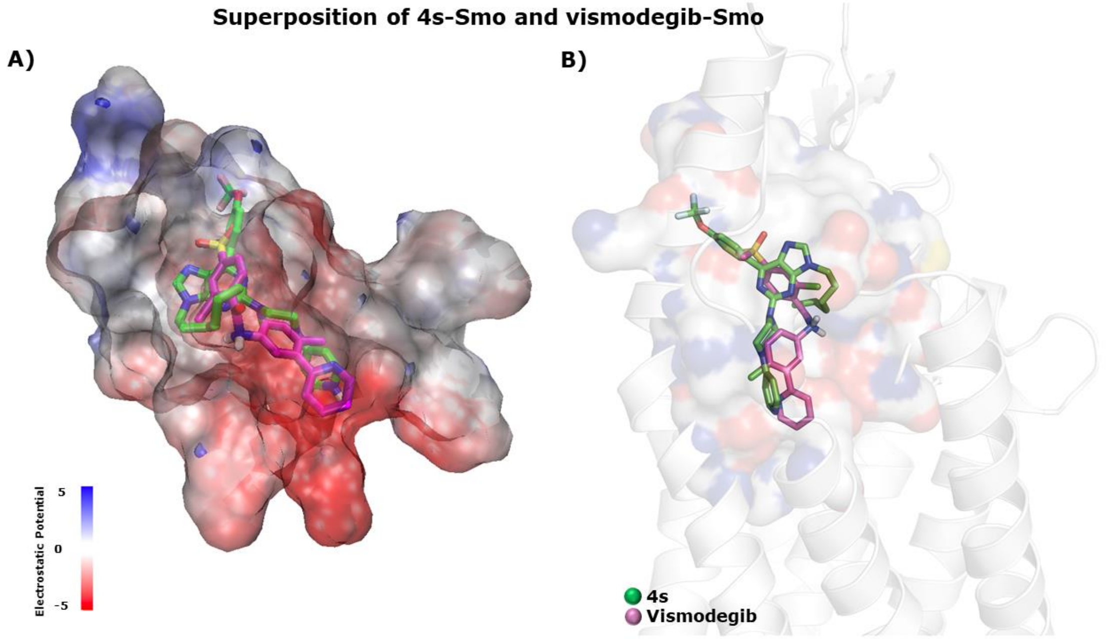

Supplemental Figure S4: **Graphical superposition of 4s-SMO (green) and vismodegib-SMO (fuchsia). A)** Electrostatic potential. The binding cavity (in red) provides a highly electron-rich environment. **B)** 3D representation of **4s** and vismodegib binding to the inner part of the heptahelical bundle of SMO.

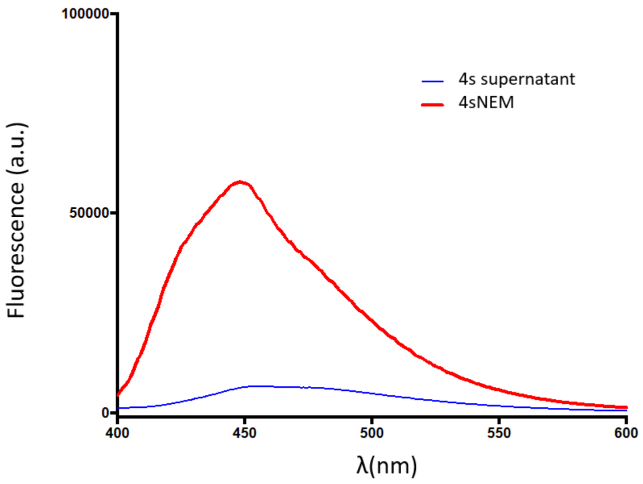

Supplemental Figure S5: Fluorescence spectra of 4s and 4sNEM. The fluorescence was detected with the excitation wavelength 365 nm. Red indicates **4s** incorporate in NEM (**4s-NEM**) and blue indicates **4s** detected in supernatant.

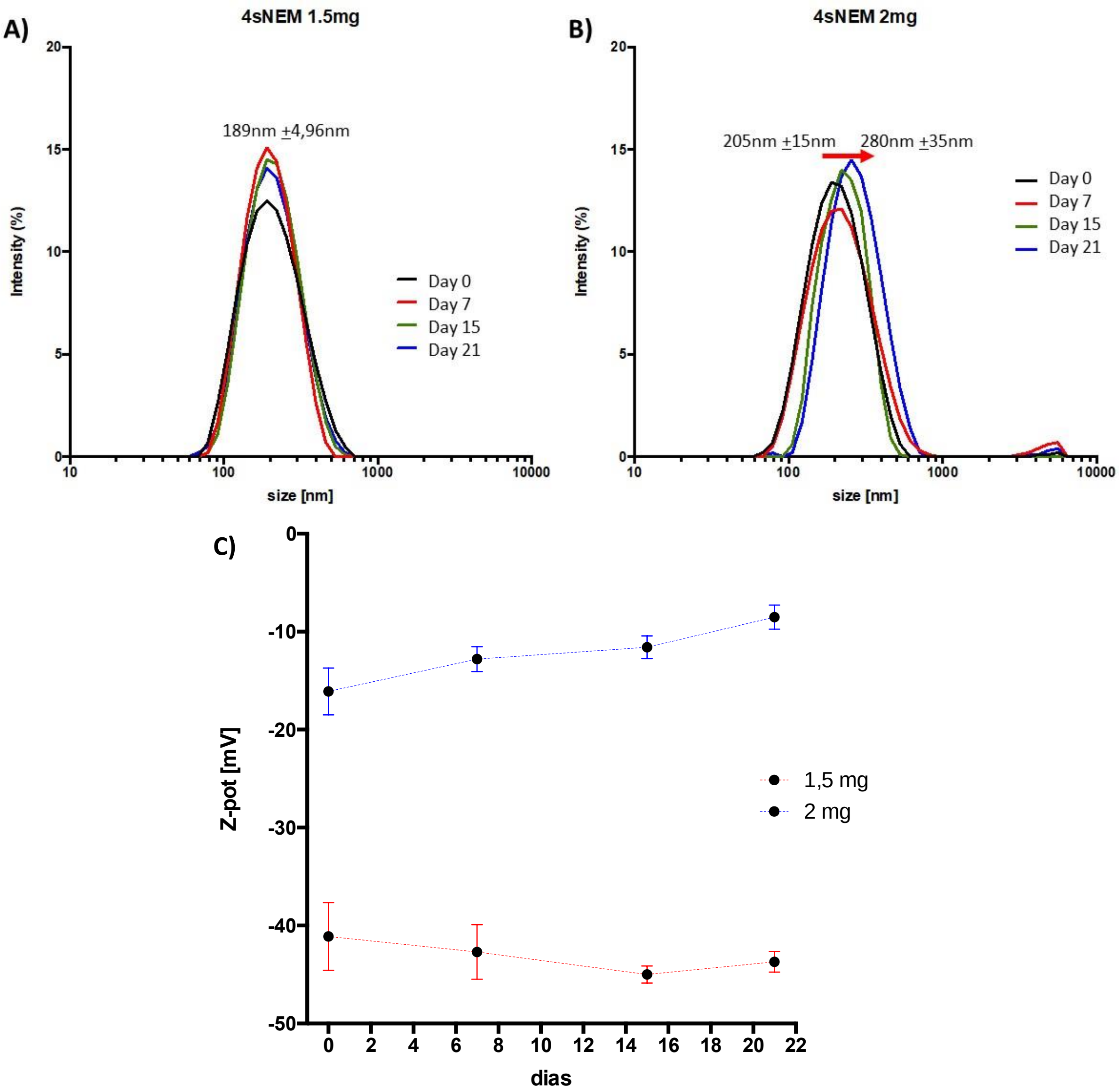

Supplemental Figure S6: Development of a formulation to nanoemulsify the SMO antagonist 4s. The stability of two systems using different amounts of **4s** considering the hydrodynamic diameter: **A**)1.5 mg and **B**) 2 mg, both evaluated for 21 d. **C**) The Z potential were measured in triplicate.

CSM: polen Series: 1957 Report Name: modified System: Chromaster DAD

Chromaster System Manager Report

Analyzed Date and Time: 04-12-2020 17:18 Reported Date and Time: 04-12-2020 17:45:49

Processed Date and Time: 04-12-2020 17:32

Data Path: C:\WIN32APP\CHROMASTER\polen\DATA\1957\

Processing Method: Christian

System (acquisition): Chromaster DAD Series: 1957

Application(data): polen Vial Number: 2

Sample Name: 4sA Vial Type: UNK

Injection from this vial: 1 of 1 Volume: 5,0 ul

Sample Description:

Chrom Type: Fixed WL Chromatogram, 260 nm

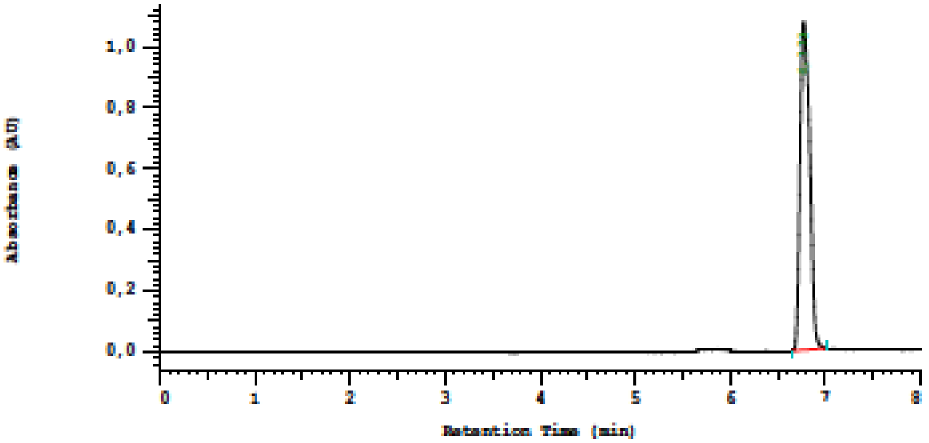

Processing Method: Christian

Method Developer: Paula

Pump 1: 5160

Pump 1 Solvent A: Pump 1 Solvent B:

Pump 1 Solvent C: MeCN/formico Pump 1 Solvent D: agua/formico

Method Description:

Chrom Type: Fixed WL Chromatogram, 260 nm

Peak Quantitation: AREA

Calculation Method: AREA%

No.	RT	Area	Conc 1	BC
1	6,773	3782079	100,000	BB
		3782079	100,000	

Peak rejection level: 0

Supplemental Figure S7: HPLC spectrum for 4s.

Supplemental Table S2: PAIN´s report for purine derivatives **4a-s**. This analysis was done by Swiss Target Prediction platform (http://www.swisstargetprediction.ch/).

Compounds	PAIN
**4a**	0
**4b**	0
**4c**	0
**4d**	0
**4e**	0
**4f**	0
**4g**	0
**4h**	0
**4i**	0
**4j**	0
**4k**	0
**4l**	0
**4m**	0
**4n**	0
**4o**	0
**4p**	0
**4q**	0
**4r**	0
**4s**	0
